# Dynamic and solid-state behaviour of bromoisotrianglimine†

**DOI:** 10.1039/d4sc04207g

**Published:** 2024-08-13

**Authors:** Abbie M. Scholes, Laurence J. Kershaw Cook, Filip T. Szczypiński, Konstantin V. Luzyanin, Benjamin D. Egleston, Rebecca L. Greenaway, Anna G. Slater

**Affiliations:** a Department of Chemistry and Materials Innovation Factory, School of Physical Sciences, University of Liverpool UK anna.slater@liverpool.ac.uk; b Department of Chemistry, Molecular Sciences Research Hub Imperial College London London UK

## Abstract

Solid-state materials formed from discrete imine macrocycles have potential in industrial separations, but dynamic behaviour during both synthesis and crystallisation makes them challenging to exploit. Here, we explore opportunities for structural control by investigating the dynamic nature of a C-5 brominated isotrianglimine in solution and under crystallisation conditions. In solution, the equilibrium between the [3 + 3] and the less reported [2 + 2] macrocycle was investigated, and both macrocycles were fully characterised. Solvent templating during crystallisation was used to form new packing motifs for the [3 + 3] macrocycle and a previously unreported [4 + 4] macrocycle. Finally, chiral self-sorting was used to demonstrate how crystallisation conditions can not only influence packing arrangements but also shift the macrocycle equilibrium to yield new structures. This work thus exemplifies three strategies for exploiting dynamic behaviour to form isotrianglimine materials, and highlights the importance of understanding the dynamic behaviour of a system when designing and crystallising functional materials formed using dynamic covalent chemistry.

## Abstract 2

The dynamic behaviour of simple imine macrocycles under crystallisation conditions is exploited to form new structural motifs, including a previously unreported [4 + 4] macrocycle.

## Graphical abstract

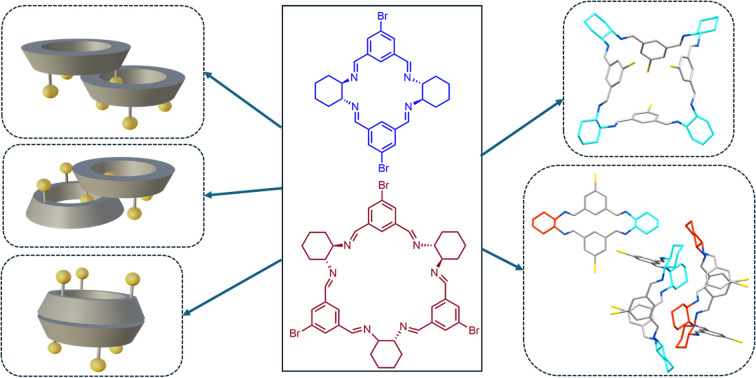

## Introduction

Solid state materials formed from discrete organic molecules with an internal void, such as macrocycles and cages, offer unique advantages and are increasingly being explored for challenging practical applications such as D_2_/H_2_ separation.^1^ Many such structures are formed using reversible chemistries, exploiting dynamic covalent chemistry (DCC) to form the thermodynamically stable product or, when multiple products with similar stabilisation energies are possible, dynamic combinatorial libraries (DCL).^2–8^ In an example of the former, a thermodynamically stable imine cage, CC3, has been used to form crystalline materials with applications in the solid state.^9–12^ The formation of multiple products is not necessarily a disadvantage in this context: ‘scrambled’ cage libraries, formed using more than two precursors, can have enhanced properties compared to the parent cages. Dynamic scrambling of two distinct amines with triformylbenzene promoted increased solubility allowing for the formation of porous liquids^13^ and increased porosity in the amorphous solid material.^14^ Dynamic scrambling of two distinct aldehyde units with cyclohexane-1,2-diamine (CHDA) was also used to form non-symmetrical cages with tuneable internal cavity size.^15^

Likewise, trianglimines and isotrianglimines, both established classes of imine macrocycles, have recently shown promise as solid-state materials.^16–18^ These Schiff-base macrocycles are formed by the condensation of *R*,*R*-CHDA or *S*,*S*-CHDA with 1,4- or 1,3-dialdehydes respectively.^19,20^ Incorporating terephthalaldehyde (1,4-dialdehyde), unsubstituted trianglimine can selectively adsorb ethyl acetate from azeotropic mixtures with ethanol.^21^ A heterochiral co-pairing strategy can be used to form a porous racemic crystal of the [3 + 3] unsubstituted isotrianglimine, formed using isophthalaldehyde (1,3-dialdehyde).^22^ Incorporation of additional functionality has resulted in substituted trianglimine macrocycles that can selectively separate industrially important molecular species.^23–27^ The reduced form of trianglimines, trianglamines, have also been used as functional materials.^28–31^

Macrocycles may have advantages over their cage counterparts, such as scope for flexibility and responsive behaviour, as well as differing pore topologies, void sizes, packing behaviour, and selectivities for substrates. However, compared to organic cage structures, where a wide range of geometries and resultant polymorphs have been reported,^32–39^ there are far fewer examples of imine macrocycle-based solid materials. This is arguably due to the relatively poor stability of imine macrocycles under crystallisation conditions, *i.e.*, in solution and during nucleation and growth of crystals.

**Fig. 1:**
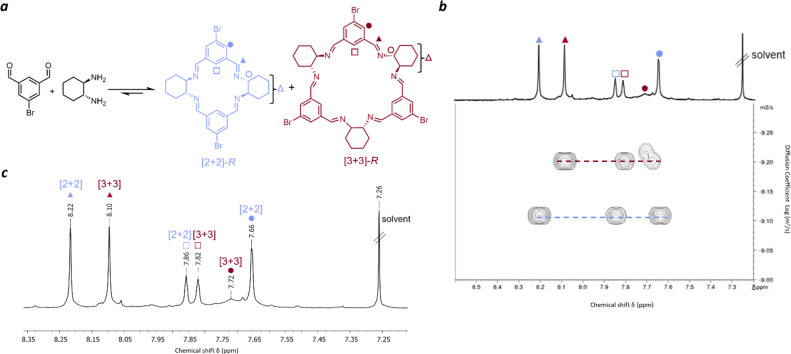
Experimental characterisation of [2 + 2] and [3 + 3] bromoisotrianglimine. (a) Reaction scheme showing the formation of [2 + 2] and [3 + 3] macrocycles from 5-bromoisophthaldehyde and *R*,*R*-CHDA; (b) pseudo 2D DOSY spectrum of the equilibrium mixture with assignment of the species present based on their diffusion coefficient; (c) ^1^H NMR (CDCl_3_) spectrum of equilibrium mixture – aromatic region.

Indeed, isotrianglimines, formed from 1,3-dialdehydes, were until recently considered less suitable for solid state studies than their trianglimine counterparts due to their dynamic behaviour in solution: the [3 + 3] macrocycle has been reported to reversibly constrict to the smaller [2 + 2] macrocycle (Fig. 1).^40,41^ Most literature examples of isotrianglimines focus on cases where recrystallisation conditions allow the isolation of the [3 + 3] species, with analytical data of the as-made reaction mixture rarely reported. Larger [4 + 4] isotrianglimines can be made, but are even rarer, with only one reported to date.^42^ However, the dynamic nature of isotrianglimines opens the possibility of using templating strategies to form new structures based on reversible bonds, resulting in new crystalline materials with differing properties, *e.g.*, new pore topologies with enhanced selectivity for a given substrate mixture, as demonstrated for organic cages.^43^

One example of an isotrianglimine system whose different sized macrocyclic products have been studied is the condensation of 2,6-diformylpyridine with either enantiopure- or (±)-CHDA.^41^ With (±)-CHDA the reaction gives the [4 + 4] and [2 + 2] macrocyclic products, which can be isolated from each other;^42^ the [3 + 3] macrocycle can be obtained by using enantiopure CHDA and recrystallising from the mixture of macrocycles,^41^ or obtained exclusively by templating with Cd(ii).^44^ In the presence of a lanthanide metal ion template, with either amine, [2 + 2]-lanthanide complexes are formed, and these have been studied extensively.^45–51^ Additionally, the isolated *meso*-[2 + 2], incorporating both *R*,*R*- and *S*,*S*-cyclohexyl moieties, can be reacted with excess CdCl_2_ to afford rearrangement into a [6 + 6]-cadmium complex.^52,53^ As such, 2,6-diformylpyridine is a simple building block that can be exploited with a degree of control to create a complex dynamic combinatorial library or to isolate specific species.^54^ However, this dynamic behaviour has been much less explored in other isotrianglimine systems or for solid state materials. As isotrianglimines are readily derivatised with a wide range of chemical functionalities, greater control of their synthesis and crystallisation would enable controllable engineering of diverse material types with variable porosity and chemical selectivity. In this vein, Kwit and co-workers recently explored how chiral isotrianglimines pack in the solid state, studying single crystal structures of four different C-5 substituted isotrianglimines (R = OMe, NO_2_, *t*-Bu, Br), examining if crystallisation from different solvents would induce polymorphism and new packing motifs. However, this study focused exclusively on the [3 + 3] derivatives.^55^

Here, we focus on isotrianglimines formed using 5-bromoisotrianglimine to explore the structural diversity possible from this simple building block. First, diffusion NMR was used to study the reaction mixture and to enable assignments for the different macrocyclic species present in solution. With this in hand, VT-NMR experiments were carried out to probe both conversion between different macrocyclic species and the conformational behaviour of the macrocycles in solution. Computational studies were carried out to elucidate the relative stabilities of the [2 + 2] and [3 + 3] macrocyclic compounds and evaluate whether either could be described as the kinetic or thermodynamic product. Then, crystallisation experiments were performed, demonstrating how different crystallisation solvents can not only alter the packing of [3 + 3] dimers, here reporting a new tail-to-head arrangement not seen for isotrianglimines, but can also alter the equilibrium by templating the formation of larger macrocycles, in this case a [4 + 4] crystal structure templated by 1,4-dioxane. Finally, we report how having opposing enantiomers present also influences the outcomes of crystallisation, reporting a *meso*-[2 + 2] crystal structure.

## Results and discussion

### Solution phase

Before studying isotrianglimines in the solid state it is important to understand the solution phase behaviour. Therefore, the synthesis of the brominated isotrianglimine was first explored. The C-5 brominated isotrianglimine has been previously synthesised and reported to form the [3 + 3] macrocycle in yields of 69% & 97%;^55,56^ the presence or absence of [2 + 2] macrocycle in reaction mixtures is rarely discussed. In our hands, reacting 5-bromoisophthaladehyde with *RR*-CHDA or *SS*-CHDA in chloroform at an overall concentration of 0.1 M overnight produced a mixture of the [2 + 2] and [3 + 3] macrocycles (Fig. 1a). As only ^1^H NMR spectra for the isolated [3 + 3] macrocycle was reported,^55,56^ both 2D and diffusion NMR was carried out to complete the assignment for the mixture of macrocycles and to enable reaction monitoring over time.

Diffusion-ordered spectroscopy provides information about the rate of diffusion of different species in a solution.^57,58^ In this case, as the diffusion coefficient is related to the solvodynamic radii, it could be used to resolve the NMR signals of distinct species in solution based on their size (Fig. 1b) and to confirm which proton signals belonged to each macrocycle. Peaks at 8.22 ppm, 7.86 ppm and 7.66 ppm were found to have an average diffusion coefficient of 6.61 × 10^−10^ m^2^ s^−1^ while those at 8.10 ppm, 7.82 ppm and 7.72 ppm were found to have an average diffusion coefficient of 5.48 × 10^−10^ m^2^ s^−1^. The larger diffusion coefficient (6.61 × 10^−10^ m^2^ s^−1^) corresponds to a smaller solvodynamic radius, indicating that these peaks correspond to the [2 + 2] macrocycle, while the others correspond to the [3 + 3] species. With COSY and NOESY NMR data, full assignments of the [2 + 2] and [3 + 3] macrocycles could therefore be made (Fig. 1c and S6–S11†). Mass spectrometry of the mixture also confirmed the presence of both [3 + 3] and [2 + 2] macrocycles, and interestingly, also a [4 + 4] species, with corresponding masses of 583.08, 875.12, and 1165.16 respectively (Fig. S3†).

Previously, it has been reported that prolonged refluxing of structurally related, methylated isotrianglimines can result in contraction of the [3 + 3] macrocycle into the smaller [2 + 2] species.^41^ However, with the brominated isotrianglimine, any attempts to shift the equilibrium in solution, such as by changing the reaction solvent (chloroform, DCM, toluene, THF) and/or the reaction temperature (room temperature, refluxing) resulted in the presence of both [2 + 2] and [3 + 3] macrocycles in similar proportions, as measured by the relative intensities of their corresponding imine peaks in the resulting ^1^H NMR spectra (Fig. S13 and S14†). The [3 + 3] macrocycle can be isolated by recrystallisation in hot ethyl acetate (Fig. S21†), however, when left in solution it re-equilibrates to a mixture of the [2 + 2] and [3 + 3] macrocycles (Fig. S26†).

Gawroński *et al.* employed molecular modelling to investigate the formation of the triangular macrocycles, and reported that the products of the reaction between isophthalaldehyde and *R*,*R*-CHDA were governed by conformational constraints, *i.e.*, the geometry of the 1,3-dialdehyde results in a less stable [3 + 3]-isotrianglimine compared to the equivalent [3 + 3]-trianglimine formed from 1,4-dialdehyde.^19^ It has previously been suggested that for isotrianglimines the [3 + 3] macrocycle is the kinetic product, while the [2 + 2] is the thermodynamic product, with most studies focusing on the unsubstituted isotrianglimine. Nour *et al.* used real-time electrospray ionization time-of-flight mass spectrometry to monitor the formation and interconversion of [2 + 2] and [3 + 3] unsubstituted isotrianglimine at 1.2 M in DCM over 6 hours.^40^ Eleven different reaction intermediates were detected in this study, which included mono-, penta-, and longer-chain polyimines. The intensity of the mass peak corresponding to the [3 + 3] macrocycle reached a maximum value at 10 hours, after which there was an increase of the [2 + 2] macrocycle at the expense of the [3 + 3] macrocycle supporting the conclusion that the [3 + 3] macrocycles were the kinetic product, and the [2 + 2] macrocycle the thermodynamically stable product. However, it is possible that sample preparation and ionization in mass spectrometry can perturb the dynamic equilibrium.^59^

**Fig. 2:**
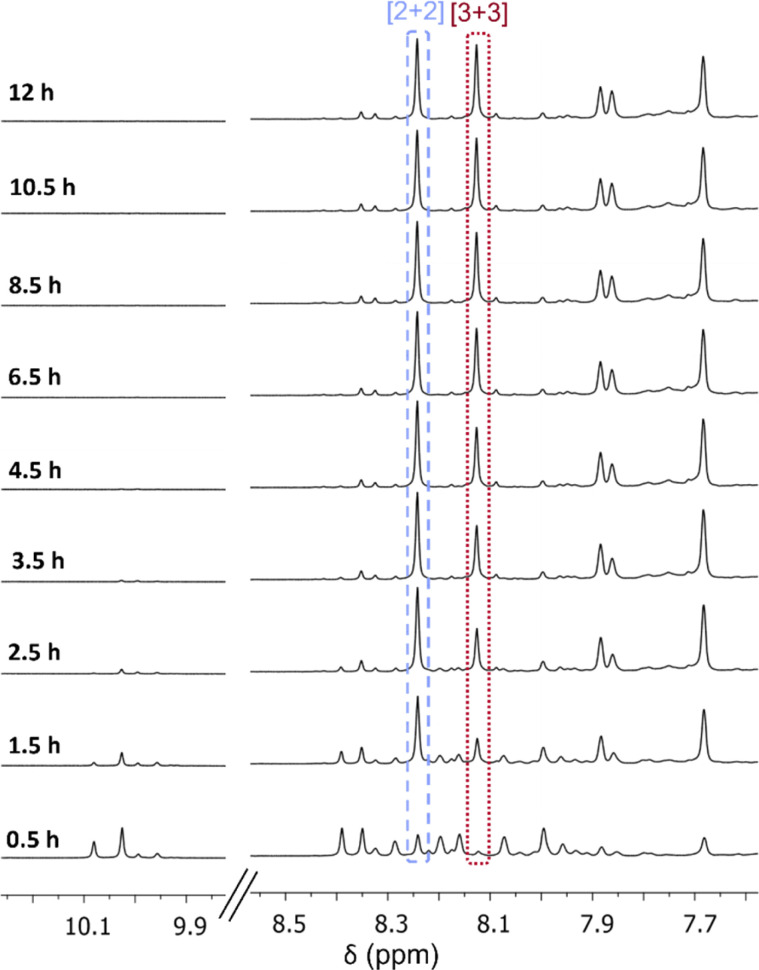
^1^H NMR (CDCl_3_) following the formation of [2 + 2] and [3 + 3] macrocycles over 12 hours.

Therefore, to observe if the [3 + 3] macrocycle contracts into the [2 + 2] macrocycle in solution over time when C-5 functionality is added to the isophthalaldehyde, the formation of the brominated isotrianglimines in solution were monitored over time by ^1^H NMR spectroscopy (Fig. 2). ^1^H NMR spectra were recorded every 30 minutes up to 12 hours. After the initial 30 minutes, multiple aldehyde and aromatic peaks were present, indicating the formation of intermediates. By 1.5 hours, the imine peaks of both macrocycles could clearly be seen; the ratio of [2 + 2] : [3 + 3] present was 1 : 0.23. After 8 hours the ratio of [2 + 2] : [3 + 3] was 1 : 0.6 and this remained relatively stable; the ratio of [2 + 2] : [3 + 3] at 12 hours was 1 : 0.65. This is in contrast with observations for the unsubstituted isotrianglimine where it was reported that the [3 + 3] initially forms in larger quantities and converts over time to the [2 + 2].^40^

**Fig. 3:**
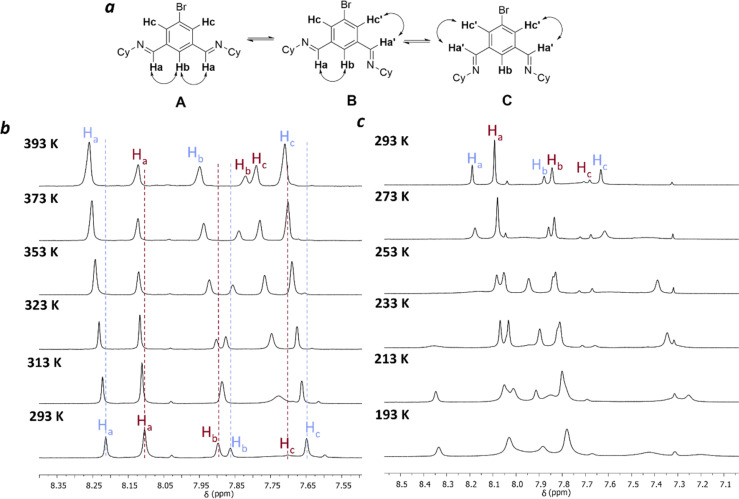
VT NMR Studies of the brominated mixture of macrocycles: (a) possible conformations for [3 + 3] macrocycle; (b) hot VT NMR, 293–393 K (tetrachloroethane-d_2_); (c) cold VT NMR, 293–193 K (dichloromethane-d_2_).

Next, variable temperature ^1^H NMR studies were carried out to investigate the dynamic interconversion between the [3 + 3] and [2 + 2] macrocycles (Fig. 3 and S15–S18†). Ideally, this should be carried out in the same solvent used throughout the study, but due to the limited solubility of the macrocycles, the mixture of equilibrated macrocycles was dissolved in tetrachloroethane-d_2_ for higher temperature ranges (298–393 K) and in dichloromethane-d_2_ for lower temperature ranges (298–193 K).

Unfortunately, the complexity of the spectra at reduced temperatures precluded unambiguous assignment (Fig. 3c). At elevated temperatures, the [2 + 2] macrocycle is favoured with respect to the [3 + 3] macrocycle (Fig. 3b). A van't Hoff analysis was carried out to extract the corresponding enthalpy and entropy values for the equilibrium between 3[2 + 2] and 2[3 + 3] macrocycles (ESI Section 1.3†). Between 293–343 K a non-linear relationship was observed (Fig. S19†), implying that the assumption that only two macrocyclic species are involved in this process at all temperatures studied is not satisfied. The implication that other species are involved over these temperatures is supported by the observations that there are significant shifts in the peaks corresponding both to the macrocyclic species, and to peaks assigned to water and residual DCM/chloroform in the spectrum, indicating potential binding interactions at these temperatures. Conformational changes for the [3 + 3] macrocycle are also possible, for example rotation around the imine bond (Fig. 3a), similar to the observed behaviour in the unsubstituted isotrianglimine,^19^ as evidenced by the broad peak for H_c_ at 7.1 ppm that is hidden at 293 K but sharpened at temperatures above 313 K (Fig. 3b).

A van't Hoff analysis was therefore carried out for those temperatures where a linear relationship was observed (353–393 K, Fig. S20†). From these points, the corresponding enthalpy and entropy for the equilibrium of 3[2 + 2] ↔ 2[3 + 3] in TCE-d_2_ are Δ*H*_0_ = – 21.5 kJ mol^−1^ and Δ*S*_0_ = −39.0 J K^−1^ mol^−1^ respectively.

**Fig. 4:**
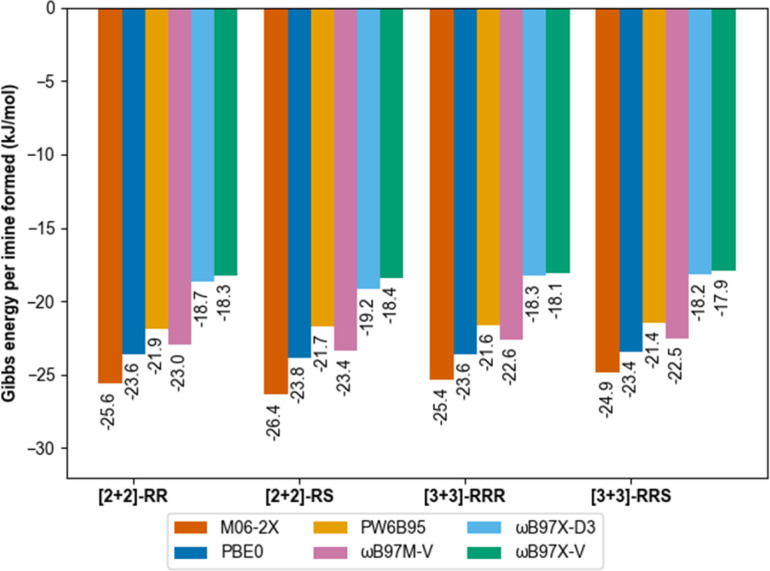
Gibbs energy of formation for the different macrocycles investigated in this study obtained with different DFT functional and def2-QZVP basis set in implicit chloroform (SMD model). Solvation model was crucial to identify low-energy structures consistent with experimental data. Entropy contributions were calculated according to the QRRHO following thermochemistry calculations at B97-3c level of theory.

Gas phase molecular modelling was also used to explore the relative energies of the macrocycles. Conformational sampling using the GFN2-xTB^60,61^ semi-empirical method within the CREST^62,63^ package identified only five low-lying energy conformations for the [2 + 2] macrocycle and 16 low-lying conformations for the [3 + 3] macrocycle. Lowest-energy conformations were identified by further geometry optimisation and energy ranking at the low-cost B97-3c^64^ DFT functional. Formation energies of those structures ([2 + 2]-*R*, *meso*-[2 + 2], [3 + 3]-*R*, [3 + 3]-*RRS*, Fig. S58 and S59†) were then calculated using six different modern dispersion-corrected^65–67^ DFT functionals (M06-2X-D3(0),^68^ PW6B95-D3(BJ),^69^ ωB97X-D3, ωB97X-V,^70^ ωB97M-V^71^ and PBE0-D3(BJ))^72^ with a large def2-QZVP basis set.^73,74^ The results were consistent (within 1 kJ mol^−1^) and showed no thermodynamic preference per imine bond between the structures (Fig. 4).

DFT calculations were performed using Orca 5.0.1 (ref. 75) using the universal solvent model based on density^76^ for implicit treatment of chloroform as the reaction solvent. We previously used a similar approach to predict the outcomes of much more rigid covalent cages made from mixed sub-components, in which case the formation energies corresponded directly to the observed reaction outcomes.^15^ In the current study, however, molecular modelling indicates there is a fine balance between the formation of the [2 + 2] and [3 + 3] macrocycles, which supports the experimental observation that both species are formed in solution under all conditions screened.

With full assignment and solution phase understanding in hand, we next investigated the crystallisation process for both homochiral and heterochiral macrocycles.

### Solid state

#### Homochiral

**Fig. 5:**
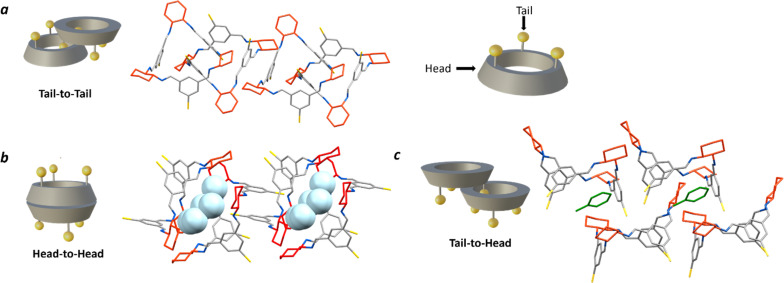
Crystal packing of [3 + 3]-*R* in the three possible dimer arrangements, hydrogen atoms omitted in all for clarity. (a) Tail-to-tail, THF/MeOH (solvents omitted); (b) head-to-head (capsule), THF/heptane with H_2_O encapsulated (light blue); (c) tail-to-tail; toluene (green)/MeCN. Carbon – grey, nitrogen – blue, bromine – yellow, *R*,*R*-cyclohexyl ring – red.

First, the crystallisation behaviour of the homochiral macrocycles were investigated by using the equilibrated mixture of the [2 + 2] and [3 + 3] macrocycles and screening a range of solvents (THF, DCM, EtOAc; antisolvents: MeOH, heptane, toluene, dioxane, hexane, acetonitrile). The majority of these crystallisation conditions selectively crystallised the [3 + 3] macrocycle. Macrocycles of [3 + 3] isotrianglimines with functionalisation at the C-5 position have the ability to self-assemble in several different possible arrangements: (i) tail-to-tail dimer; (ii) head to-head-dimer; and (iii) head-to-tail dimer (Fig. 5).^55,56^ Previous structurally characterised examples are known to associate as both the tail-to-tail and head-to-head capsule forms, the former being more prevalent, even in the presence of hydrogen-bond directing hydroxy groups at the C-3 position in known examples.^77^ This was rationalised by Kwit and co-workers in a study of the assembly of C-5 functionalised [3 + 3] isotrianglimines in the gas phase using ion mobility mass spectrometry, DOSY-NMR, optical rotation measurements, and semiempirical theoretical calculations, concluding it is energetically favourable for C-5 brominated [3 + 3] macrocycles to pack as a tail-to-tail dimer rather than a head-to-head dimer.^56^

More recently, Kwit and co-workers continued their investigation by exploring dimer arrangements in the solid state in a small library of C-5 substituted isotrianglimines, reporting two non-isomorphous crystal forms of the brominated [3 + 3] species.^55^ They reported that when brominated isotrianglimine crystals are grown from anhydrous DCM/MeCN the [3 + 3] macrocycles assemble as solvent-free discrete tail-to-tail dimers (Fig. 5a), but when water was present in the system a very different structure was adopted, featuring a tail-to-tail association wherein an head-to-head capsule is formed between adjacent dimeric pairs containing inclusion solvent (Fig. 5b). In our studies, crystallisation by vapour diffusion of MeOH into THF resulted in crystals isomorphous with that of the discrete tail-to-tail structure reported by Kwit and co-workers (Fig. 5a) yet possessing a pronounced 0.6 Å increase in length of the crystallographic *b* axis. Both solvated and desolvated structures were obtained (Fig. S57†).

The [3 + 3]-*R* head-to-head form reported by Kwit features intermolecular complementary capsule association between neighbouring tail-to-tail dimers, within which resides water from the crystallisation solvent and a small amount of non-encapsulated MeCN (Fig. 5b).^55^ In our screen, isostructural crystals to the reported structure were formed from wet THF/heptane, which contained an almost identical amount of inclusion water. Kwit and co-workers speculated that this head-to-head motif would only appear in isotrianglimine crystals when solvent molecules containing functional groups capable of forming hydrogen bonds are involved.^55^ The hydrogen bonding network provided by the encapsulated water molecules appears to be key in driving this secondary head-to-head capsule arrangement, wherein a pillared type structure is constructed as an alternating dimer-capsule-dimer arrangement which propagates parallel to the crystallographic *a* axis.

Interestingly, when toluene and xylenes were employed as the anti-solvents, an unreported tail-to-head motif was observed, with the aromatic solvent found to be situated between the two macrocycles (Fig. 5c). Solid state structures of the unsubstituted parent homochiral isotrianglimine incorporating aromatic solvents have been reported, but of the three presented, two form a one-dimensional porous columnar structure consisting of isolated, non-interacting [3 + 3] macrocycles wherein the aromatic guest is located within the channels, and the third adopts the more routinely observed tail-to-tail dimeric association despite the absence of functionalisation at the benzene C-5 positions.^55^

**Fig. 6:**
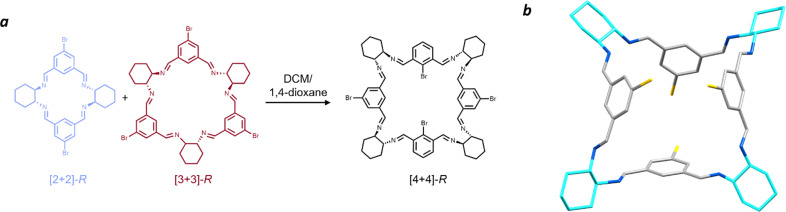
(a) Schematic showing the crystallisation conditions for formation of the [4 + 4] brominated isotrianglimine (b) Single crystal structure of homochiral [4 + 4]-*S* bromoisotrianglimine. Solvent omitted for clarity. Carbon – grey, nitrogen – blue, bromine – yellow, *SS*-cyclohexyl rings – cyan.

Surprisingly, when 1,4-dioxane was used as the antisolvent, a new macrocycle was isolated: a [4 + 4] species (Fig. 6). During our synthetic and spectroscopic studies, the [2 + 2] and [3 + 3] forms were the major products, with the [4 + 4] species only detected through mass spectrometry analysis and not isolated in the bulk material. There are reports of metal complexes with [4 + 4] Schiff base macrocycles,^52,78–83^ but only limited examples for trianglimine^84^ or isotrianglimine^42^ derivatives, the latter of which was characterised crystallographically, however as a *meso*-rather than homochiral structure.

Vapour diffusion of 1,4-dioxane into a solution of the equilibrated [2 + 2] and [3 + 3] macrocyclic mixture in DCM yielded large, yellow prismatic crystals, in contrast with the colourless appearance of all other forms of bromoisotrianglimine crystals presented herein. These prismatic crystals were sensitive to rapid desolvation and loss of crystallinity, suggestive of potential porosity. Numerous attempts were made to obtain publishable quality diffraction data on samples of both the homochiral *R*- and *S*-enantiomers with the [4 + 4]-*S* macrocycle (Fig. 6) form yielding slightly larger crystals resulting in the strongest diffraction. The macrocyclic form can be considered pseudo-cage-like in arrangement, crystallising as a poly-1,4-dioxane solvate in which there are four chemically unique solvent environments (Fig. S30†). Three of the 1,4-dioxane molecules are external to the macrocycles, with the fourth encapsulated within the central cavity of the [4 + 4]-*S* macrocycles and disordered only by symmetry, strongly indicative of possible solvent templation driving the formation of this larger macrocyclic structure.

1,4-Dioxane has been shown to direct the crystal packing for imine cage CC2 away from its lowest energy polymorphs to form isostructural 3-dimensional diamondoid pore channels.^85^ In that study, several internal cavity void calculations were reported on these structures, which ranged from 116–125 Å^3^. By removing the modelled internal 1,4-dioxane from our [4 + 4]-*S* structure, a PLATON Squeeze routine at the 1.2 Å level calculated an internal void space *ca.* 13–22% larger at 141 Å^3^. This is consistent with the volume of 1,4-dioxane as inferred from crystallographic data alone, which typically ranged from 111 to 121 Å^3^ depending on the conditions under which the crystal was measured.^86,87^ To the best of our knowledge, this is the first single crystal structure of a homochiral [4 + 4] isotrianglimine, obtained through solvent templation. Gregoliński *et al.* reported the first example of a Schiff base [4 + 4] crystal structure from 2,6-diformylpyridine, but in its *meso*-[4 + 4] form.^42^ The only other example of a similar solvent templation towards a [4 + 4] imine macrocycle has been seen in a trianglimine derived from 9,10-diphenylanthracene-based dialdehyde and *R*,*R*-CHDA where *p*-xylene templates the formation; while no crystal structure of the imine was obtained, they were able to reduce it to the amine counterpart for further study.^84^ As expected, when the [4 + 4]-bromoisotrianglimine crystal is exposed to air allowing evaporation of dioxane, the crystal rapidly decomposes. We were able to obtain a ^1^H NMR spectrum (Fig. S27†), however the [4 + 4] macrocycle rapidly converts back to a mixture of the [2 + 2] and [3 + 3] species in solution limiting any further structural investigation (Fig. S28†).

Molecular modelling was performed as for the smaller macrocycles. Unfortunately, this did not identify any conformation with persistent cavity for the [4 + 4] macrocycle: all lowest-energy conformations were globular “collapsed” structures (Fig. S60†). Only when a molecule of dioxane was manually placed in the starting conformations and the ‘non-covalent interactions’ mode was used in CREST, were we able to bias the search to identify a chemically reasonable dioxane [4 + 4] superstructure (Fig. S61†).

Although the [4 + 4] macrocycle was not stable to desolvation, and the tail-to-head arrangement did not induce porosity into the homochiral [3 + 3] macrocycle, both strategies could yield porous materials for other functionalised isotrianglimines. High-throughput screening of crystallisation conditions could be a useful tool for further exploring the effect of crystallisation solvents on the arrangement of isotrianglimines in the solid state.^88^ Such screening could also exploit another tool to direct the crystal packing in molecular materials: chiral recognition.^89–93^ Cooper and co-workers invoked the possibility of chiral recognition to achieve the capsule head-to-head motif on the unsubstituted isotrianglimine.^21^ Therefore, we explored the use of heterochiral co-crystallisation of bromo-isotrianglimine to investigate the effect on both the polymorphs obtained and the macrocycles present in the structure.

#### Heterochiral

**Fig. 7:**
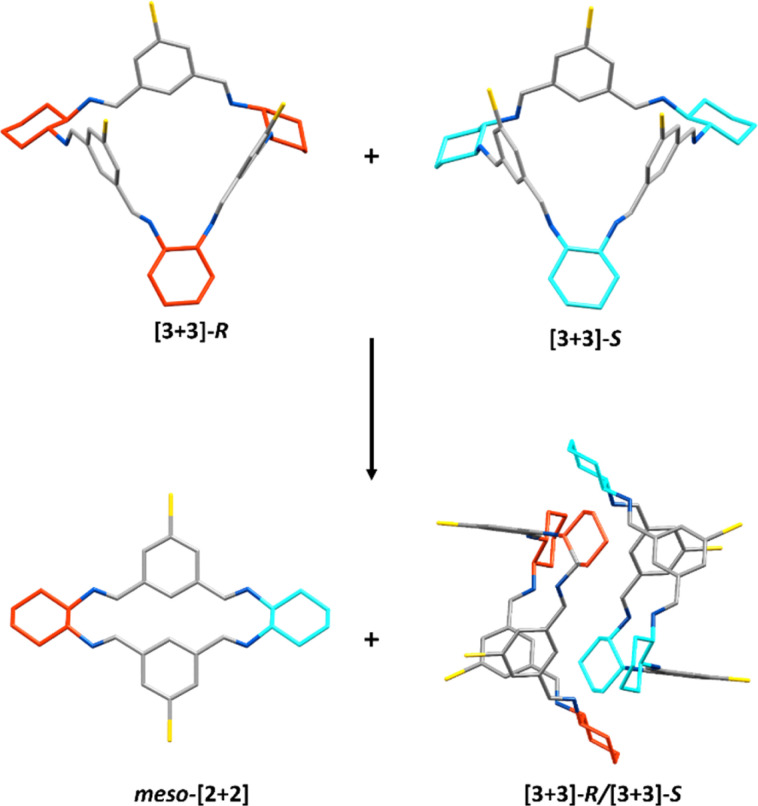
Crystallisation of equimolar [3 + 3]-*R* and [3 + 3]-*S* can lead to both *meso*-[2 + 2] and the co-crystal [3 + 3]-*R*/[3 + 3]-*S* which arranges in the head-to-head ‘capsule’ dimer arrangement. Carbon – grey, nitrogen – blue, bromine – yellow, *S*,*S*-cyclohexyl rings – cyan, *R*,*R*-cyclohexyl rings – red.

For heterochiral investigations, the equilibrated mixture of the [2 + 2] and [3 + 3] macrocycles was also synthesised from *S*,*S*-CHDA. For investigation of small-scale co-crystallisation equimolar amounts of the *R*,*R*-and *S*,*S*-CHDA-derived equilibrated mixtures were subsequently dissolved in ethyl acetate, and hexane used as an antisolvent. This resulted in the formation of the racemic co-crystal [3 + 3]-*R*/[3 + 3]-*S* of the brominated isotrianglimine (Fig. 7). The opposite chiralities of the [3 + 3] macrocycle complement each other, enabling the formation of a capsule motif. In this motif, there is alternating tail-to-tail association or dimeric pairs of the same handedness; the co-crystals are comprised of alternating [3 + 3]-*R* tail-to-tail and [3 + 3]-*S* tail-to-tail motifs. Given the presence of the bulky bromine atoms, the cocrystallised [3 + 3]-*R*/[3 + 3]-*S* brominated isotrianglimine structure was not expected to be porous which was confirmed by gas sorption measurements (Fig. S47†).

However, the [3 + 3]-*R*/[3 + 3]-*S* co-crystal was not the only material that was formed during these heterochiral crystallisation studies. Large scale crystalline material could be obtained by recrystallising equimolar amounts of the *R*,*R*- and *S*,*S*-CHDA-derived reaction mixtures in hot ethyl acetate; on cooling the co-crystallised [3 + 3]-*R*/[3 + 3]-*S* precipitated out of solution. However, upon leaving the cooled reaction mixture to stand over time, the amount of [3 + 3]-*R*/[3 + 3]-*S* that precipitated decreased and a new species was found to co-precipitate: the *meso*-[2 + 2] macrocycle (Fig. 7). Eventually the latter precipitates exclusively, allowing collection of the isolated *meso*-[2 + 2] species (Fig. S37†). ^1^H NMR analysis of the remaining ethyl acetate supernatant showed a soluble imine species with different proton signals compared to either the *meso*-[2 + 2] or [3 + 3]-*R*/[3 + 3]-*S* species, albeit with large quantities of EtOAc present in the solution (Fig. S38 and S39†). When this sample was concentrated to reduce EtOAc content, peak shifts were observed, resulting in a complex spectrum (Fig. S40†). As no aldehyde peaks were observed, both spectra are indicative of mixtures of multiple imine species. The dynamic changes observed on perturbation of the solution made any analysis of the species present challenging. Further investigation would be required to confirm the identity of such species, for example by freezing of the dynamic behaviour *via* reduction of the mixture; however, it is possible that the composition of the mixture would change further during reduction.

Thus, to explore this further, we investigated the solution phase behaviour of each heterochiral species by dissolving the obtained pure samples of *meso*-[2 + 2] and [3 + 3]-*R*/[3 + 3]-*S* and taking ^1^H NMR spectra at intervals. When either the *meso*-[2 + 2] macrocycle or the co-crystallised [3 + 3]-*R*/[3 + 3]-*S* were separately left in CDCl_3_ for an extended period of time (3 months), they both equilibrated to the same mixture of products as analysed by ^1^H NMR spectroscopy, reaching equilibrium by 1 month (Fig. S42 and S43†). In the ^1^H NMR spectrum of this mixture, peaks matching those of chiral [3 + 3], *meso*-[2 + 2], and chiral [2 + 2] macrocycles were identified, alongside peaks that did not overlap with any of the species identified thus far (Fig. S44†). The unidentified peaks also did not match those seen in the ethyl acetate supernatant (Fig. S4†), which we have tentatively assigned as kinetic products due to the observed changes in their composition with any perturbation of the system. We were unable to isolate or confirm assignment of these species; it is possible that they are macrocyclic species containing both *R*,*R*- and *S*,*S*-CHDA units, such as [3 + 3]-*RRS* (Fig. 4 and S59†), as seen for organic cage CC3-*RS* formed from racemic cyclohexane-1,2-diamine.^94^

In contrast to the homochiral crystallisation studies in the same solvent systems, introducing both chiralities of the brominated isotrianglimine in DCM/1,4-dioxane gave single crystals of both the [3 + 3]-*R*/[3 + 3]-*S* and *meso*-[2 + 2] species, and did not yield any single crystals of the [4 + 4] macrocycle. Therefore, it can be assumed that when both enantiomers of CHDA are present it is more favourable for the [2 + 2] and [3 + 3] macrocyclic products to crystallise than the 1,4-dioxane templated [4 + 4] species.

Having observed the formation and crystallisation of *meso*-[2 + 2] it is worth noting that during this work multiple crystallisation conditions were trialled, but none afforded the homochiral [2 + 2]-*R* that was observed in solution. The [2 + 2]-*R* macrocycle may have significant strain or may not pack efficiently in the solid state, as can be predicted from the modelled structure (Fig. S58†), thus it may have a relatively high stabilisation energy and be unlikely to form under crystallisation conditions.

## Conclusions

Isotrianglimines as dynamic macrocycles are a versatile platform for future exploration, and whose crystallisation behaviour and dynamic equilibrium can be readily influenced by solvents. Here, we have reported multiple new motifs and structures from the simple reaction of bromoisophthalaldehyde and cyclohexane-1,2-diamine, including: full characterisation of the chirally pure [2 + 2] macrocycle in solution, as part of a mixture of *R*,*R*-[2 + 2] and *R*,*R*-[3 + 3] species; a new tail-to-head packing motif from aromatic solvent inclusion; a 1,4-dioxane templated [4 + 4] macrocycle; a racemic co-crystal of [3 + 3]-*R*/[3 + 3]-*S*; and the *meso*-[2 + 2] species.

In this case, porous materials were not obtained, however this work has uncovered multiple strategies to influence the packing of isotrianglimine macrocycles. It is likely that including different functional groups at C-5 and exploiting these strategies will afford materials with interesting properties, especially in combination with high-throughput screening approaches. We anticipate that re-examination of existing isotrianglimines using these approaches will uncover further opportunities to tune the solid-state structures and applications of these materials.

## Data availability

Structures of identified conformations and further optimised geometries used for single point DFT calculations, together with a summary of the computational results can be found on GitHub: https://github.com/fiszczyp/aic-trianglimine/. CCDC 2312774–2312783 contain the supplementary crystallographic data for this paper. These data can be obtained free of charge from the Cambridge Crystallographic Data Centre and are also uploaded as ESI.†

## Author contributions

Conceptualisation and funding acquisition was carried out by AGS. Supervision carried out by AGS and RLG. Experiments and methodology were carried out by AMS. AMS ran crystallisation experiments, structural data was collected and solved by LKC. FTS was responsible for modelling calculations. KL collected VT-NMR experiments. BDE and RLG were responsible for DOSY experiments and data processing. All authors contributed to experimental design, analytical measurement, and interpretation of results. AMS produced the first draft and all authors contributed to the final manuscript.

## Conflicts of interest

There are no conflicts to declare.

## Supplementary Material

SC-OLF-D4SC04207G-s001

SC-OLF-D4SC04207G-s002
