# Supplementary material for: Dynamic and solid-state behaviour of bromoisotrianglimine
Source: Chem Sci. 2024 Aug 13;15(35):14254–63. doi: 10.1039/d4sc04207g (PMC11320023; doi:10.1039/d4sc04207g)
Supplement: SC-015-D4SC04207G-s001 [file SC-015-D4SC04207G-s001.pdf]

Supplementary Information (SI) for Chemical Science.
This journal is © The Royal Society of Chemistry 2024

Supplementary Information

Dynamic and solid-state behaviour of bromoisotrianglimine

Abbie M. Scholes,[a] Laurence J. Kershaw Cook,[a] Filip T. Szczypiński,[a] Konstantin Luzyanin,[a] Benjamin D. Egleston,[b] Rebecca L. Greenaway[b] and Anna G. Slater* [a]

***** E-mail: anna.slater@liverpool.ac.uk

[a]Department of Chemistry and Materials Innovation Factory, University of Liverpool, Liverpool, UK.

[b]Department of Chemistry, Molecular Sciences Research Hub Imperial College London White City Campus, 82 Wood Lane, W12 0BZ London, UK

Contents

1. Experimental 3
 1.1 Experimental details 3
 1.2 Synthesis 5
  1.2.1 Materials 5
  1.2.2 Equilibrium mixture of [2+2] and [3+3] brominated isotrianglimine 5
  1.2.3 Characterisation data 6
 1.3 Variable Temperature ^{1}H NMR 14
 1.3.1 Van't Hoff analysis 18
 1.4 Recrystallisation to [3+3]-*R* 19
 1.5 1,4-Dioxane solvent templation of [4+4] 23
 1.6 [3+3]-*R/*[3+3]-*S* and *meso*-[2 + 2] 27
 1.7 Diffusion NMR for bromoisotrianglimine 38
  1.7.1 Decay Curves 39
2. Single Crystal data 43
 2.1 Crystallographic tables 44
 2.2 Additional Information for single crystal data 46
3 Molecular Modelling 47
4 References 51

1. Experimental

1.1 Experimental details

Nuclear Magnetic Resonance (NMR) Spectroscopy

Solution ^{1}H and ^{13}C NMR spectra were recorded at 400.13 MHz and 100.6 MHz respectively using a Bruker Avance III HD 400 NMR spectrometer. Chemical shifts are reported in ppm (δ) with reference to internal residual protonated species of the deuterated solvent used for ^{1}H and ^{13}C analysis.

High-Resolution Mass Spectrometry (HRMS)

HRMS was carried out using an Agilent Technologies 6200 series TOF/6500 series Q-TOF Dual ESI mass spectrometer (capillary voltage 4000 V, fragmentor 225 V) in positive-ion detection mode.

Single Crystal X-ray Diffraction (SCXRD)

Apart from *in-situ* desolvated [3+3]-*R*, all crystals were subjected to lab-source diffraction experiments and directly extracted from the relevant solvent media and immersed in viscous Fomblin perfluorinated oil before mounting on either 20 or 50 µm MiTeGen dual thickness micromounts. The crystals were transferred directly into the nitrogen coldstream (100 or 150 K) controlled by an Oxford Cryosystems 700+ cryostream controller. The structure of [3+3]-*R*•(MeOH) was acquired using a Bruker D8 Venture diffractometer equipped with a photon 100 dual-CMOS chip detector and operating a Mo Kα (λ = 0.71073) microfocus X-ray source with data collection and processing conducted using Bruker APEX3 software. All other structure determinations presented were collected using a Rigaku AFC12K goniometer employing mirror monochromated Mo Kα radiation (λ = 0.71073 Å) radiation generated from a Rigaku 007HF Molybdenum rotating anode microfocus X-ray target source and using a Rigaku Hypix-6000HE Hybrid Photon Counting detector. To obtain the desolvated structure of [3+3]-*R*, a crystal was extracted from its methanolic crystallisation liquor and mounted under the cryostream at 290 K, before heating to 375 K and holding the crystal at this temperature for 30 minutes. The temperature was then cooled to 100 K where the full structure determination of the desolvated phase was confirmed. All data reduction and processing for the structures acquired using the Rigaku instrument was performed using the CrysAlisPro software package and empirical absorption corrections using spherical harmonics

were implemented in the SCALE3 ABSPACK scaling algorithm. The structures were solved using either direct or dual-space methods and refined by full matrix least-squared on F2 with SHELXL2018. The interface of the OLEX2 software suite was used to develop the structures during modelling. Unless otherwise specified, hydrogen atoms were placed in calculated positions and refined using a riding model. In the main text, van der Waals radii of atoms are taken into account on the reported distances between atoms and the solvent accessible surfaces where necessary were obtained through a PLATON Squeeze analysis are calculated with probe radius of 1.2 Å. Structural data are summarised in SI Section 2, Tables 2 & 3. Crystallographic figures were prepared using Mercury 2022.1.0.

Powder X-ray Diffraction (PXRD)

PXRD data were collected in transmission mode on samples held on thin Mylar film in stainless steel well plates on a Panalytical Empyrean MPD equipped with a high throughput screening (HTS) XYZ stage, X-ray focusing mirror, and PIXcel detector, using Cu Kα radiation.

Laboratory diffractometer based PXRD experiments on [4+4]-*S*•(1,4-dioxane) were carried out at room temperature on a Bruker D8 Advance diffractometer with a monochromated Cu source (Kα1, λ = 1.5406 Å) in Debye-Scherrer geometry. The samples were briefly ground using an agate mortar and pestle and loaded into 0.7 mm internal diameter borosilicate glass capillaries. To study the effects of desolvation with time and temperature, a 1,4-dioxane immersed sample was studied, followed by two experiments on the extracted solid material, the first of which was allowed to air dry on the bench for a period of minutes prior to loading and measurement, and the second annealed at 75°C for 18 h overnight before acquiring its powder profile.

Gas Sorption Analysis

Surface areas were measured by nitrogen adsorption and desorption at 77.3 K. Powder samples were degassed offline at 100 °C under dynamic vacuum before analysis, followed by degassing on the analysis port under vacuum, also at 100 °C. Isotherms were measured using a Micromeritics ASAP 2020 or 2420 volumetric adsorption analyser.

1.2 Synthesis

1.2.1 Materials

(1*R*,2*R*)-1,2-Cyclohexanediamine (*R,R*-CHDA) and (1*S*,2*S*)-1,2-cyclohexanediamine (*S,S*-CHDA) were purchased from Fluorochem. 5-Bromoisophthalaldehyde was purchased from Manchester Organics. All other chemicals and solvents (HPLC grade/ anhydrous) were purchased from Sigma-Aldrich or Fischer Scientific and used as received.

1.2.2 Effect of reaction conditions on the formation of [2+2] and [3+3] brominated isotrianglimine

Screening experiments were carried out to explore the impact of conditions on macrocycle formation. The temperature, solvent, amine ratio, use of inert atmosphere, dry vs. bench chloroform, and addition rate of amine were investigated, and the reaction was monitored over time (see Table 1). In all cases, a mixture of [2+2] and [3+3] macrocycles was obtained, with a relative ratio of [2+2]:[3+3] of between 1:0.60 (chloroform, reflux) and 1:1.5 (THF, RT or reflux). The experimental conditions below represent the conditions where the presence of starting material aldehyde were not observed by NMR and a relative ratio of [2+2]:[3+3] macrocycles of 1:0.7 was consistently produced. NMR results for the different solvents trialled can be seen in Figures S13 and S14.

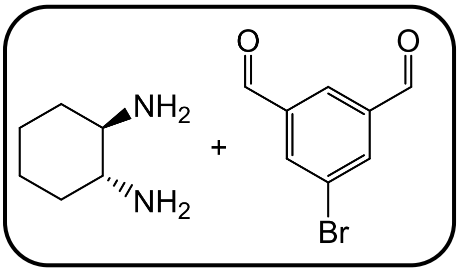

To a stirring solution of 5-bromoisophthaladehyde (1.00 g, 4.96 mmol, 1 eq) in dry chloroform (50 mL, 0.1 M) under nitrogen, a solution of *R,R*-CHDA, 0.56 g, 4.96 mmol, 1 eq) in dry chloroform (50 mL, 0.1M) under nitrogen was added dropwise. The mixture was left to stir overnight at room temperature under nitrogen. The solution was reduced to near dryness under vacuum and hexane was added to precipitate the mixture of [2+2] and [3+3] macrocycles as a colourless solid, which was collected by filtration. (1.44 g, 42% yield). **^{1}H NMR** (400 MHz, $CDCl_3$) δ 8.22 (s, 1H, [2+2]), 8.10 (s, 1.3H, [3+3]), 7.86 (t, *J* = 1.3 Hz, 0.52H, [2+2]), 7.82 (t, 0.55H, [3+3]), 7.75-7.67 (broad s, 0.85H, [3+3]), 7.66 (d, *J* = 1.3 Hz, 1H, [2+2]), 3.37 (m, 2.2H, [2+2] and [3+3]), 1.99 – 1.22 (m, cyclo-hex, 12H, [2+2] and [3+3]). Integrals are calibrated to 1H for the H_a peak for the [2+2] macrocycle to show relative amounts of each species in the mixture; peak integrals would be expected to have a ratio of 2:3 for [2+2]:[3+3]. See Figure S4 and S5 for integrations calibrated to each species; **^{13}C NMR** (101 MHz, $CDCl_3$) δ_C

160.09 [2+2], 158.32 [3+3], 138.28 [2+2], 138.22 [3+3], 132.04 [2+2], 126.60 [2+2], 126.14 [3+3], 123.14 [2+2], 122.94 [3+3], 74.45 [3+3], 74.07 [3+3], 32.92 [3+3], 32.26 [2+2], 24.49 [2+2], 24.33 [3+3]; **HRMS** (ES+) calc. for 100% [M+1] peak = [2+2] $C_{28}H_{31}Br_2N_4$: 583.0895, [3+3] $C_{42}H_{46}Br_3N_6$: 873.1314, [4+4] $C_{56}H_{61}Br_4N_8$: 1165.1712; m/z found for $[M+1]^+$ = [2+2]: 583.0887, [3+3]: 873.1296, [4+4]: 1165.1686

Values for [3+3] match that reported in the literature.[1] The *S,S*-equilibrium mixture can be obtained by using *S,S*-CHDA instead, and all data is the same.

1.2.3 Characterisation data

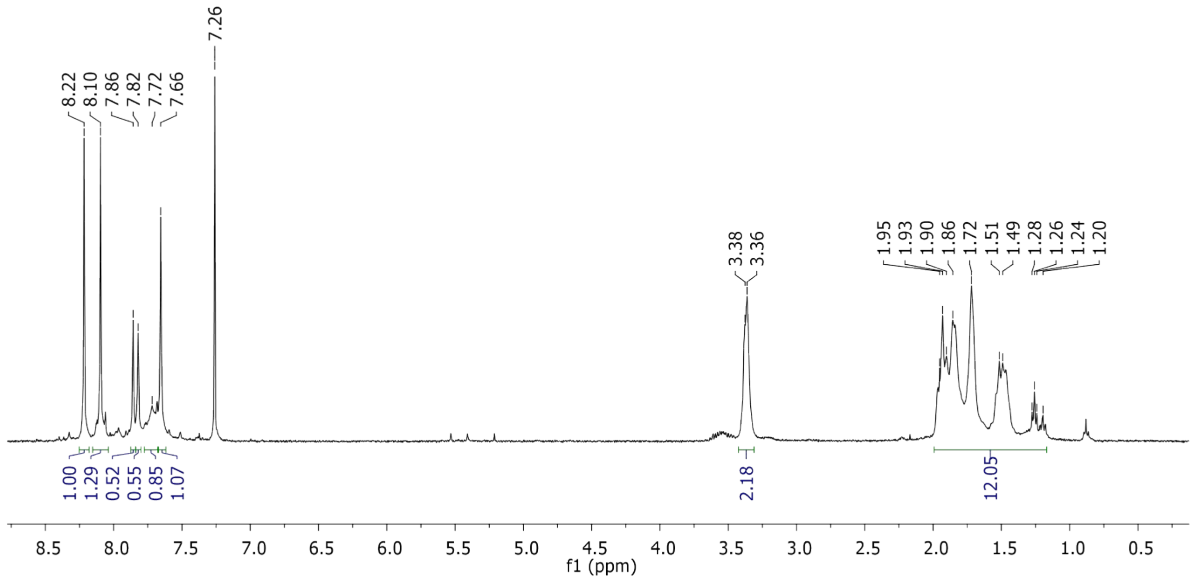

Figure S1: ^{1}H NMR spectrum ($CDCl_3$) of brominated isotrianglimine equilibrium mixture showing integrals as relative values for the [2+2] and [3+3] species, calibrated to 1H for the [2+2] macrocycle H_a peak.

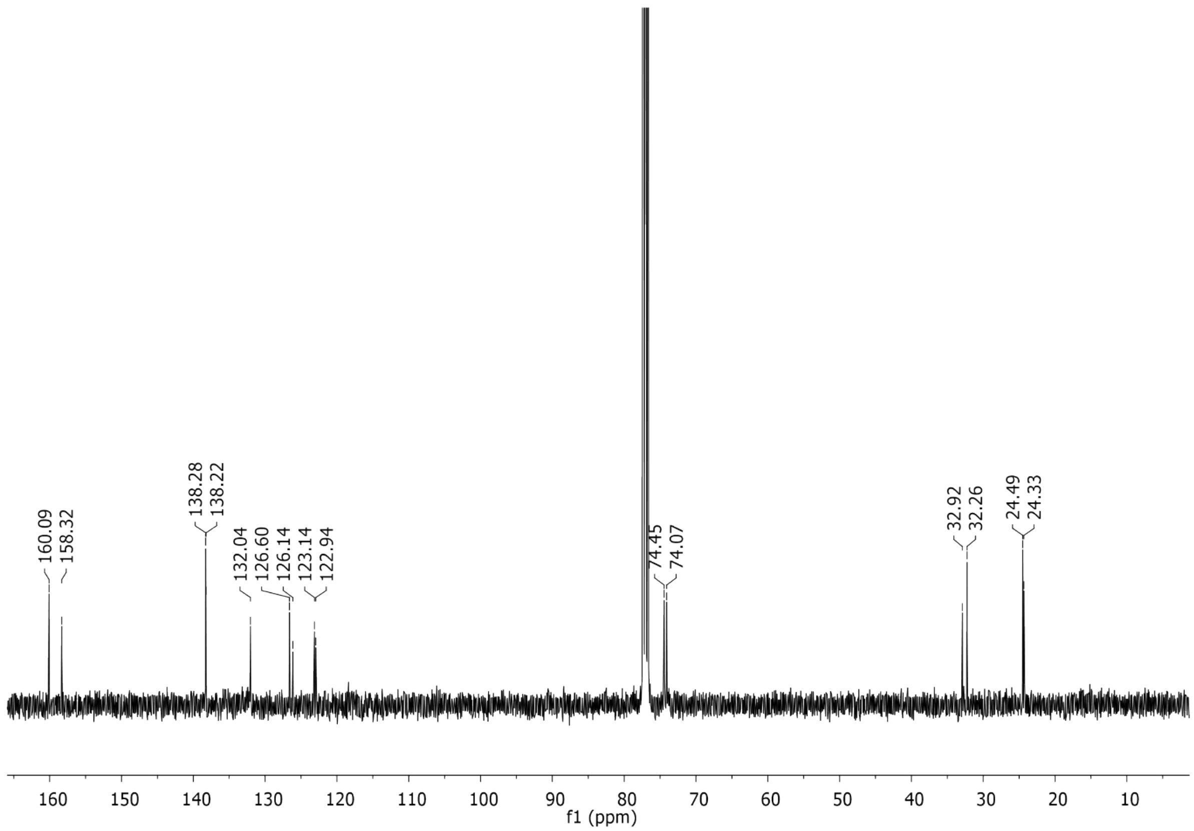

Figure S2: ^{13}C NMR spectrum ($CDCl_3$) of brominated isotrianglimine equilibrium mixture, 20 ppm – 170 ppm.

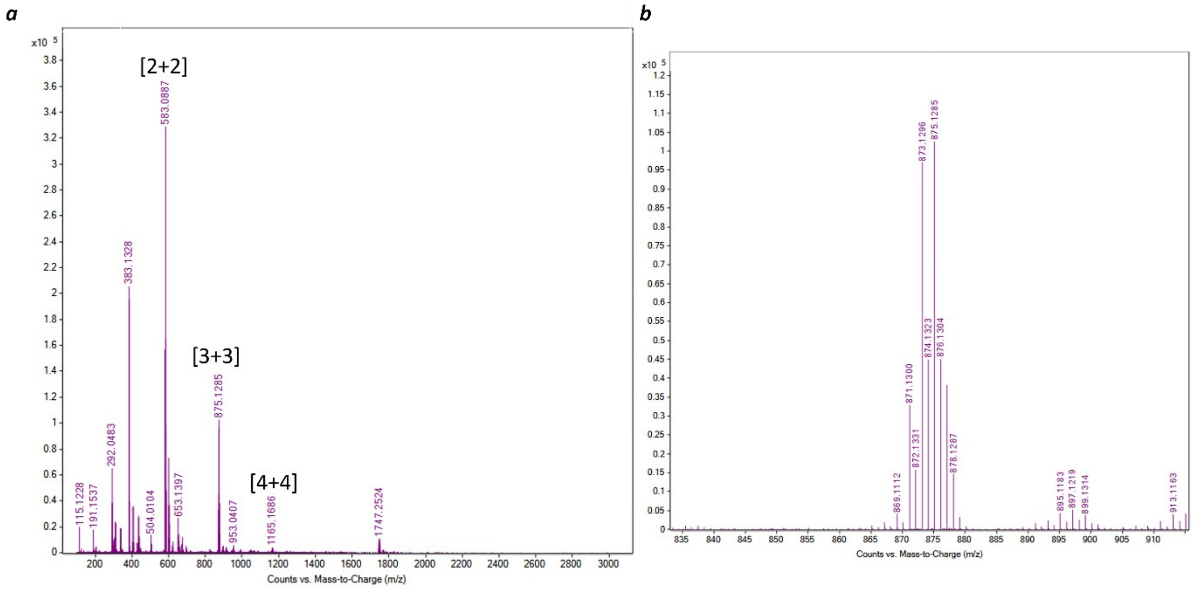

Figure S3: High resolution mass spectra for the brominated isotrianglimine crude reaction mixture, (a) showing the presence of [2+2], [3+3], and [4+4] macrocycles (b) showing the isotope splitting pattern for the [3+3] m/z peak.

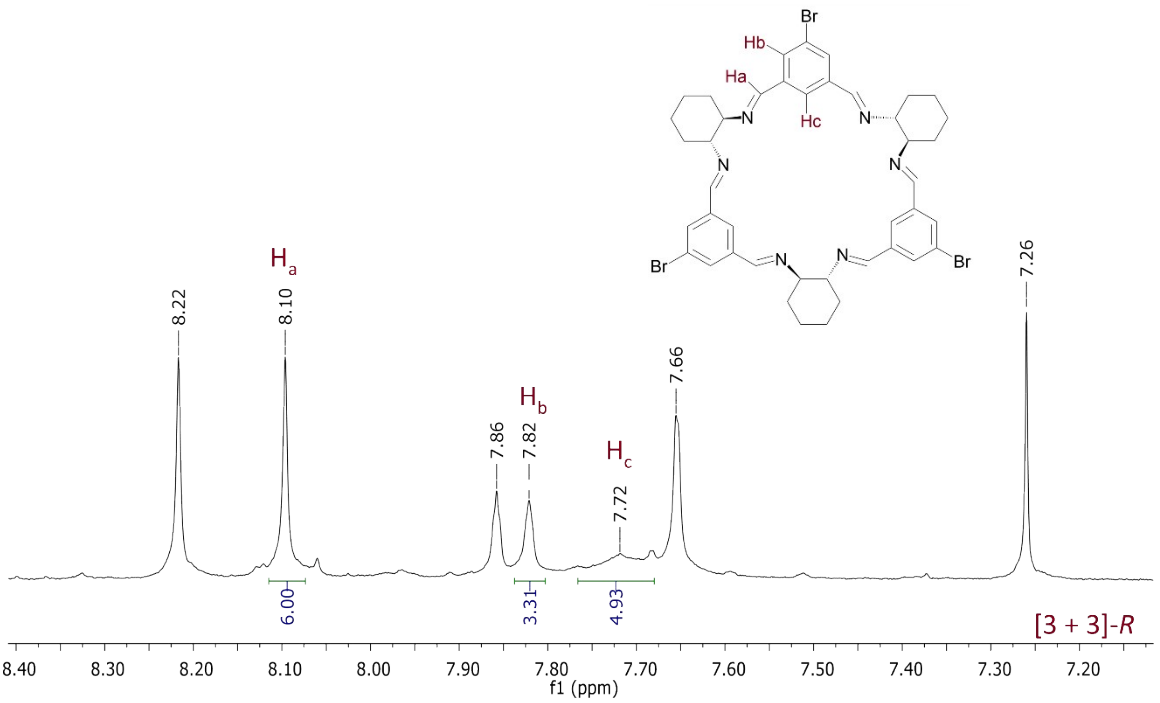

Figure S4: ^{1}H NMR ($CDCl_3$) spectrum with assignments with integrals shown calibrated to 6H for the [3+3] macrocycle H_a peak, aromatic region 7.60 ppm – 8.30 ppm.

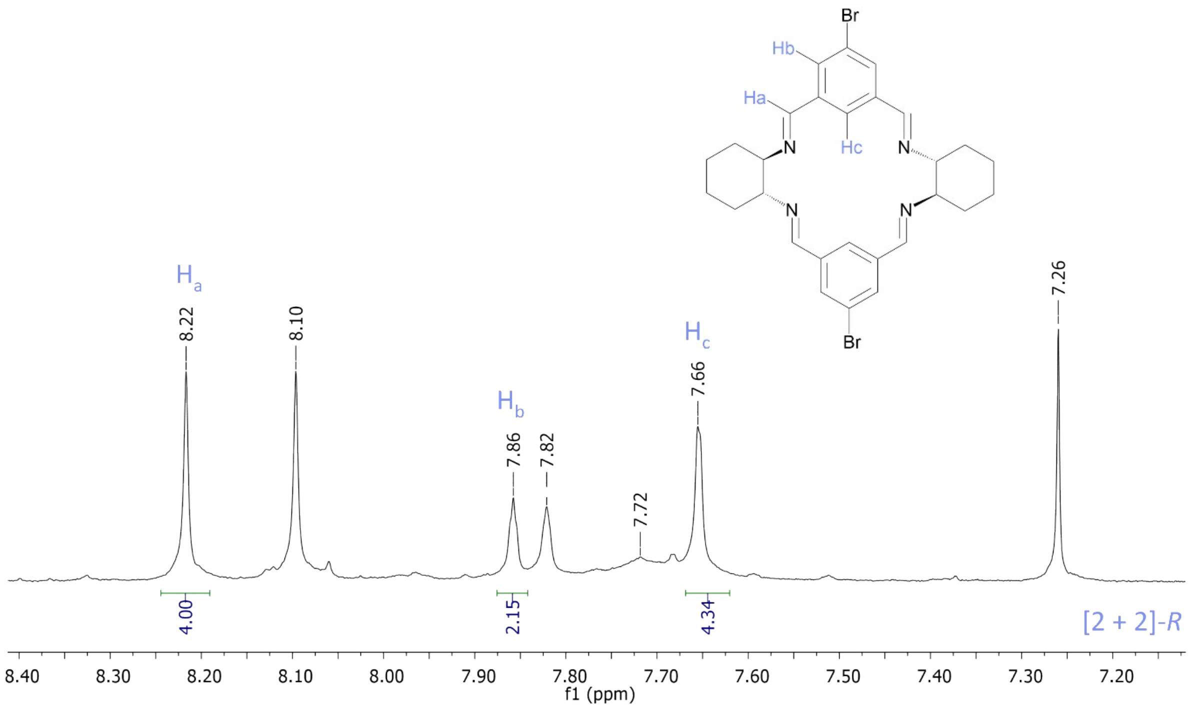

Figure S5: ^{1}H NMR with assignments and with integrals shown calibrated to 4H for the [2+2] macrocycle H_a peak, aromatic region, 7.60 ppm – 8.30 ppm.

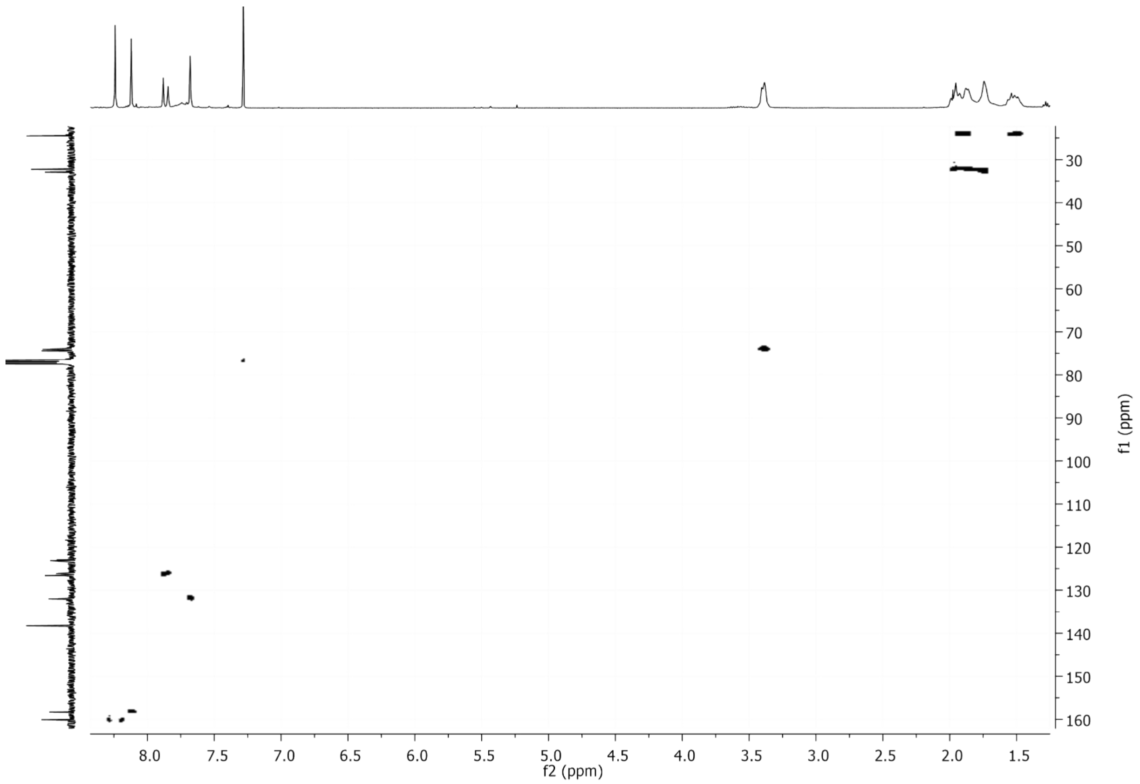

Figure S6: HSQC ($CDCl_3$) spectrum of the brominated isotrianglimine equilibrium mixture.

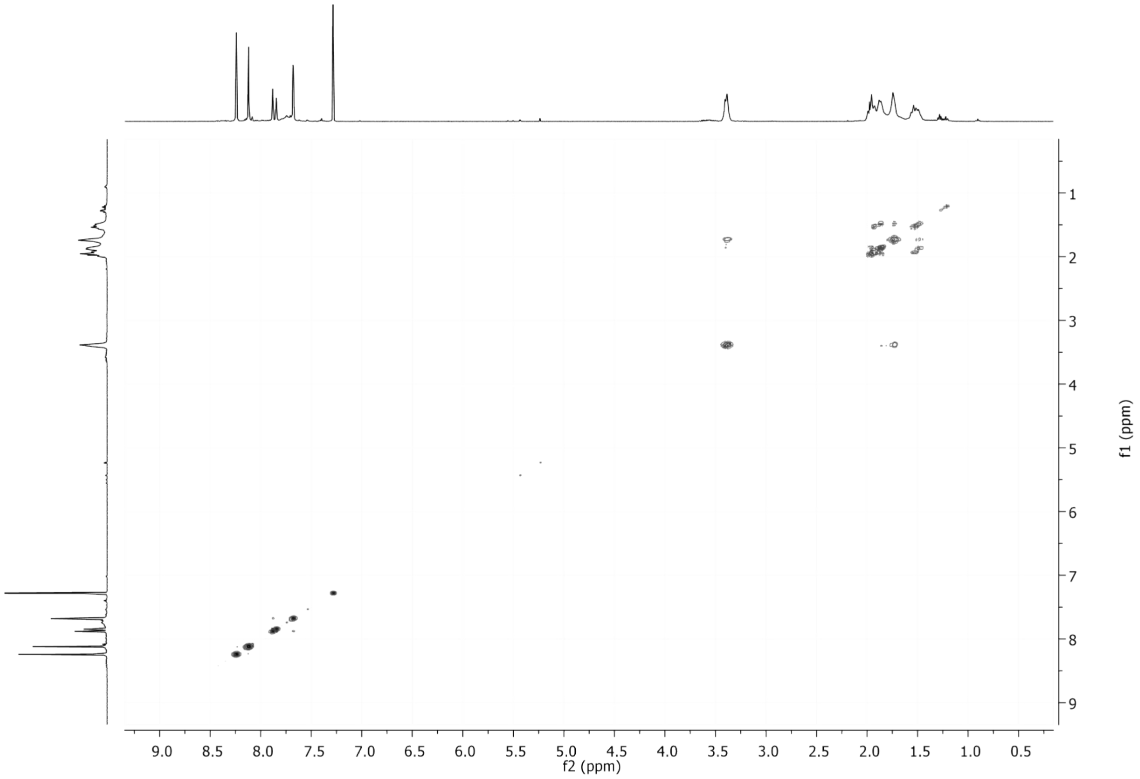

Figure S7: COSY ($CDCl_3$) spectrum on the brominated isotrianglimine equilibrium mixture – containing both [2+2] and [3+3] species.

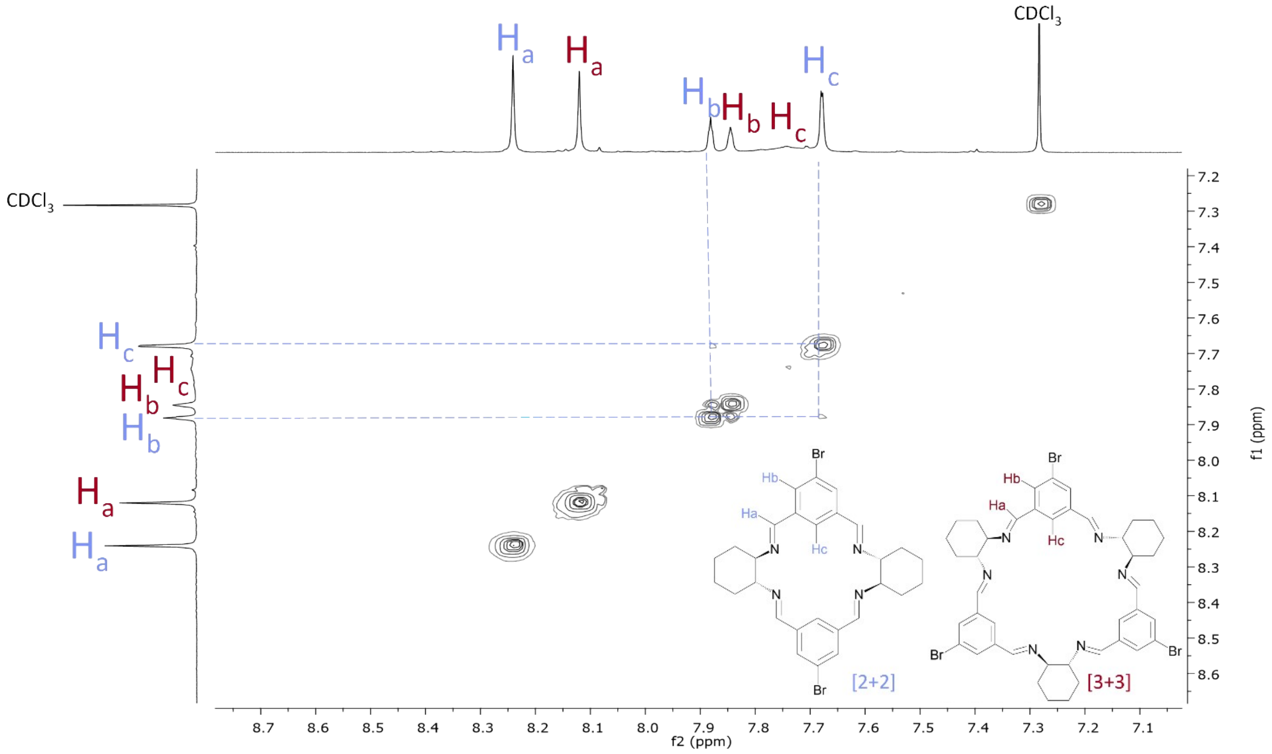

Figure S8: Aromatic region of the COSY ($CDCl_3$) spectrum of the brominated isotrianglimine equilibrium mixture, 7.00 ppm – 8.70 ppm.

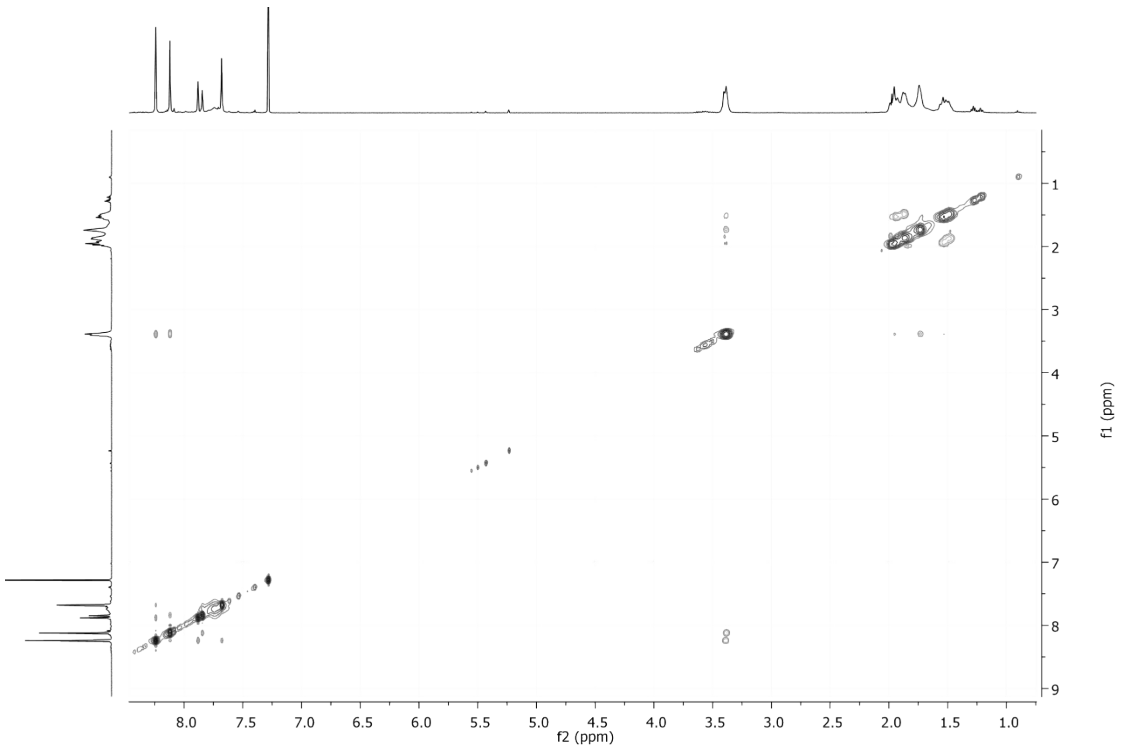

Figure S9: NOESY ($CDCl_3$) spectrum on brominated isotrianglimine equilibrium mixture containing both [2+2] and [3+3] macrocycles.

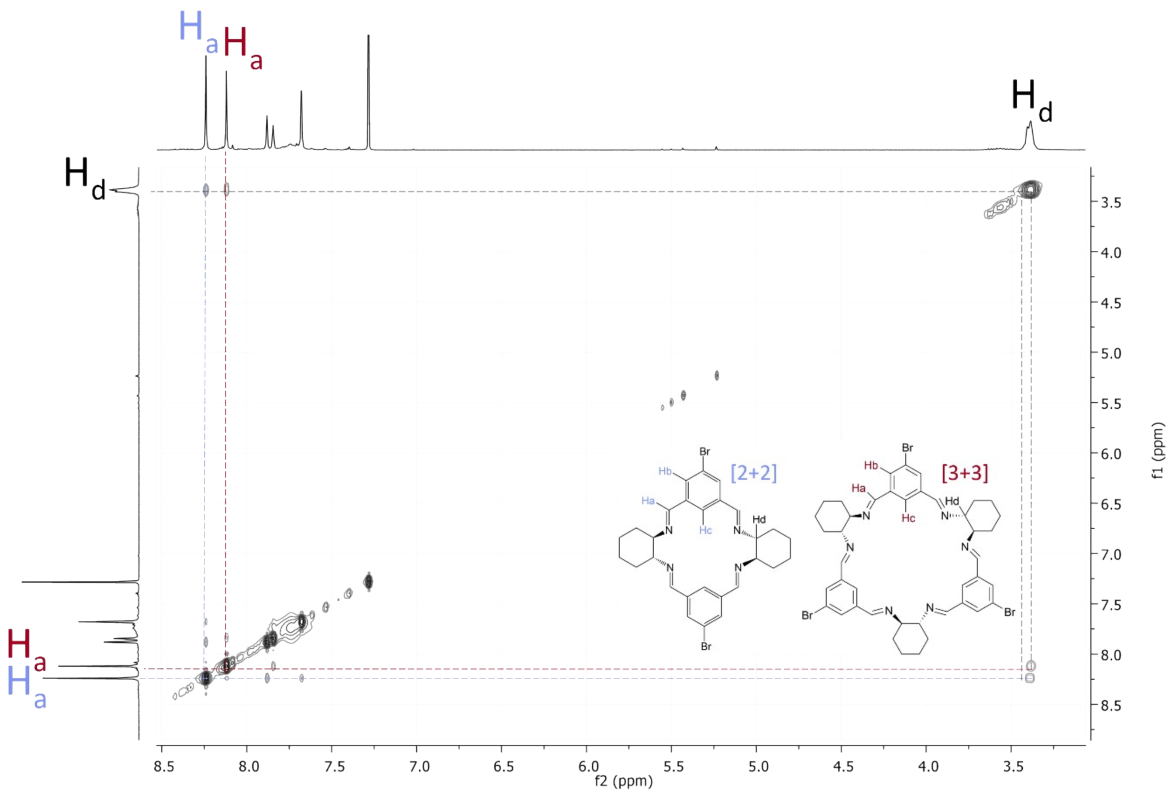

Figure S10: NOESY ($CDCl_3$) spectrum on equilibrium mixture [2+2] (blue) and [3+3] (red). Highlighted are the corresponding imine H_a proton and cyclohexane ring –CH (H_d) NOE interaction (2.0 ppm – 8.5 ppm).

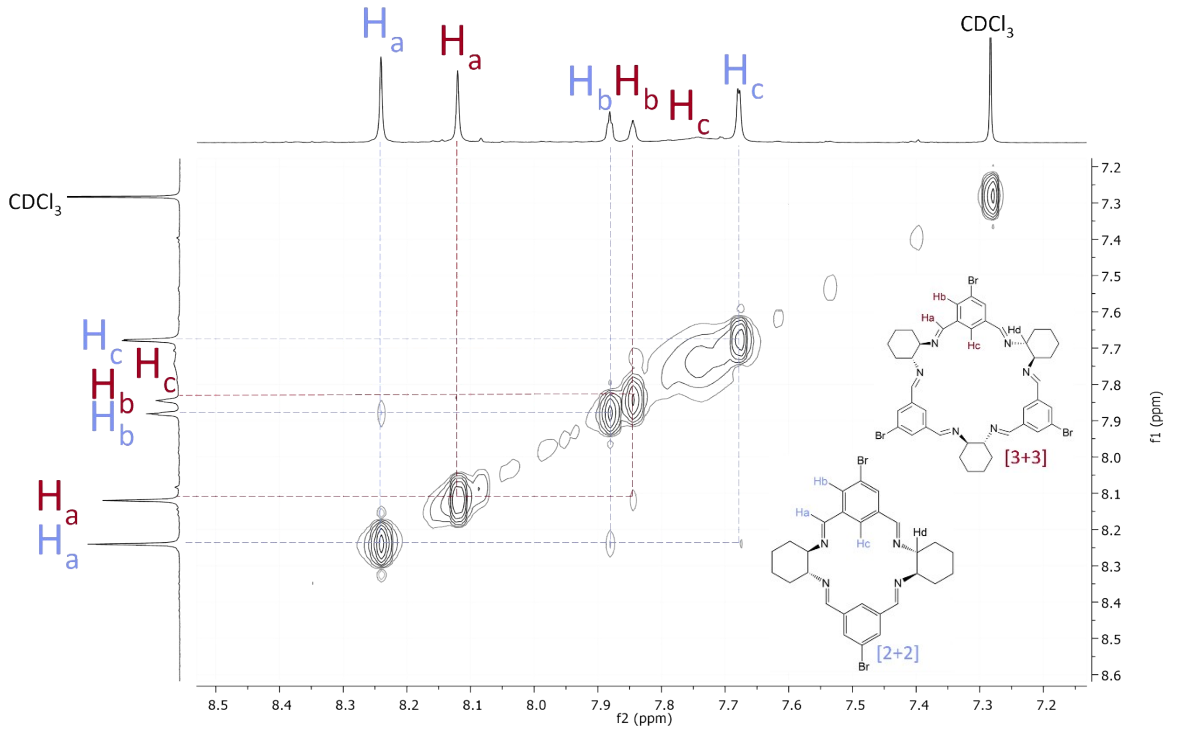

Figure S11: Aromatic region of the NOESY ($CDCl_3$) spectrum of the equilibrium mixture [2+2] (blue) and [3+3] (red).

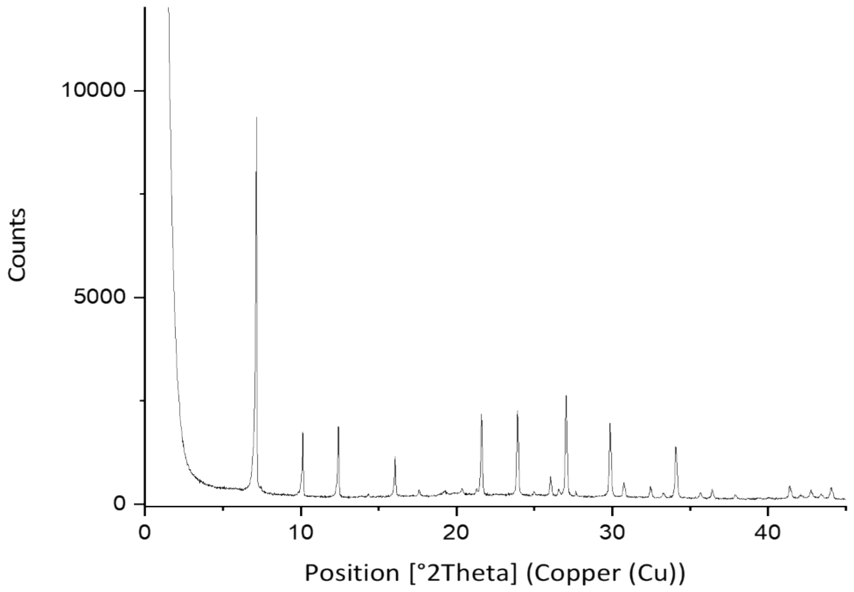

Figure S12: PXRD of equilibrium mixture [2+2] and [3+3] solid, obtained by reducing the chloroform reaction mixture to near dryness, precipitating with *n*-hexane and filtering to collect the solid.

Table 1: Different conditions screened for the synthesis of brominated isotrianglimine (bold indicates condition changed, green entries are shown in Figures S13 and S14):

Solvent	Amine Eq	Temperature	[2+2]:[3+3] ratio	Impurities
Me-THF	1	RT	1:1.70	Yes
DCM	1	RT	1:0.95	Yes
Toluene	1	RT	1:1.30	Yes
THF	1	RT	1:1.50	Yes
Chloroform	**1.05**	RT	1:0.70	Yes- small amount of SM aldehyde
Chloroform	**1.1**	RT	1:0.60	Yes - small amount of SM aldehyde
Toluene	1	**Reflux (70 hrs)**	1:0.88	Yes
THF	1	**Reflux (70 hrs)**	1:1.5	Yes - small amount of SM aldehyde
Chloroform	1	**Reflux (70 hrs)**	1:0.6	Yes - small amount of SM aldehyde

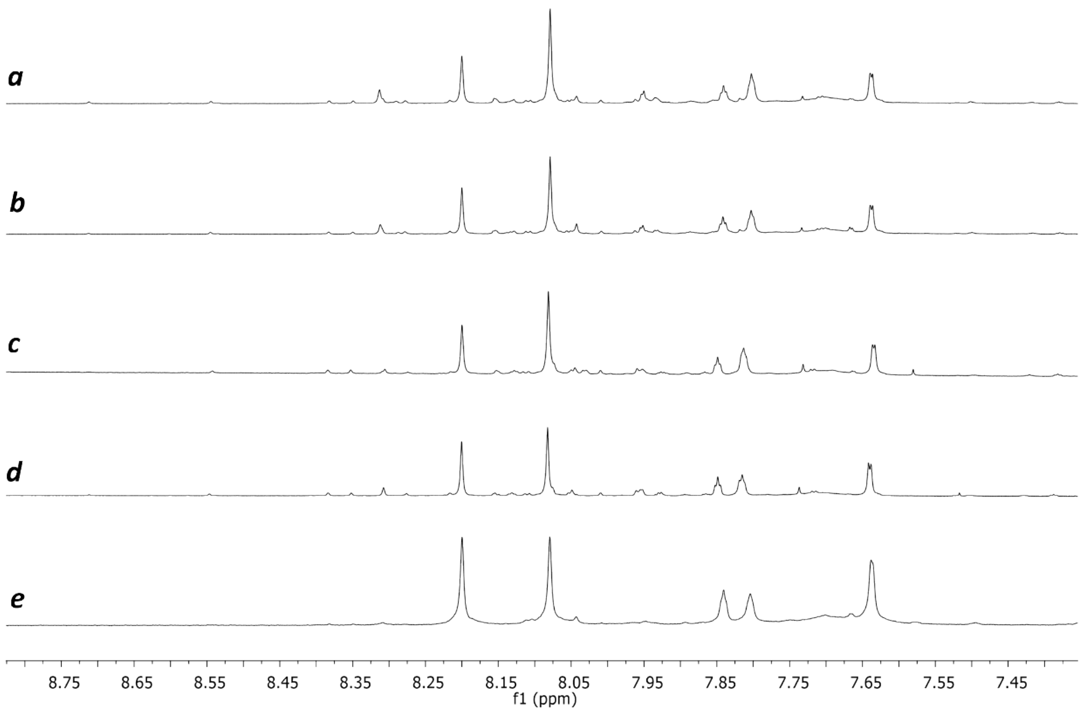

Figure S13: Stacked ^1H NMR ($CDCl_3$) spectra for the synthesis of brominated isotrianglimine in different solvents (RT) overnight; (a) THF; (b) toluene; (c) methyl THF; (d) dichloromethane; (e) chloroform. Spectra aligned to the [2+2] imine peak, 7.35 pm – 8.80 ppm.

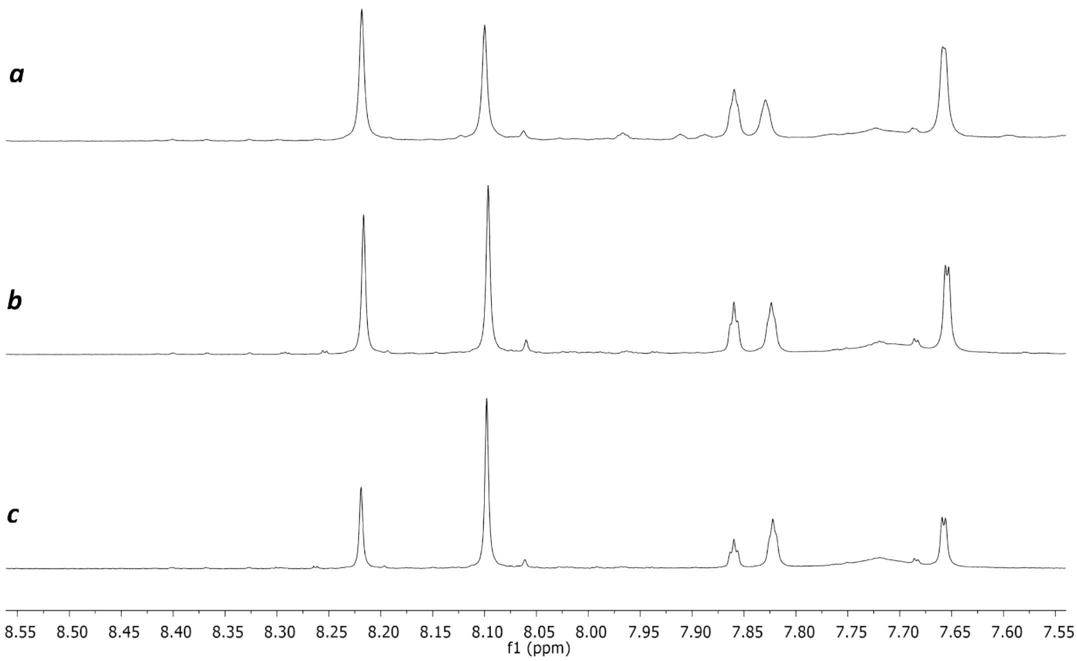

Figure S14: Stacked ^1H NMR ($CDCl_3$) spectra for the synthesis of brominated isotrianglimine refluxing in different solvents for 70 hours: (a) chloroform; (b) toluene; (c) THF, 7.55 ppm – 8.55 ppm.

1.3 Variable Temperature ^{1}H NMR

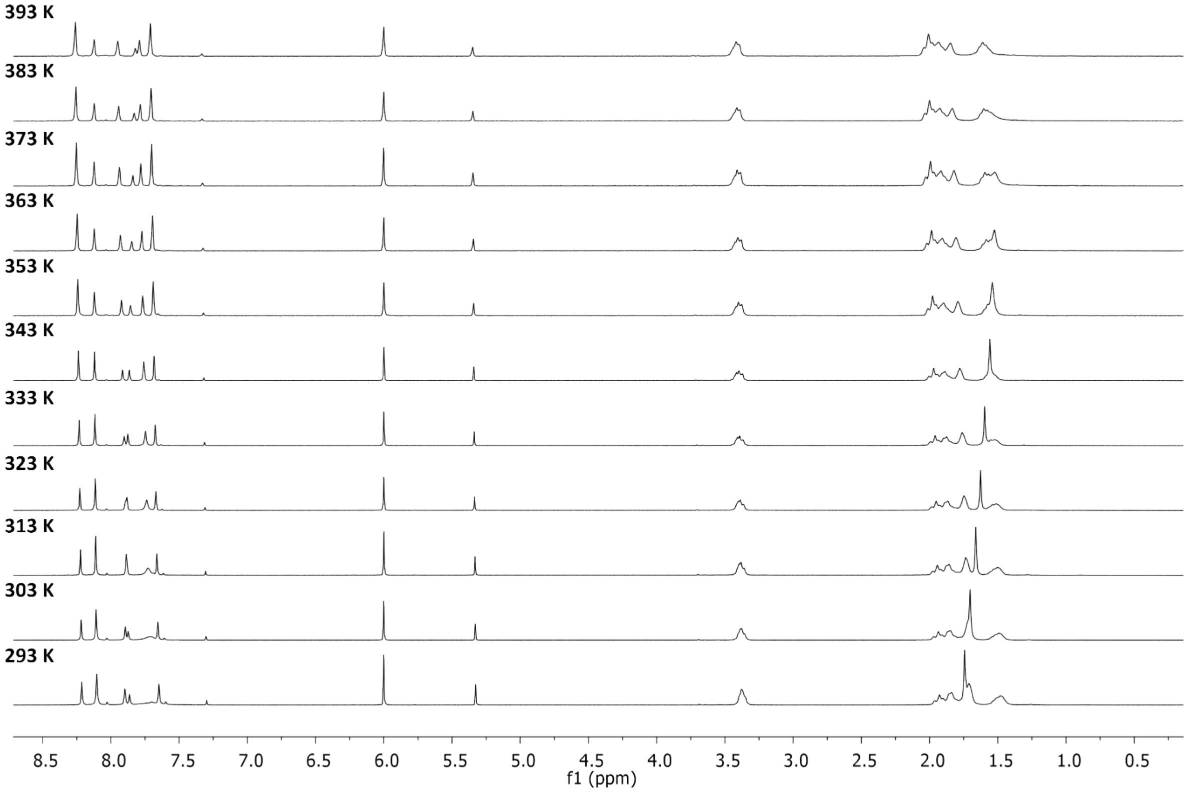

Figure S15: Hot VT NMR for the brominated isotrianglimine equilibrium mixture, 293 K – 393 K (tetrachloroethane-d_2). To obtain the NMR sample, a sample of recrystallised [3+3]-R was stirred in chloroform for 3 days to re-equilibrate to a mixture of [2+2] and [3+3]. The solution was then evaporated to dryness. The NMR sample was made from the solid sample (10 mg in 0.7 mL of TCE-d2).

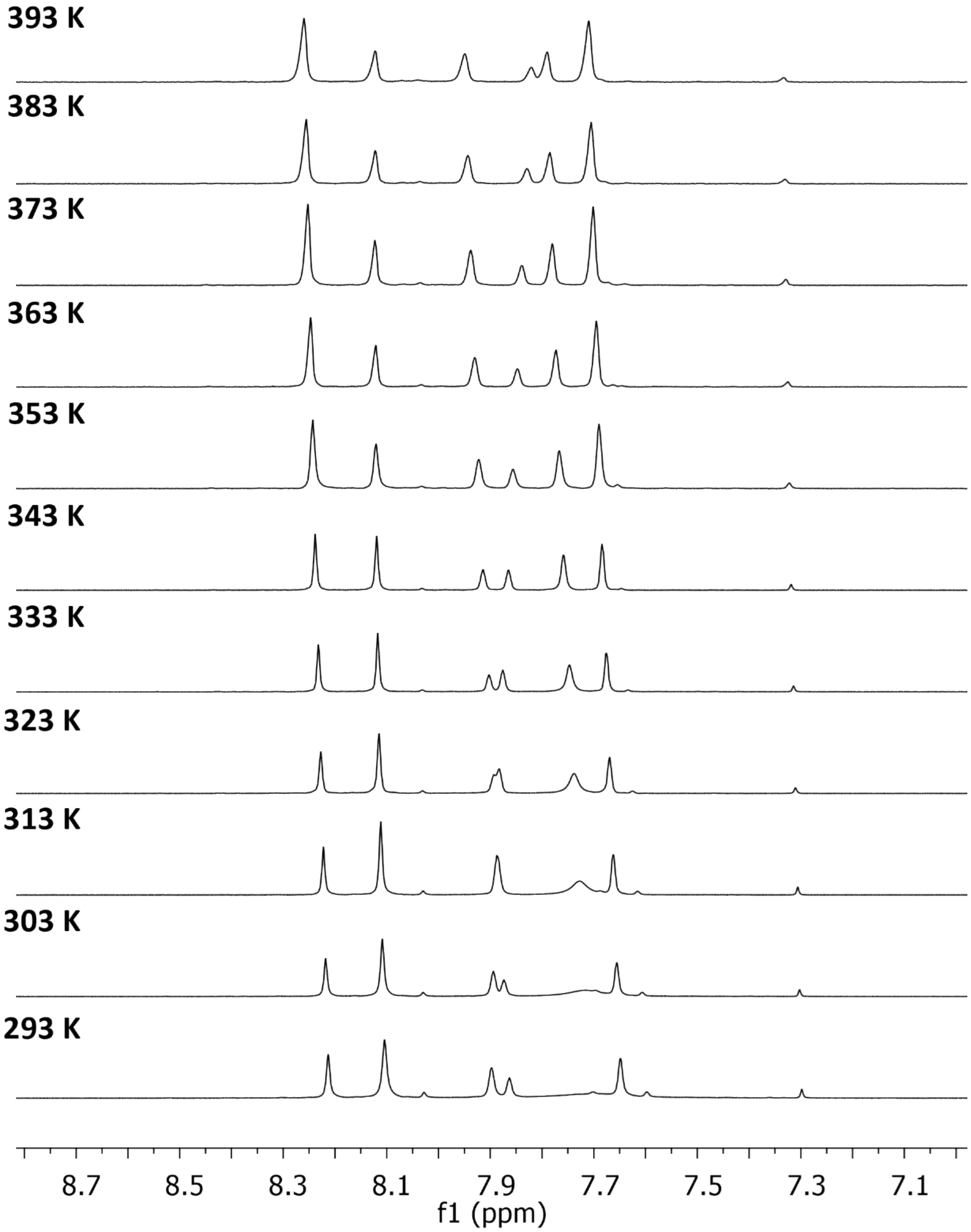

Figure S16: Hot VT NMR for the brominated isotrianglimine equilibrium mixture, 293 K – 393 K (tetrachloroethane-d_2), 6.75 ppm – 9.25 ppm.

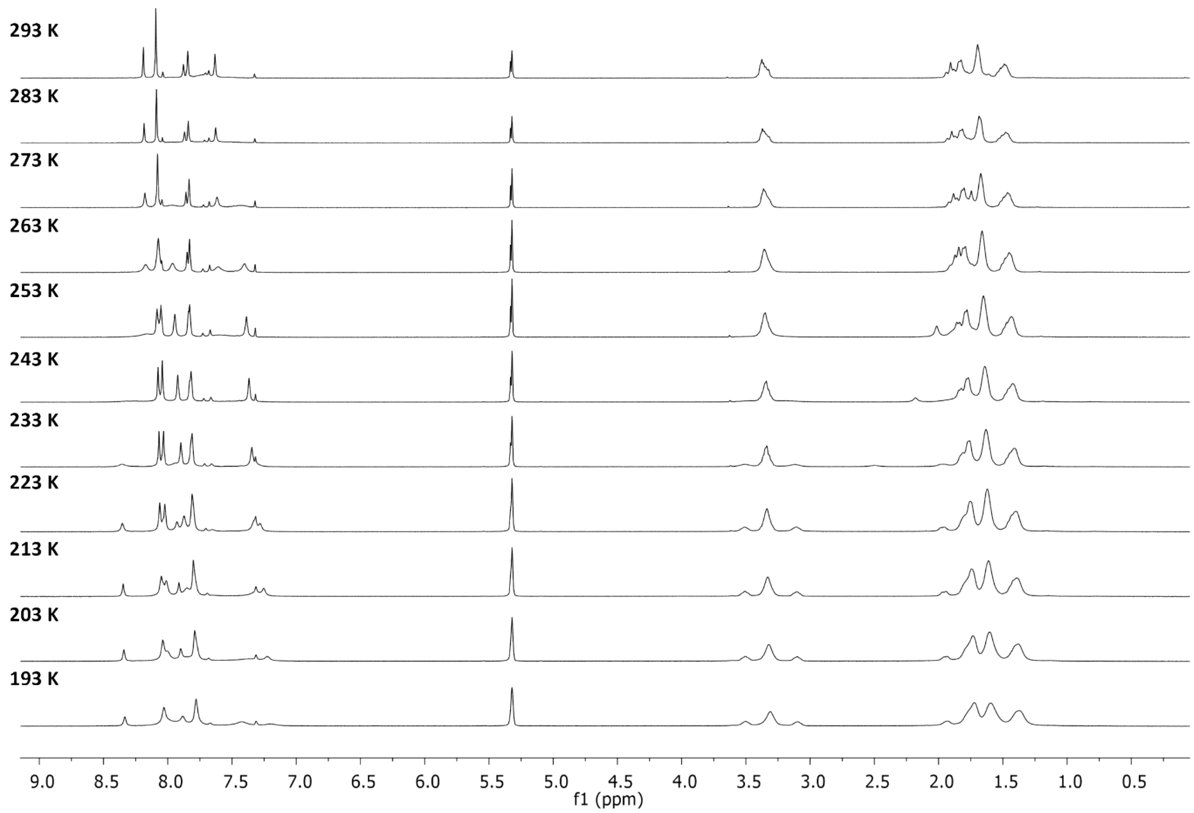

Figure S17: Cold VT NMR for the brominated isotrianglimine equilibrium mixture, 293 K – 193 K (dichloromethane-d_2). To obtain the NMR sample, a sample of recrystallised [3+3]-R was stirred in chloroform for 3 days to re-equilibrate to a mixture of [2+2] and [3+3]. The solution was then evaporated to dryness. The NMR sample was made from the solid sample (10 mg in 0.7 mL of DCM-d_2).

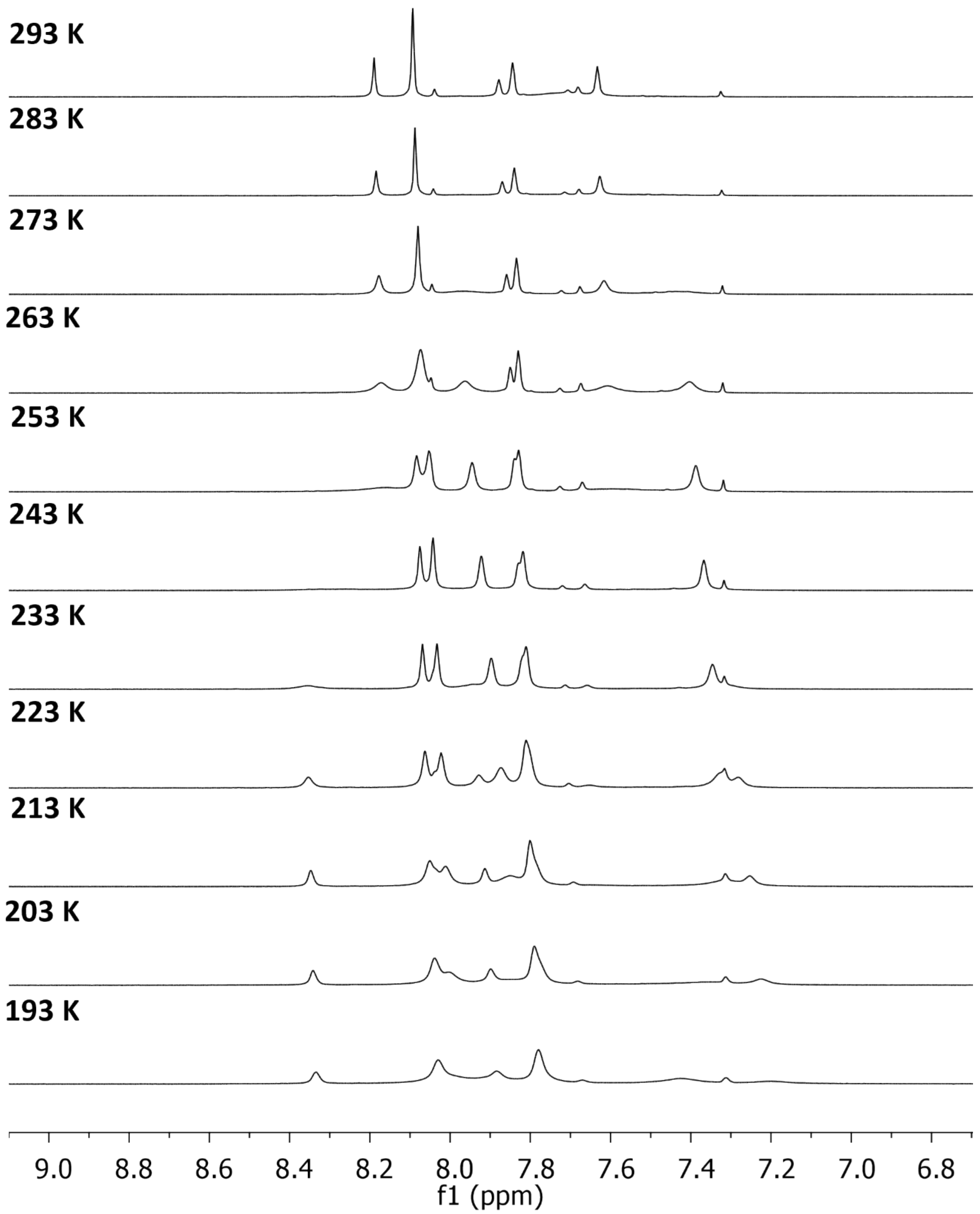

Figure S18: Cold VT NMR for the brominated isotrianglimine equilibrium mixture, 293 K – 193 K (dichloromethane-d_2). 6.75 ppm – 9.05 ppm.

1.3.1 Van't Hoff analysis

Integrals were obtained in TopSpin 4.2.0 for the two peaks corresponding to the [2+2] and [3+3] macrocycles. The peaks under investigation were the two most downfield signals in the ^{1}H NMR spectrum. We defined $I_{[2+2]} = 1$ and the corresponding $I_{[3+3]}$ can be found in the table below.

T/K	293	303	313	323	333	343	353	363	373	383	393
$I_{[3+3]}$	1.731	1.744	1.673	1.525	1.321	0.998	0.674	0.625	0.566	0.522	0.498

A van't Hoff plot was obtained for the values above using the following van't Hoff isochore, and assuming a) that all aldehyde monomer is either part of the [2+2] or [3+3] macrocycle, and b) that the total concentration of aldehyde monomer is constant throughout.

$$\ln\left[\frac{8I_{[3+3]}^{2}}{9I_{[2+2]}^{3}}\left(I_{[2+2]} + I_{[3+3]}\right)\right] = \frac{1}{R}\left(\Delta S^o - \frac{\Delta H^o}{T}\right)$$

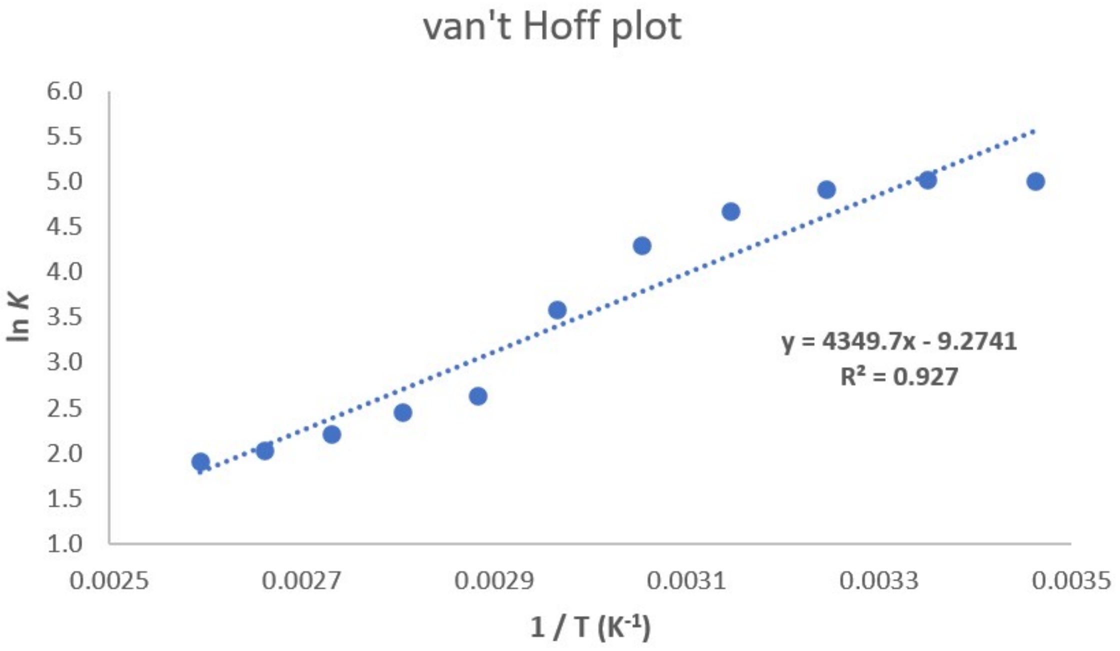

Figure S19: Van't Hoff plot for temperatures 393 K to 293K (1/T of 0.0026 to 0.0035 K^{-1}). A non-linear relationship is evident.

From the plot above, the corresponding enthalpy and entropy for the 3[2+2] ↔ 2[3+3] equilibrium are: ΔH^0 = - 36.2 kJ mol^{-1} and ΔS^0 = -77.1 J K^{-1} mol^{-1}. However, the non-linear

relationship implies that the assumption that only two macrocyclic species are involved in this process at all temperatures studied is likely not satisfied. Thus, a van't Hoff plot for the region where a linear relationship is seen is given below in Figure S20.

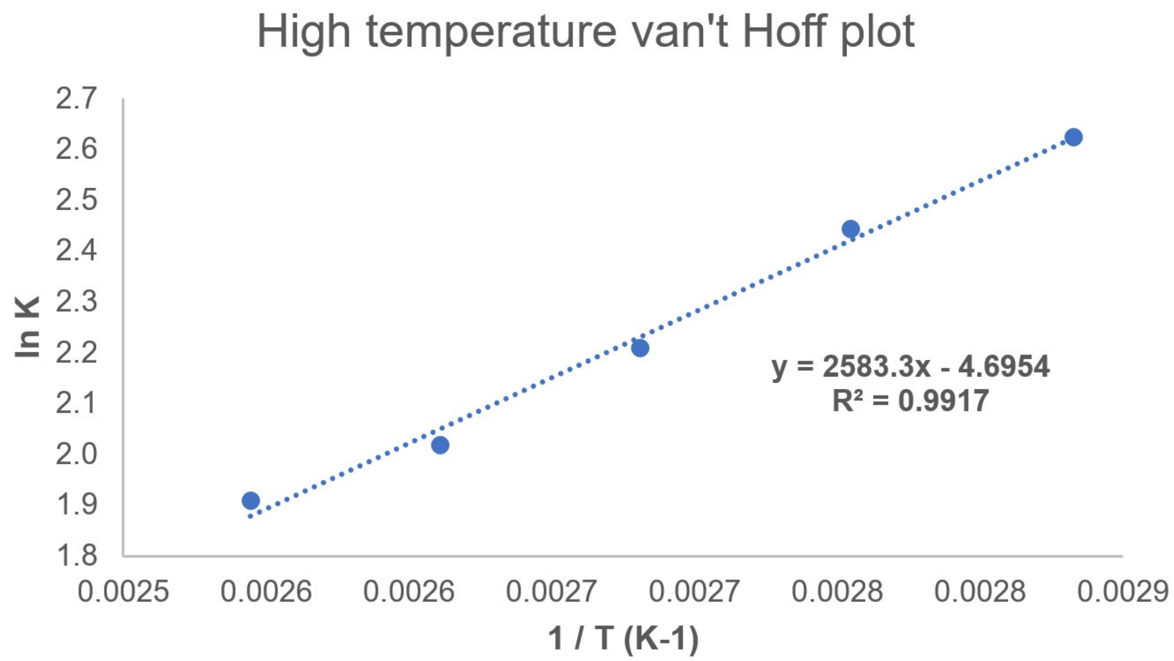

Figure S20: Van't Hoff plot for temperatures 393 K to 353K, corresponding to 1/T of 0.0026 - 0.0029 K^{-1}.

From the plot above, the corresponding enthalpy and entropy for the 3[2+2] ↔ 2[3+3] equilibrium are: ΔH^0 = - 21.5 kJ mol^{-1} and ΔS^0 = -39.0 J K^{-1} mol^{-1}.

At 298 K, TΔS = -11.6 kJ mol^{-1}.

1.4 Recrystallisation to [3+3]-*R*

The [3+3] macrocycle can be obtained by recrystalising the equilibrium reaction mixture from

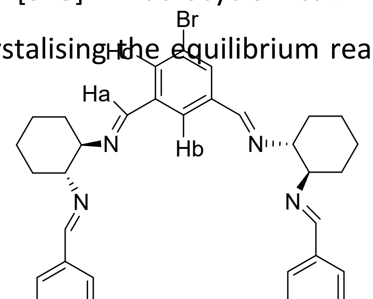

Section 1.2.2 in hot ethyl acetate and allowing it to cool to room temperature. The resulting precipitate was collected by filtration, washed with cold ethyl acetate and dried in a vacuum oven overnight at 50°C to afford a colourless solid. **^{1}H NMR** (400 MHz, $CDCl_3$) δ 8.10 (s, 6H), 7.82 (s, 3H), 7.72 (broad s, 5H), 3.41 – 3.32 (m, 6H), 1.85 (m, 6H), 1.72 (s, 12H), 1.54 – 1.42 (m, 6H); **^{13}C NMR** (101 MHz, $CDCl_3$) δ_C 158.32, 138.23, 126.13, 122.94, 77.33, 77.22, 77.02, 76.70, 74.44, 32.91, 24.33. Data matches that reported in the literature.[2]

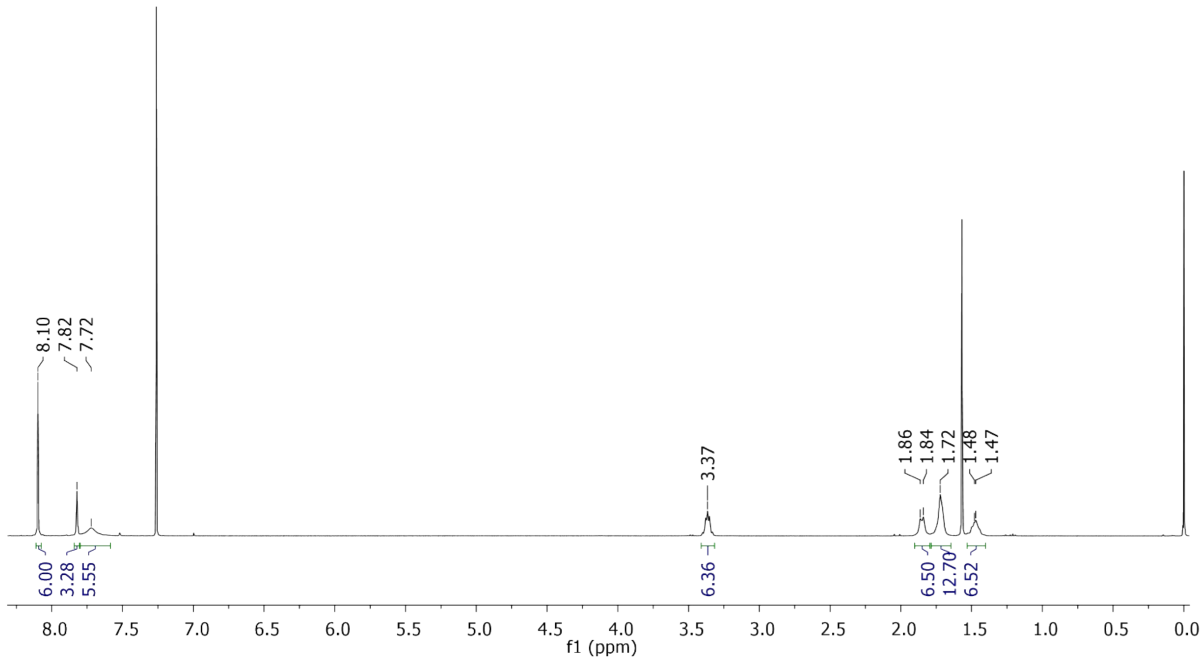

Figure S21: ^{1}H NMR spectrum ($CDCl_3$) of isolated [3+3]-*R* brominated isotrianglimine.

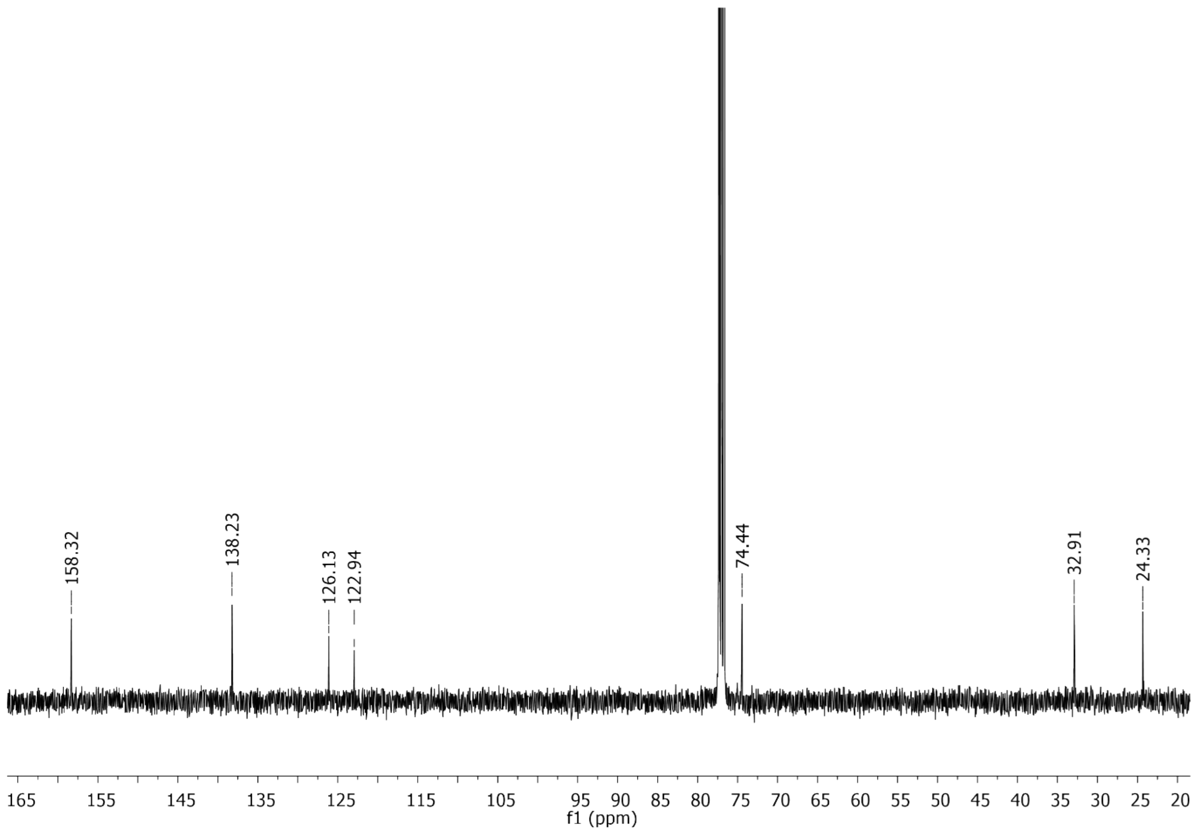

Figure S22: ^{13}C NMR ($CDCl_3$) of spectrum isolated [3+3]-*R* brominated isotrianglimine, 20 ppm – 165 ppm (zoomed in)

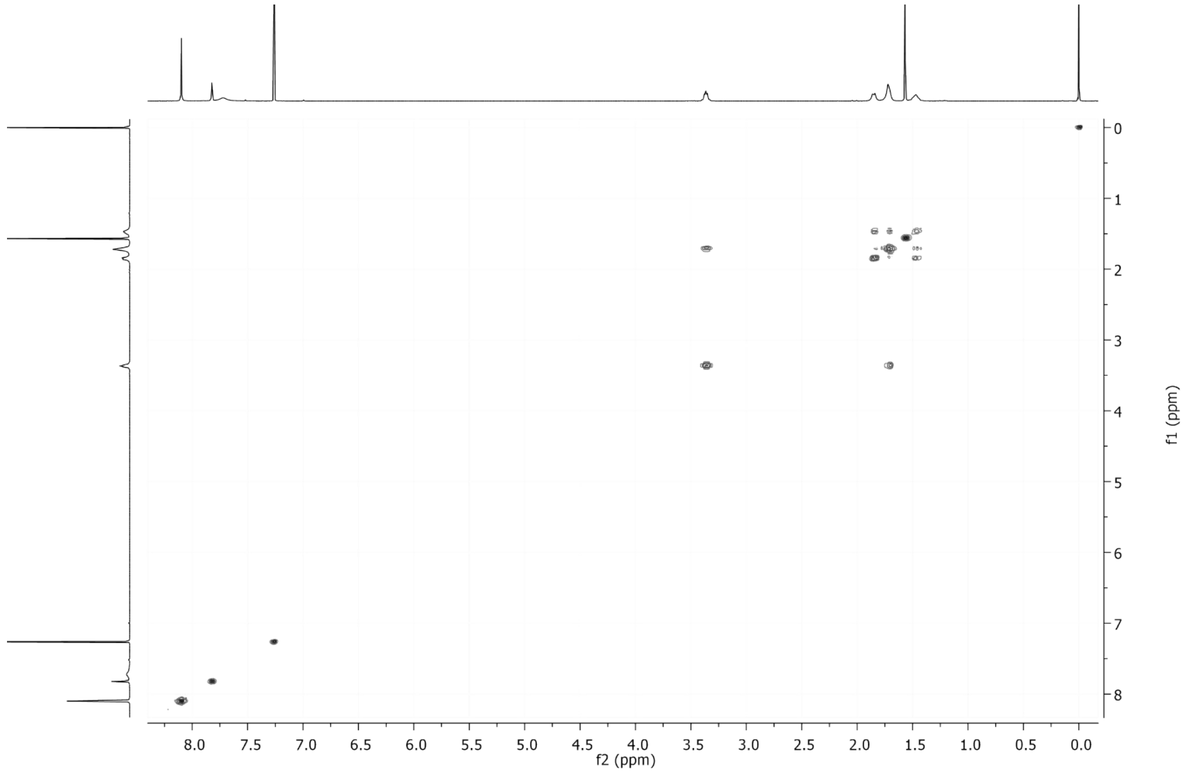

Figure S23: COSY ($CDCl_3$) spectrum of isolated [3+3]-*R* brominated isotrianglimine.

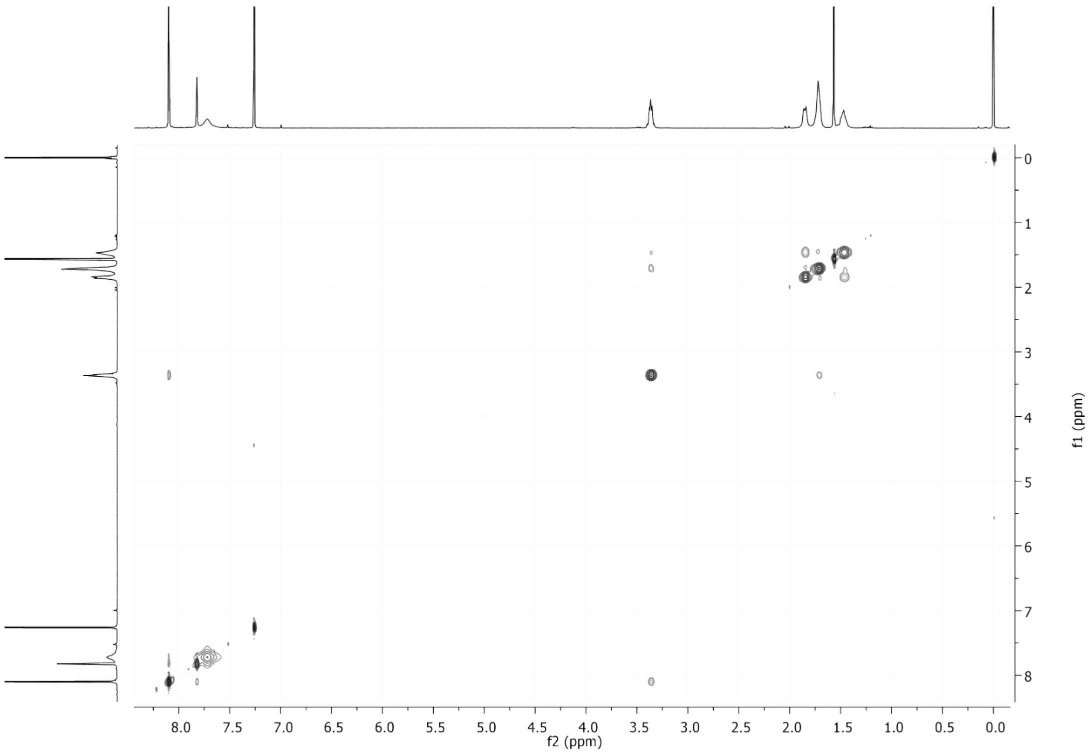

Figure S24: NOESY ($CDCl_3$) spectrum of isolated [3+3]-*R* brominated isotrianglimine.

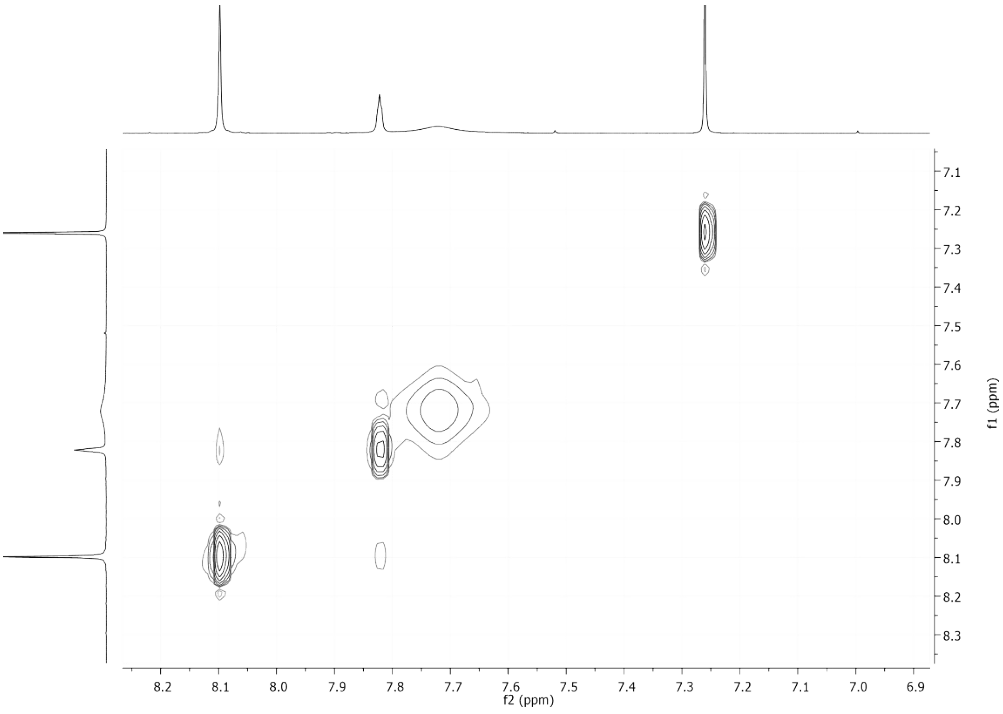

Figure S25: Aromatic region of NOESY ($CDCl_3$) spectrum of isolated [3+3]-*R* brominated isotrianglimine, 6.9 ppm – 8.3 ppm. The stability of [3+3]-*R* in solution was determined by repeatedly collecting ^1H NMR ($CDCl_3$) spectra at intervals over a week. Re-equilibration to the [2+2] and [3+3] equilbrium mixture was observed, but hydrolysis wasn't.

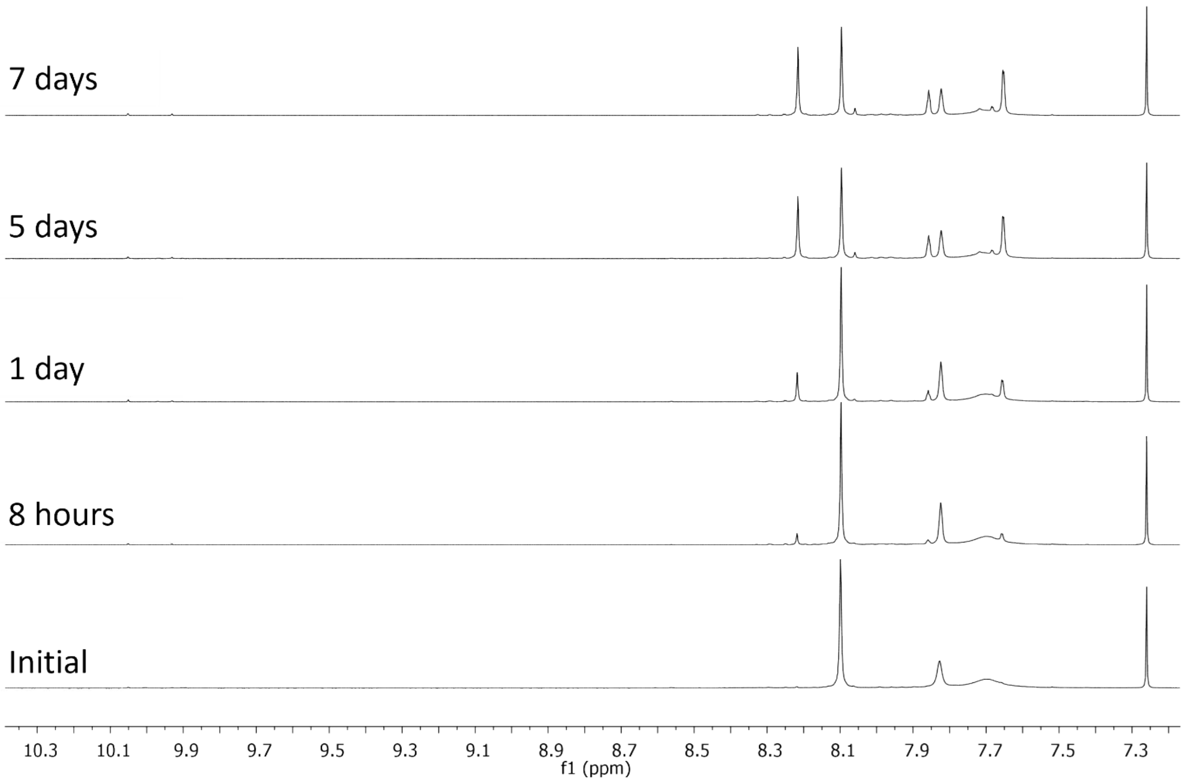

Figure S26: Stacked ^{1}H NMR ($CDCl_3$) spectra at intervals over a week, showing re-equilibration from the [3+3] macrocycle back to the equilibrium mixture of [2+2] and [3+3] species, 7.2 ppm – 10.30 ppm.

1.5 1,4-Dioxane solvent templation of [4+4]

[4 + 4] brominated isotrianglimine was obtained by dissolving either [3+3]-*R* brominated isotrianglimine or the equilibrium mixture containing both the [2+2] and [3+3] macrocycles in DCM (15 mg/mL). The solution was transferred to a small vial with a pierced lid, and placed in a larger vial containing 1,4-dioxane which was sealed. This resulted in a yellow crystal of [4+4] brominated isotrianglimine that was analysed by X-Ray diffraction and ^{1}H NMR spectroscopy.

The assignment of the aromatic region can be found in the main text.

^{1}H NMR (400 MHz, $CDCl_3$) δ 8.06 (s, 6H, H_A), 7.72 (d, *J* = 1.3 Hz, 4H, H_B), 7.68 (d, *J* = 1.4 Hz, 8H, H_C), 3.41 – 3.31 (m, 8H, H_D), 1.85 (d, *J* = 7.8 Hz, 12H, $H_{cyclo\text{-}hex}$), 1.73 (s, 21H, $H_{cyclo\text{-}hex}$), 1.47 (d, *J* = 4.0 Hz, 12H, $H_{cyclo\text{-}hex}$).

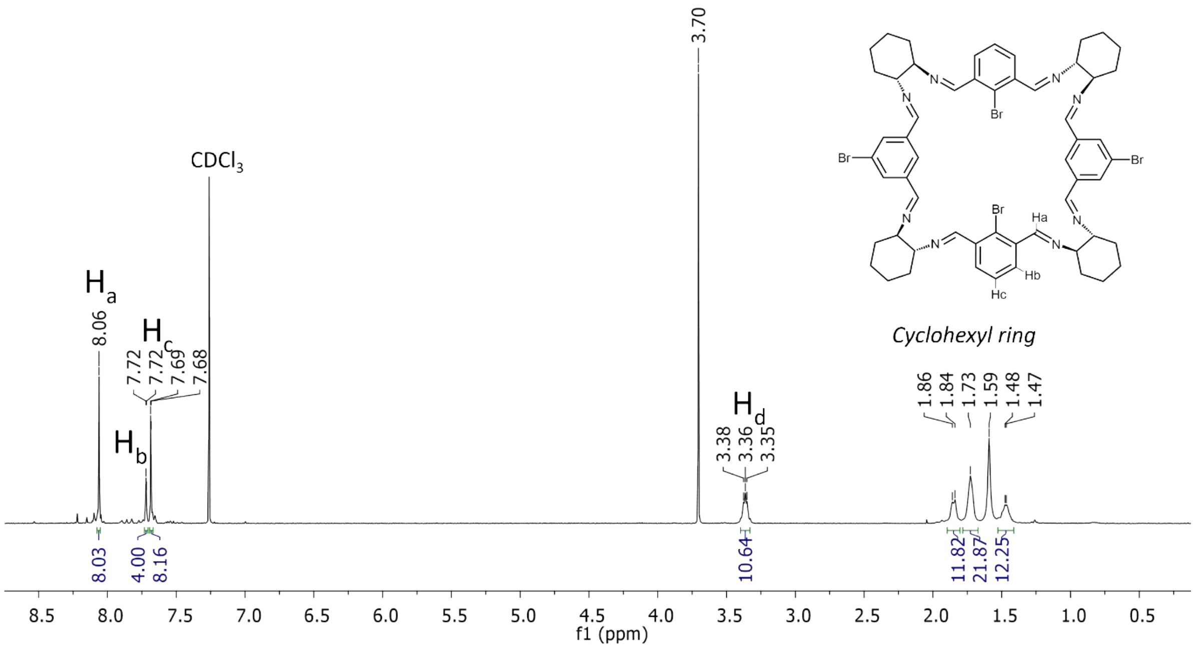

Figure S27: ^{1}H NMR ($CDCl_3$) spectrum for the isolated 1,4-dioxane templated [4+4] bromoisotrianglimine.

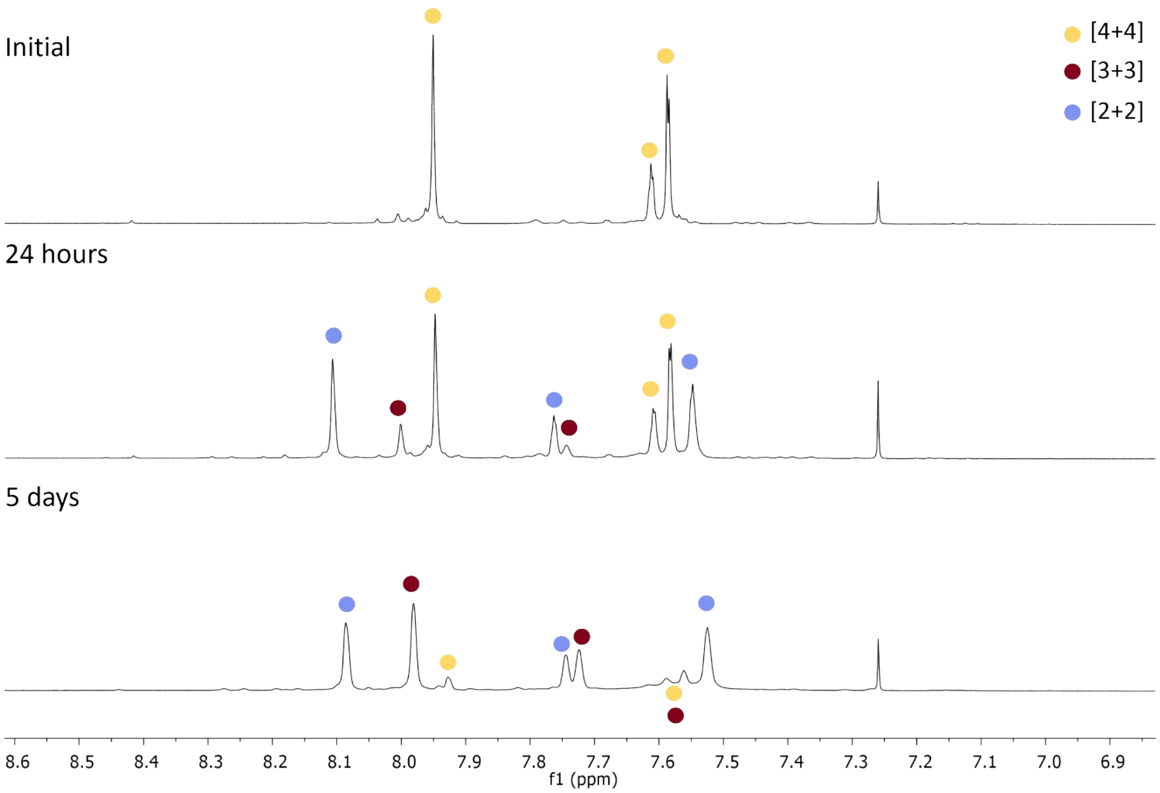

Figure S28: ^{1}H NMR ($CDCl_3$) spectrum for the isolated 1,4-dioxane templated [4+4] bromoisotrianglimine over a period of 5 days. Circles different colours for; [2+2] (blue), [3+3] (red) and [4+4] (yellow).

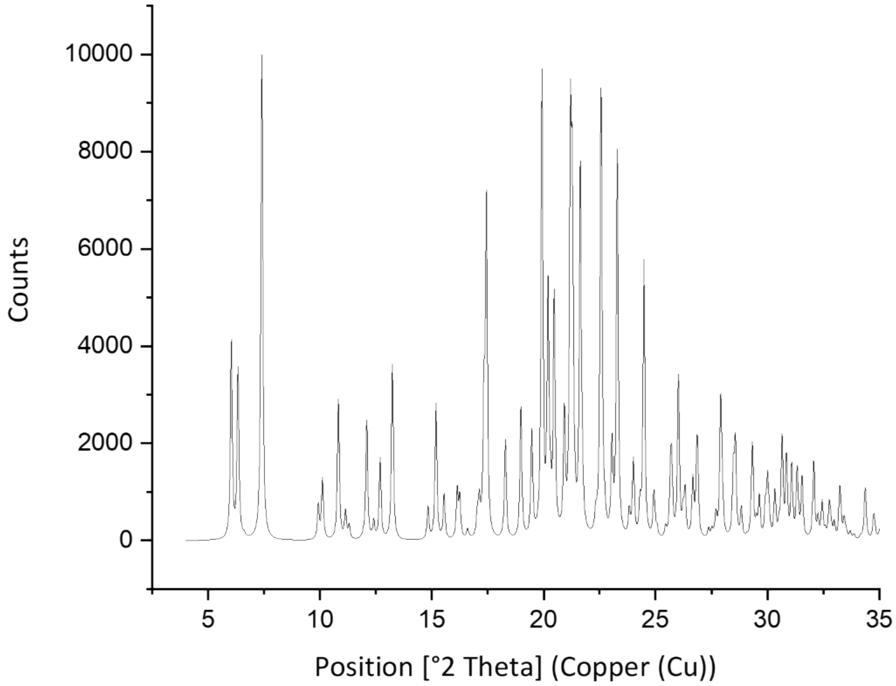

Figure S29: PXRD of [4+4]-*S* brominated isotrianglimine crystals obtained from DCM-1,4-dioxane. Laboratory diffractometer based PXRD experiments on [4+4]-*S*•(1,4-dioxane) were carried out at room temperature on a Bruker D8 Advance diffractometer with a monochromated Cu source (Kα1, λ = 1.5406 Å) in Debye-Scherrer geometry.

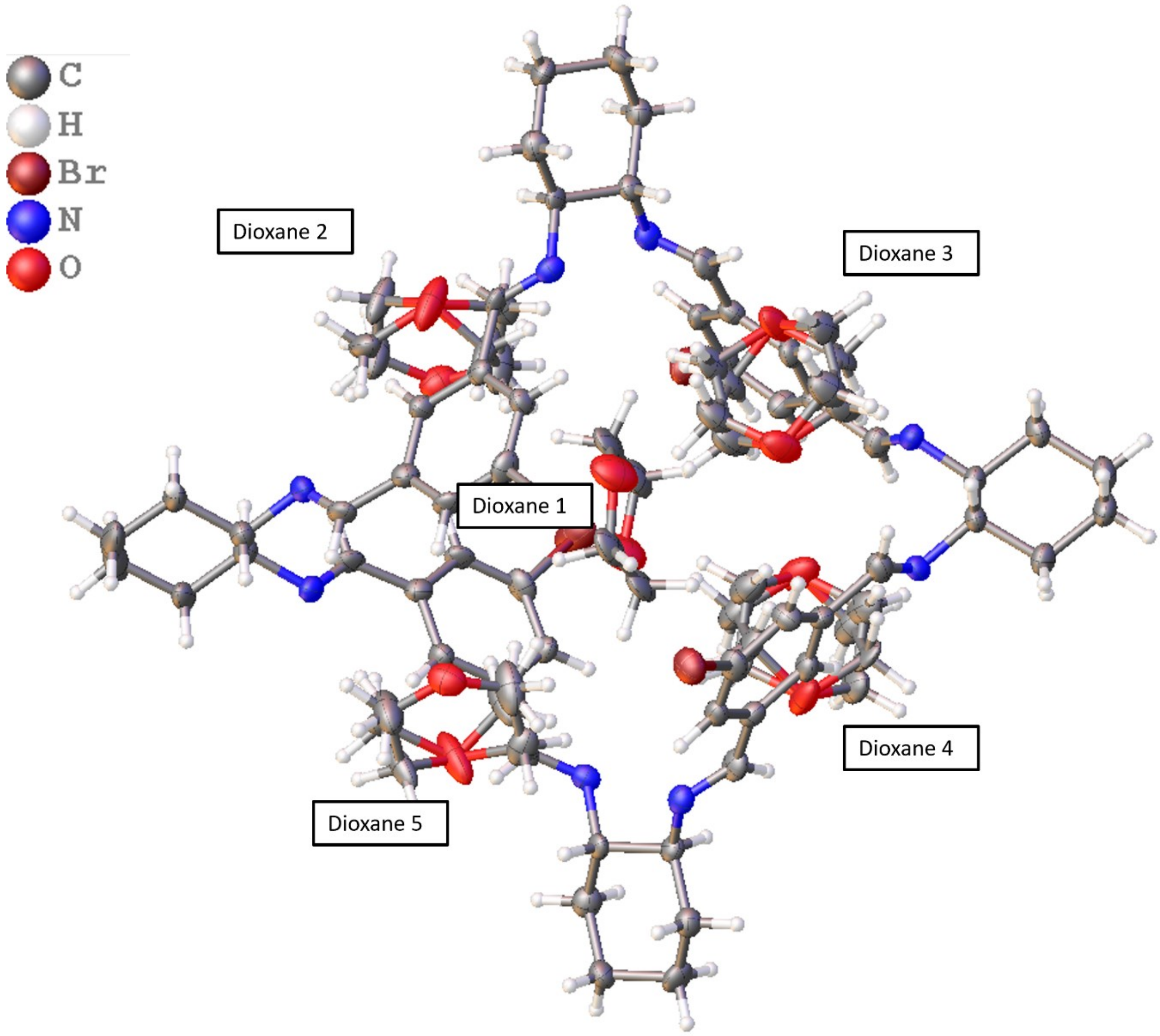

Figure S30: ORTEP structure of [4+4]-*S*. Dioxane 1 can be seen in the cavity of the macrocycle, as well as four peripheral dioxane molecules.

1.6 [3+3]-*R*/[3+3]-*S* and *meso*-[2 + 2]

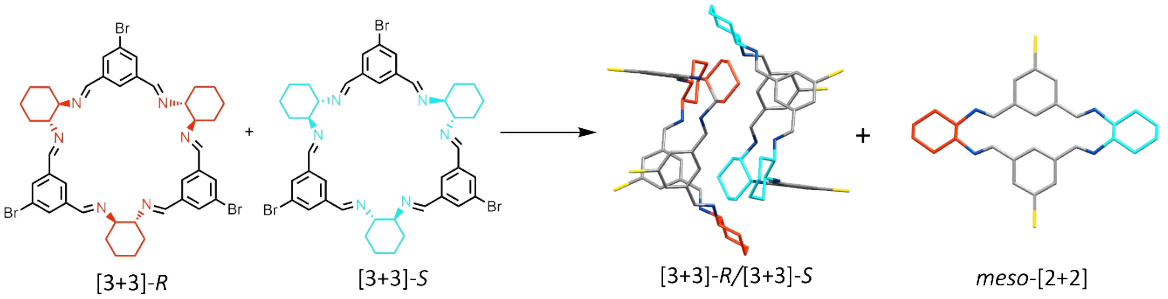

Figure S31: Co-crystallising [3+3]-*R* and [3+3]-*S* in ethyl acetate results in [3+3]-*R*/[3+3]-*S* and *meso*-[2+2]. Colours – *R,R*-cyclohexyl rings; red, *S,S*-cyclohexyl rings; cyan, nitrogen; blue, bromine; yellow.

[3+3]-*R* (1.00 g, 1.15 mmol, 1 eq) and [3+3]-*S* (1.00 g, 1.15 mmol, 1 eq) were dissolved in ethyl acetate (50 mL) at 80 °C and allowed to cool to room temperature. The resulting precipitate was collected by filtration, washed with cold ethyl acetate and dried in the vacuum oven overnight at 50 °C to obtain [3+3]-*R*/[3+3]-*S* as a colourless solid (0.84 g, 41 % yield). The original ethyl acetate solution was left to stand, resulting in further precipitation of the solid which was collected and analysed to be a mixture of [3+3]-*R*/[3+3]-*S* and *meso*-[2+2]. Eventually, *meso*-[2+2] precipitated exclusively and was obtained by collecting by filtration, washing with cold ethyl acetate, and drying in a vacuum oven overnight at 50 °C. *meso* [2+2] was obtained as a colourless solid (0.36 g, 18 % yield). After 1 week nothing else precipitated from the ethyl acetate solution; the remaining solution was analysed by ^{1}H NMR and found to include multiple racemic species of brominated isotrianglimine (Figure S38).

[3+3]-*R*/[3+3]-*S*:

^{1}H NMR is identical to the homochiral [3+3] spectra.

^{1}H NMR (400 MHz, $CDCl_3$) δ 8.10 (s, 6H), 7.82 (s, 3H), 7.72 (broad s, 5H), 3.41 – 3.32 (m, 6H), 1.85 (d, 6H), 1.72 (s, 12), 1.54 – 1.42 (m, 6H). **^{13}C NMR** (101 MHz, $CDCl_3$) δ_C 158.32, 138.23, 126.13, 122.94, 77.33, 77.22, 77.02, 76.70, 74.44, 32.91,

***Meso*-[2+2]:** **^{1}H NMR** δ 8.07 (d, *J* = 1.2 Hz, 4H), 7.61 (s, 4H), 6.35 (s, 2H), 3.21 – 3.13 (m, 4H), 2.01, 18H.

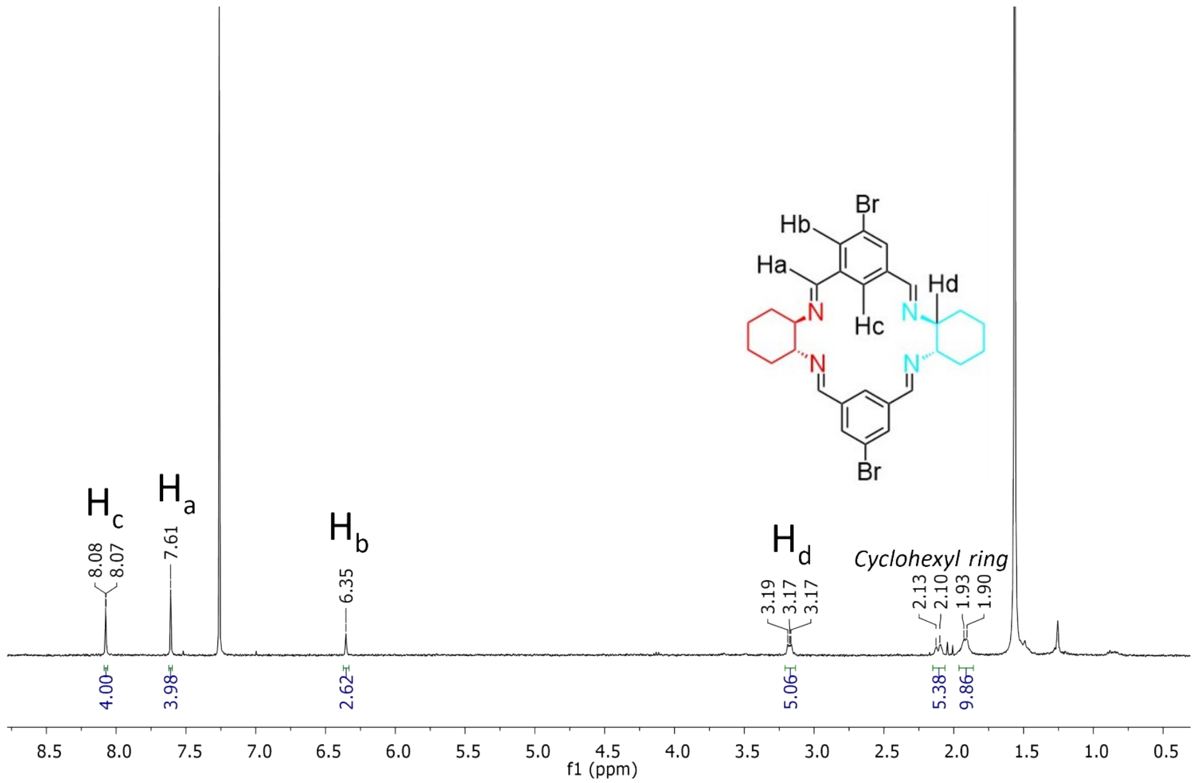

Figure S32: ^{1}H NMR ($CDCl_3$) spectrum for *meso*-[2+2].

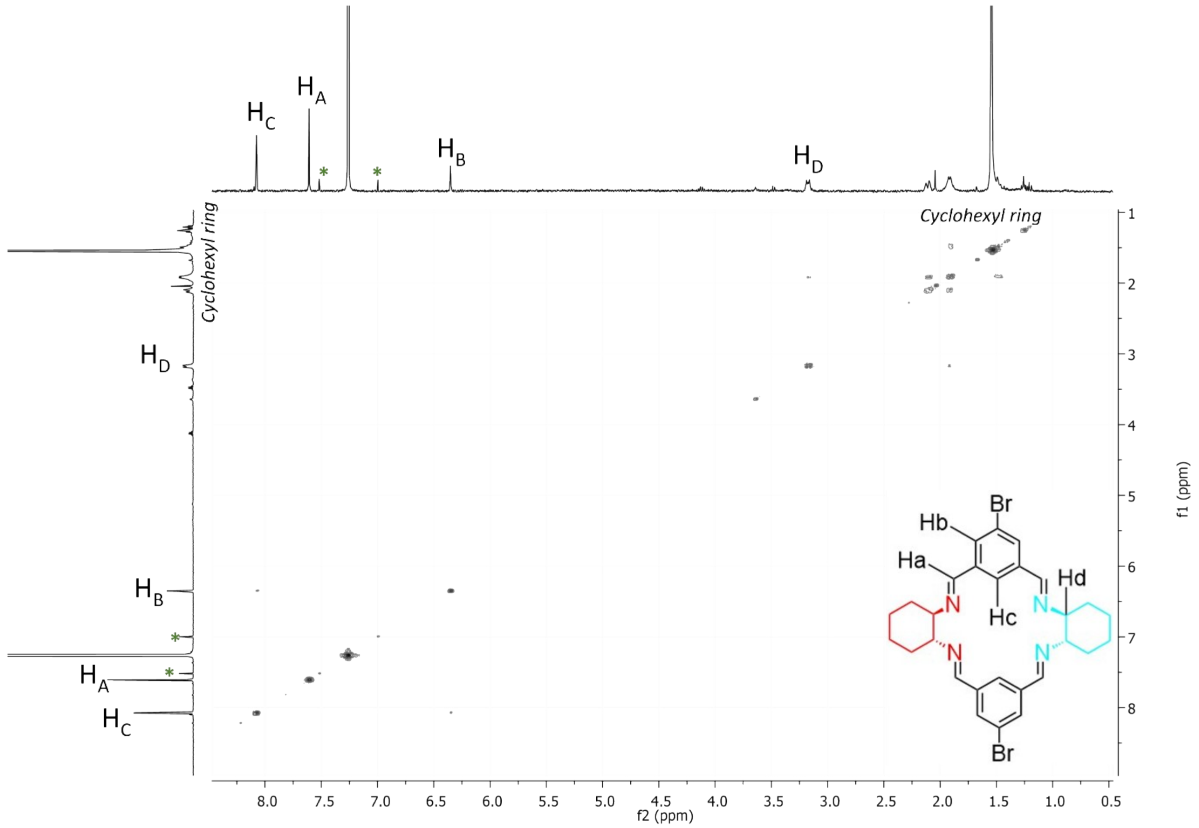

Figure S33: COSY ($CDCl_3$) spectrum for *meso*-[2+2]. * = spinning side bands.

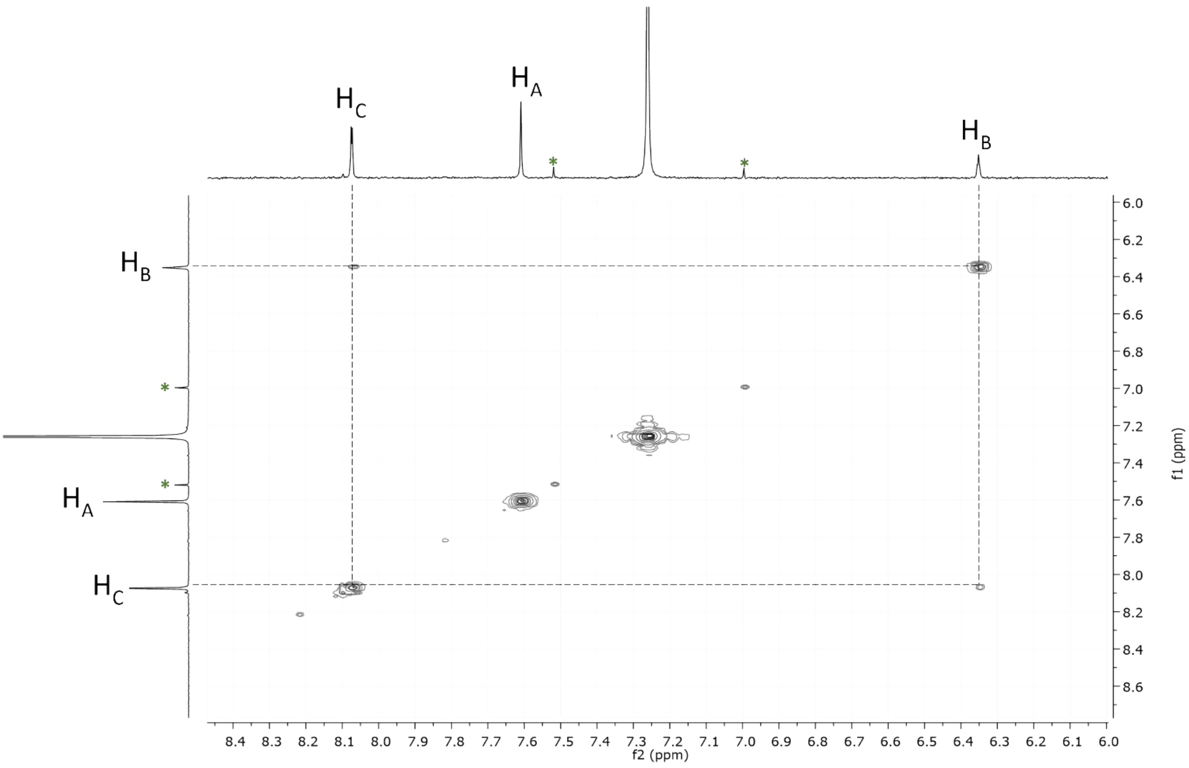

Figure S34: Aromatic region of COSY ($CDCl_3$) spectrum for *meso*-[2+2], 6.0 ppm – 8.4 ppm. * = Spinning side bands

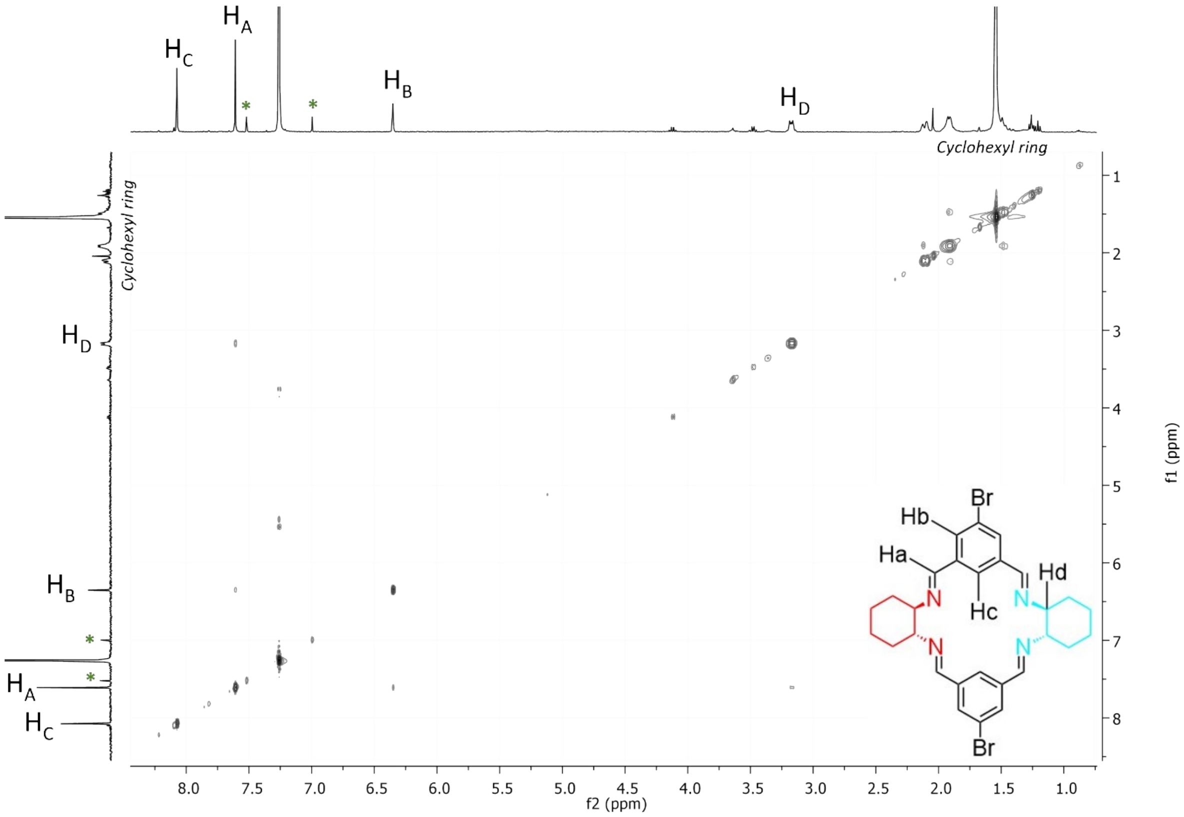

Figure S35: NOESY ($CDCl_3$) spectrum for *meso*-[2+2]. * = Spinning side band

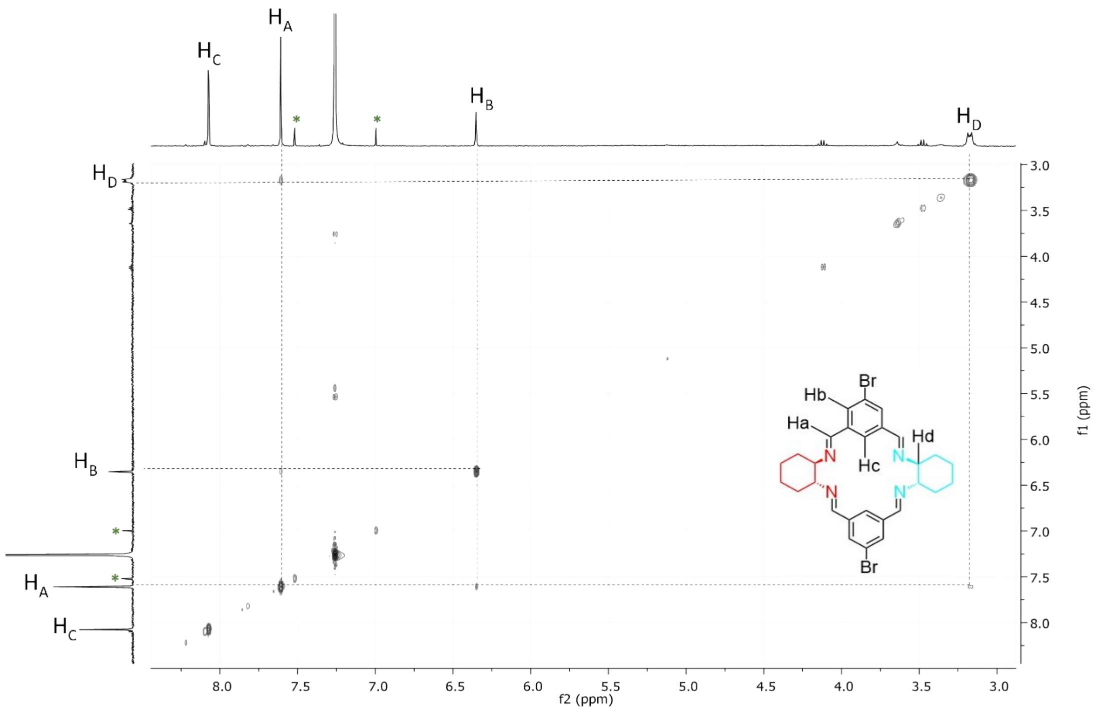

Figure S36: NOESY ($CDCl_3$) spectrum for *meso*-[2+2] – 3.0 ppm – 9.0 ppm.

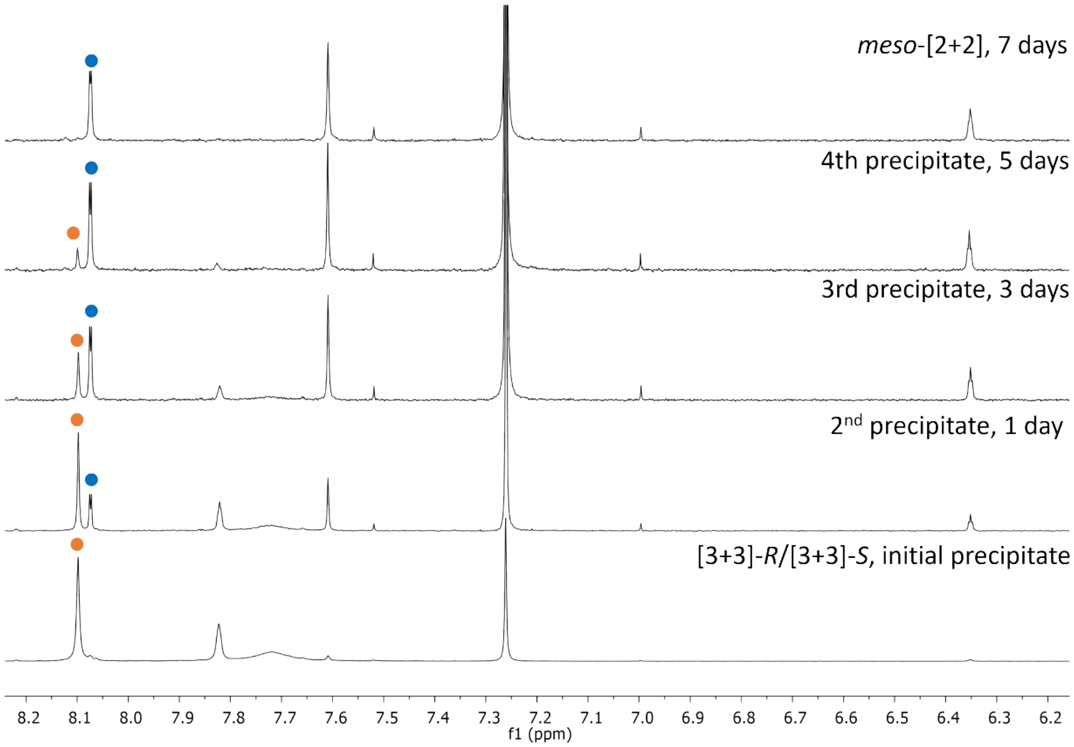

Figure S37: Stacked ^{1}H NMR ($CDCl_3$) spectra showing the formation of *meso*-[2+2] over time, alongside [3+3]-*R*/[3+3]-*S* as precipitate collected by filtration from the ethyl acetate solution. Orange: proton on [3+3]-*R*/[3+3]-*S*, blue: proton on *meso*-[2+2].

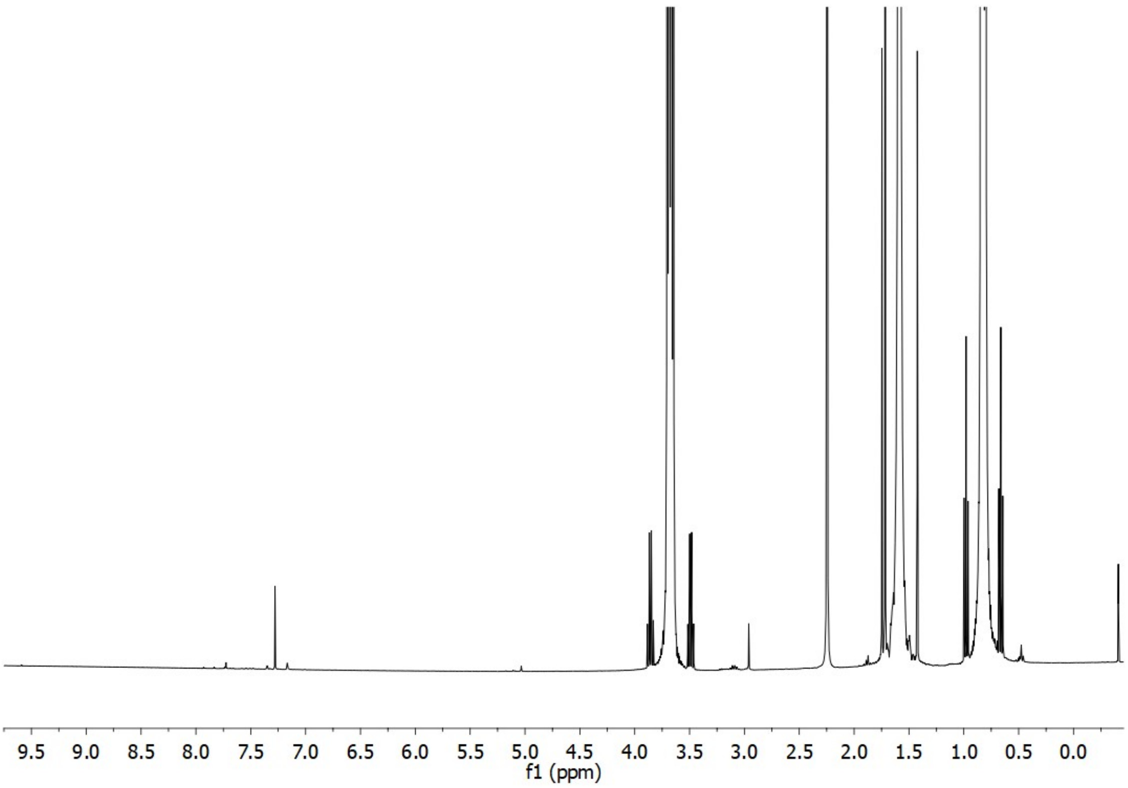

Figure S38: ^{1}H NMR ($CDCl_3$) spectrum of soluble racemic species left in ethyl acetate supernatant after isolation of *meso*-[2+2] and racemic [3+3] cocrystal, full ppm (see Figure S33 for magnified spectra of aromatic region). This is a dilute NMR, as can be seen by the large volume of ethyl acetate present in the sample, with $CDCl_3$ added to an aliquot of the ethyl acetate solution, as concentrating the sample was found to perturb the equilibrium; see Figure S40.

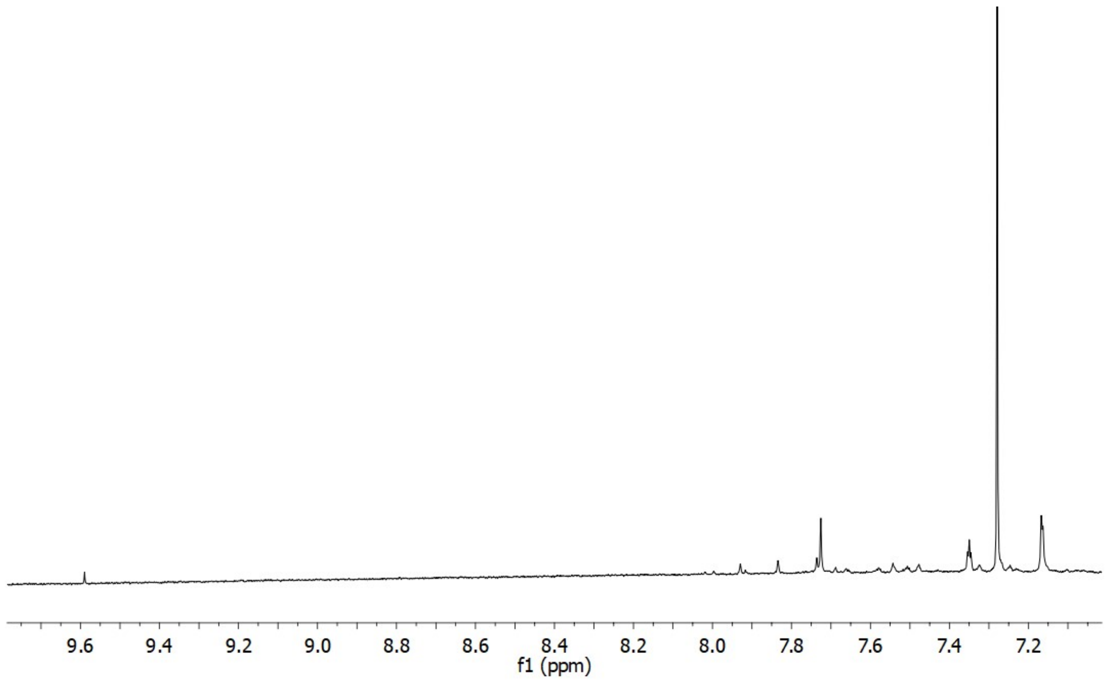

Figure S39: ^{1}H NMR ($CDCl_3$) spectrum of soluble racemic species left in ethyl acetate supernatant after isolation of *meso*-[2+2] and racemic [3+3] cocrystal, 7.0 ppm – 9.6 ppm.

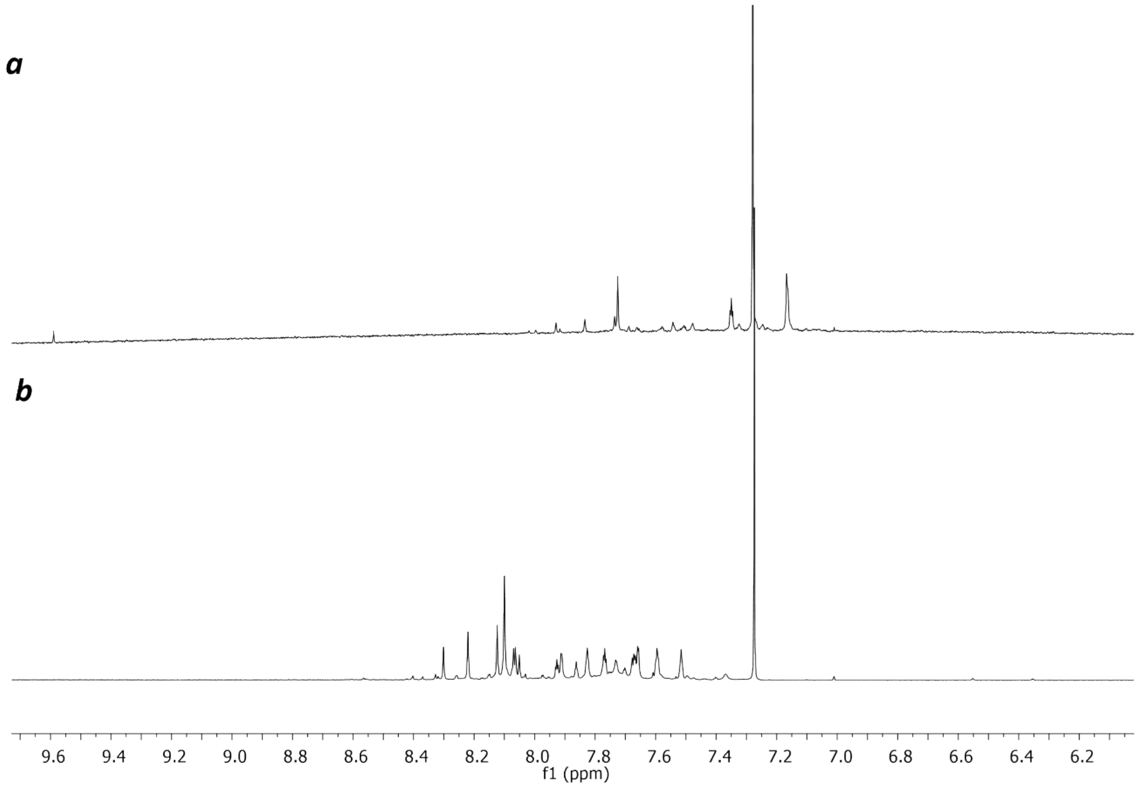

Figure S40: Stacked ^{1}H NMR ($CDCl_3$) spectra comparing the soluble racemic species left in ethyl acetate supernatant: (a) after trying to concentrate by removing solvent *in vacuo*; (b) without concentration, as in Figure S38.

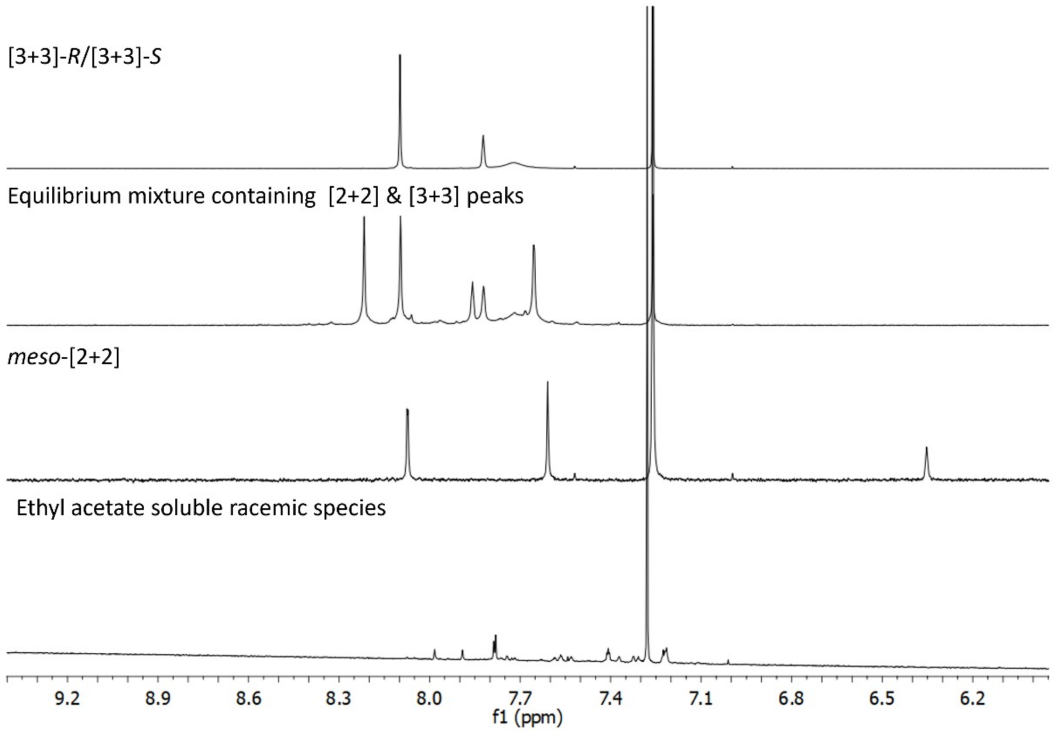

Figure S41: Stacked ^{1}H NMR ($CDCl_3$) spectra of soluble racemic species left in ethyl acetate supernatant, compared with proton peaks of *meso*-[2+2], isolated [3+3]-*R*/[3+3]-*S* co-crystal redissolved in $CDCl_3$, and the equilibrium mixture of chiral [2+2] and [3+3], 6.1 – 9.2 ppm. The NMR contains ethyl acetate, as removing under vacuum perturbed the equilibrium, so some peaks may be shifted.

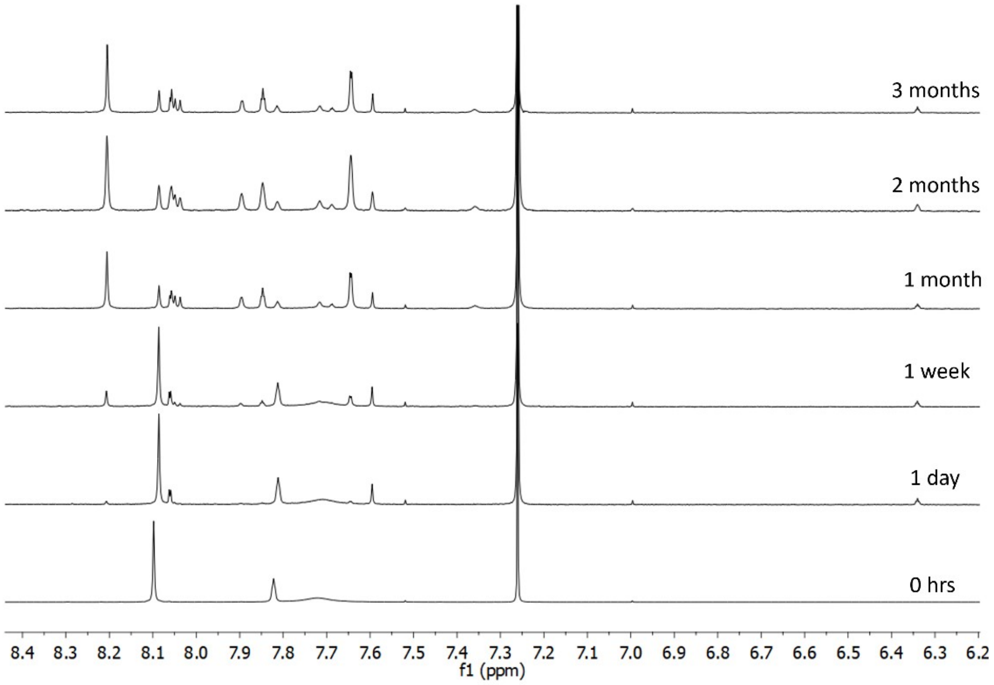

Figure S42: Stacked ^{1}H NMR ($CDCl_3$) spectra monitoring the stability of isolated [3+3]-*R*/[3+3]-*S* dissolved in $CDCl_3$ over 3 months, 6.2 ppm – 8.3 ppm.

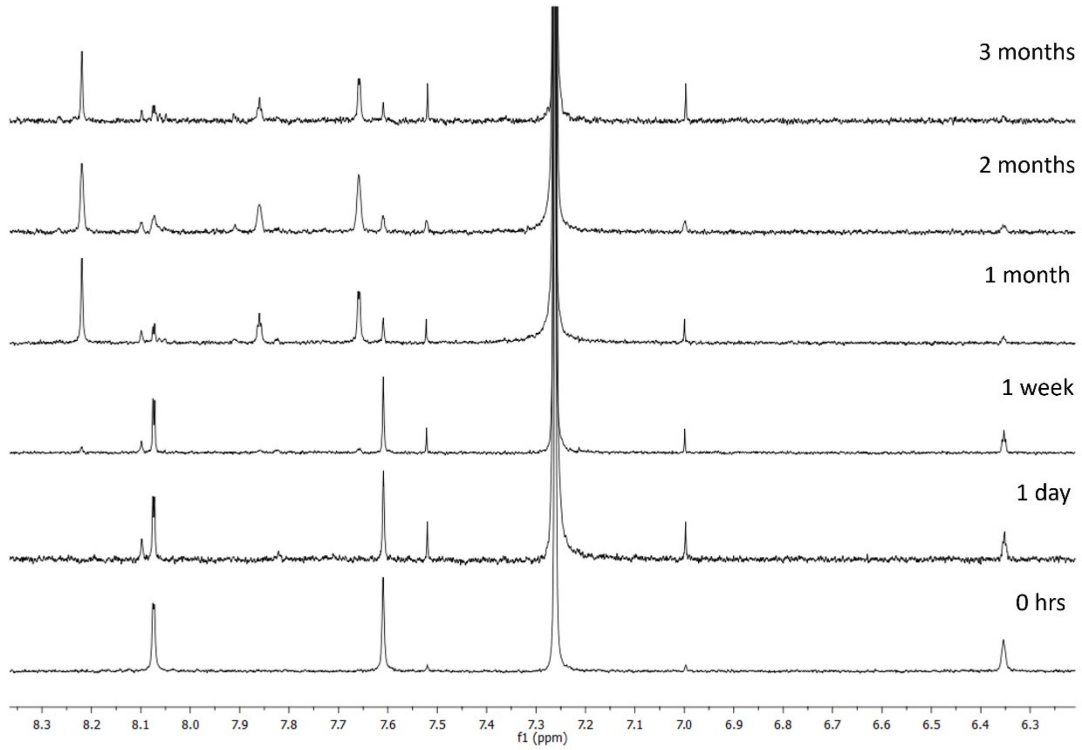

Figure S43: Stacked ^{1}H NMR ($CDCl_3$) spectra monitoring the stability of *meso*-[2+2] over 3 months, 6.2 ppm – 8.3 ppm.

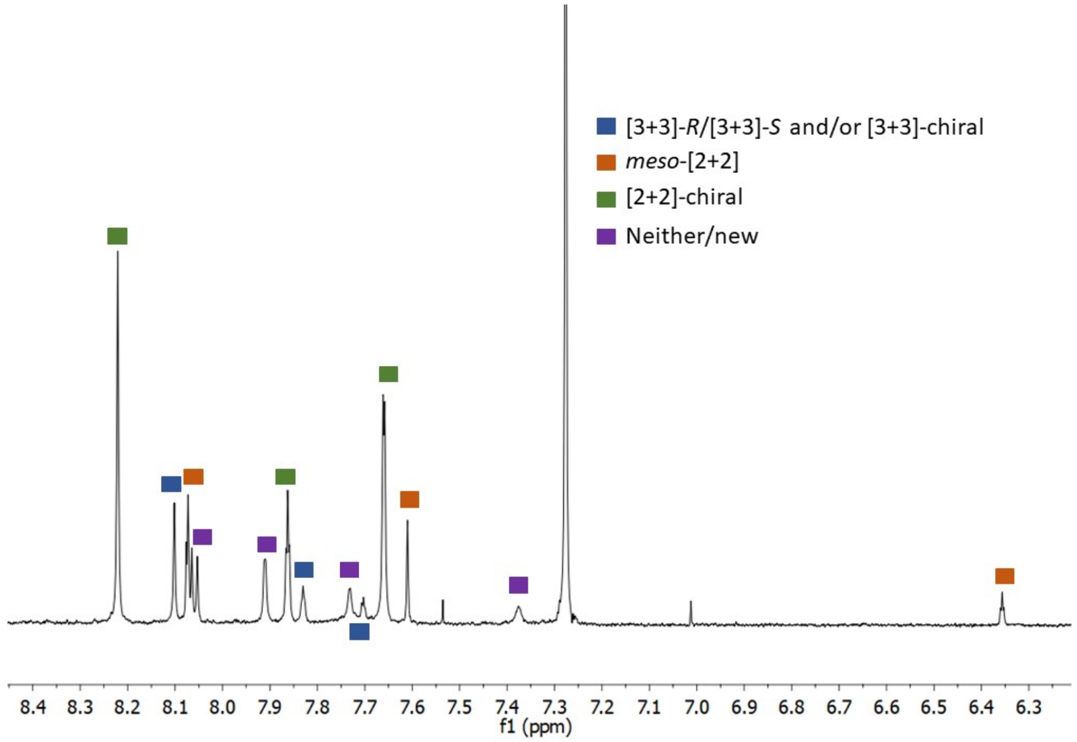

Figure S44: ^{1}H NMR ($CDCl_3$) assignments of the equilibrated species from species from *meso*-[2+2] left in solution, compared to other isolated macrocycles. 6.3 ppm – 8.4 ppm.

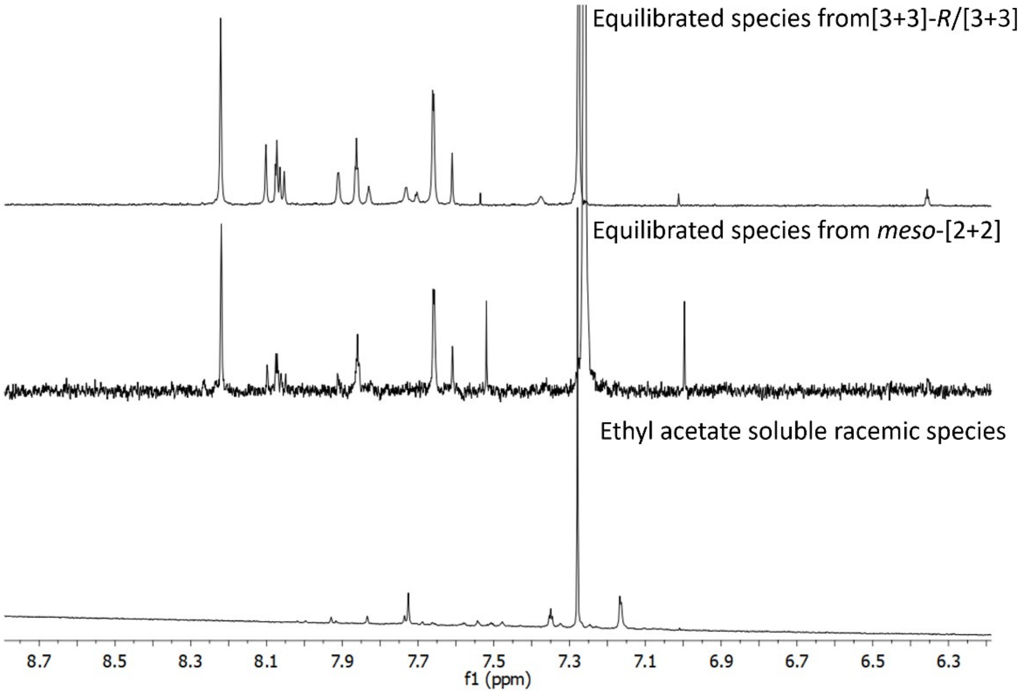

Figure S45: Stacked ^1H NMR ($CDCl_3$) spectra the equilibrated species from *meso*-[2+2], equilibrated species from [3+3]-*R/*[3+3]-S cocrystal and the ethyl acetate supernatant species, 6.2 ppm – 8.7 ppm.

To explore the effect of using racemic CHDA on equilibrium of cyclocondensation products formed, the synthesis was carried out as in Section 1.2.2 but with (±)-trans-1,2-Diaminocyclohexane. ^1H NMR of the crude reaction mixture (Figure S46) showed additional peaks alongside the species already identified by other heterochiral and homochiral experiments. We assume these peaks are associated with at least one other soluble racemic species, for example [3+3]-RRS as modelled in Figure S59, but due to the same issues discussed in the caption for Figure S38, any attempt to isolate these species would perturb the equilibrium so this was not followed up further.

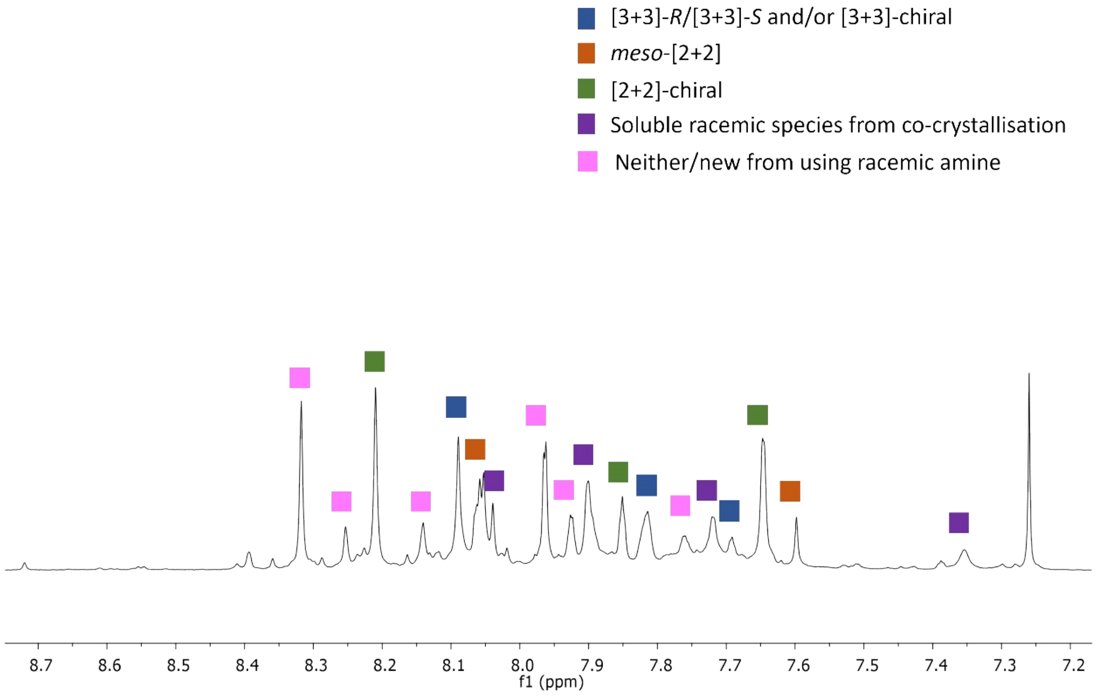

Figure S46: ^{1}H NMR ($CDCl_3$) assignments of the species formed when using the racemic amine for the cyclocondensation reaction, compared to other isolated macrocycles. 7.2 ppm – 8.7 ppm.

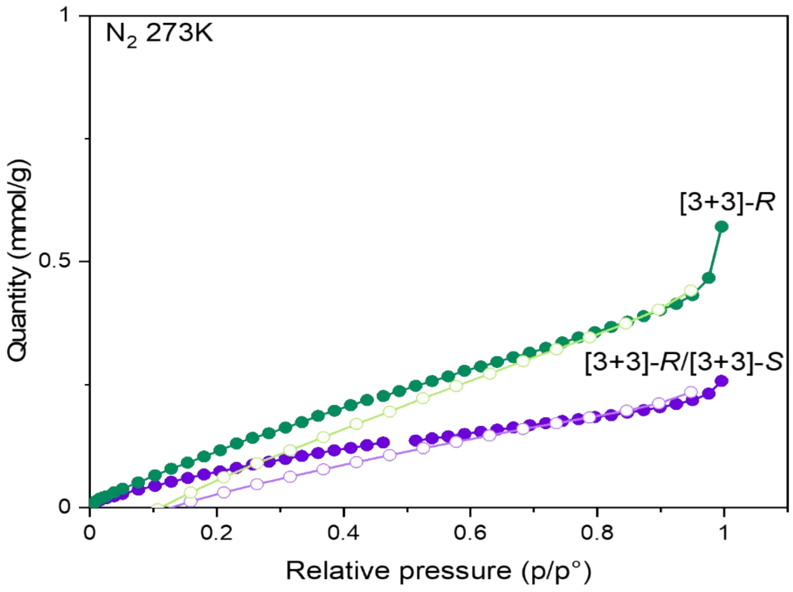

Figure S47: N2 gas sorption isotherm comparing homochiral [3+3]-R (green) and co-crystallised [3+3]-*R*/[3+3]-*S* (purple). Whole circles for adsorption, hollow circles for desorption. BET surface area for [3+3]-*R*: 16.2866 m²/g, [3+3]-*R*/[3+3]-*S*: 8.9865 ± 0.1659 m²/g.

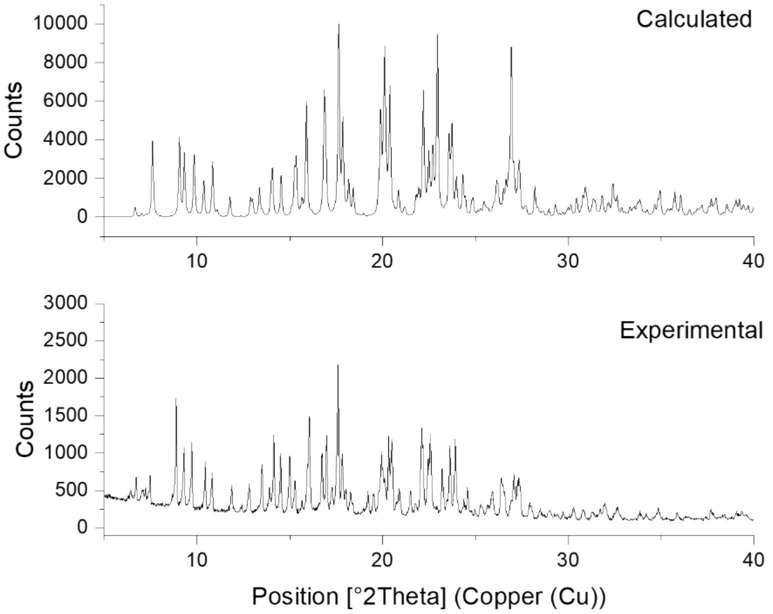

Figure S48: Stacked PXRD predicted from single crystal structure of [3+3]-*R*/[3+3]-*S* co-crystal obtained from ethyl acetate/hexane (upper) and experimental PXRD data of [3+3]-*R*/[3+3]-*S* co-crystal (lower). Experimental data obtained from recrystallisation of equimolar *R,R*- and *S,S*- equilibrium mixture from hot ethyl acetate, initial precipitate.

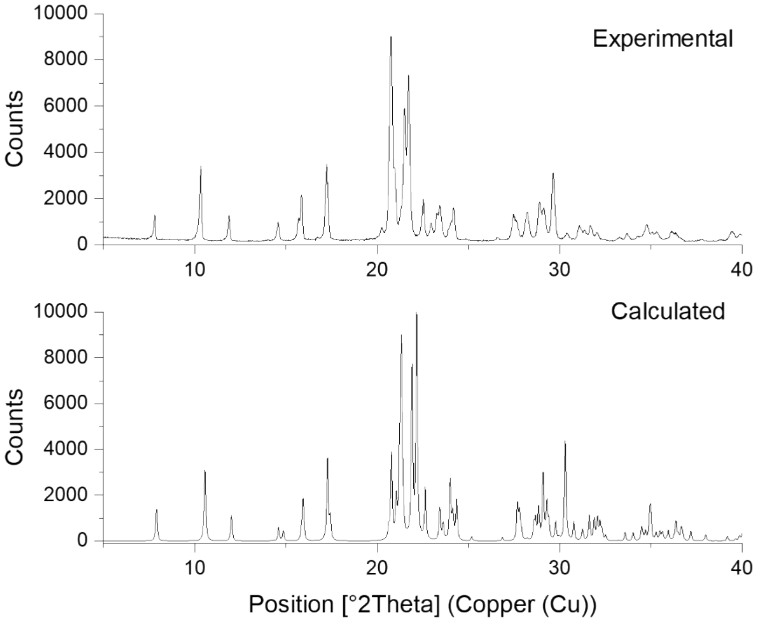

Figure S49: Stacked PXRD predicted from single crystal structure of *meso*-[2+2] obtained from DCM/1,4-dioxane (lower) and experimental PXRD data of *meso*-[2+2] (upper). Experimental data obtained from recrystallisation of equimolar *R,R*- and *S,S*- equilibrium mixture from hot ethyl acetate; 5th precipitation after 7 days.

1.7 Diffusion NMR for bromoisotrianglimine

All measurements were carried out using a 5 mm indirect detection probe equipped with z-gradient coil producing a nominal maximum gradient of 48 G/cm. Diffusion data was collected using the Bruker longitudinal eddy current delay (LED) bipolar gradient pulse sequence (ledbpgp2s). A diffusion encoding pulse (δ) of length 2.5 ms and a diffusion delay (Δ) 0.05 s was used. Gradient amplitudes were equally spaced between 0.963 G/cm and 45.742 G/cm. Each FID was acquired using 32k data points. All experiments were carried out at a nominal probe temperature of 298 K, with and air flow of 600 $m^3 min^{-1}$.

Diffusion coefficients were calculated from signal intensities using the Skejskal-Tanner equation using Bruker Dynamics Center:[3]

$$I = I_0 e^{-\gamma^2 G^2 \delta^2 \left(\Delta - \frac{\delta}{3}\right) D}$$

Where I is the signal intensity, I_0 is the signal intensity at a gradient strength of zero, G is the gradient strength, and D is the diffusion coefficient, ɣ is the gyromagnetic ratio, δ is the gradient pulse length and Δ is the diffusion time.

Table 2 Chemical shifts and diffusion coefficients of peaks for each species in the brominated equilibrium mixture containing the [2+2] and [3+3] macrocycles, 298 K

Species	Chemical Shift (ppm)	Diffusion Coefficient (m^2/s)	Average Diffusion Coefficient (m^2/s)
A	8.22	6.67×10^{-10}	
A	7.86	6.61×10^{-10}	6.61×10^{-10}
A	7.63	6.56×10^{-10}	
B	8.10	5.48×10^{-10}	
B	7.82	5.55×10^{-10}	5.48×10^{-10}
B	7.72	5.37×10^{-10}	
B	7.66	5.51×10^{-10}	

1.7.1 Decay Curves

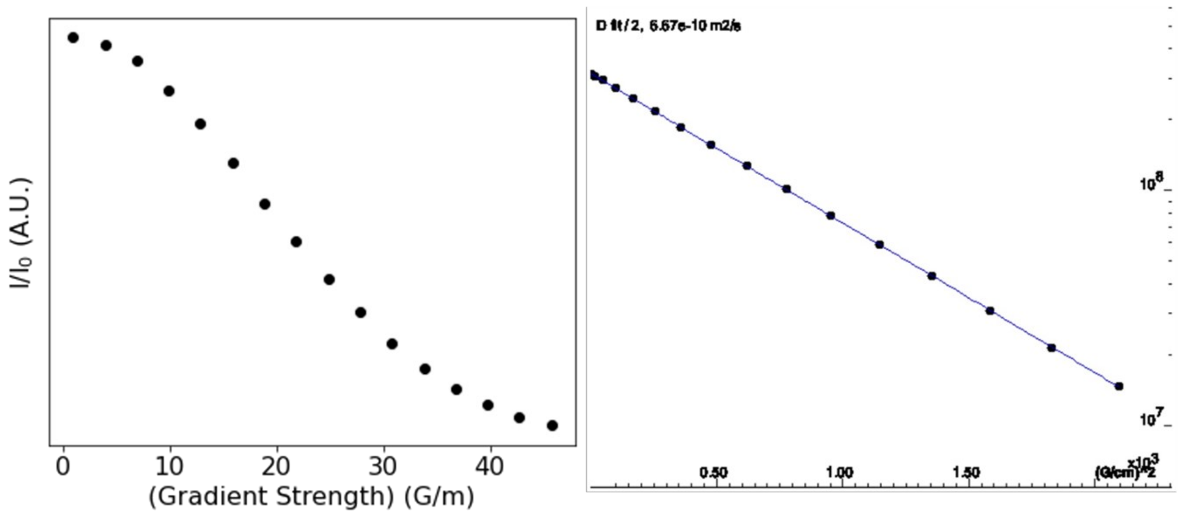

Figure S50: Diffusion decay curves (left) and Skejskal-Tanner fit (right) for peak at 8.22 ppm for bromoisotrianglimine, belonging to the [2+2] macrocycle.

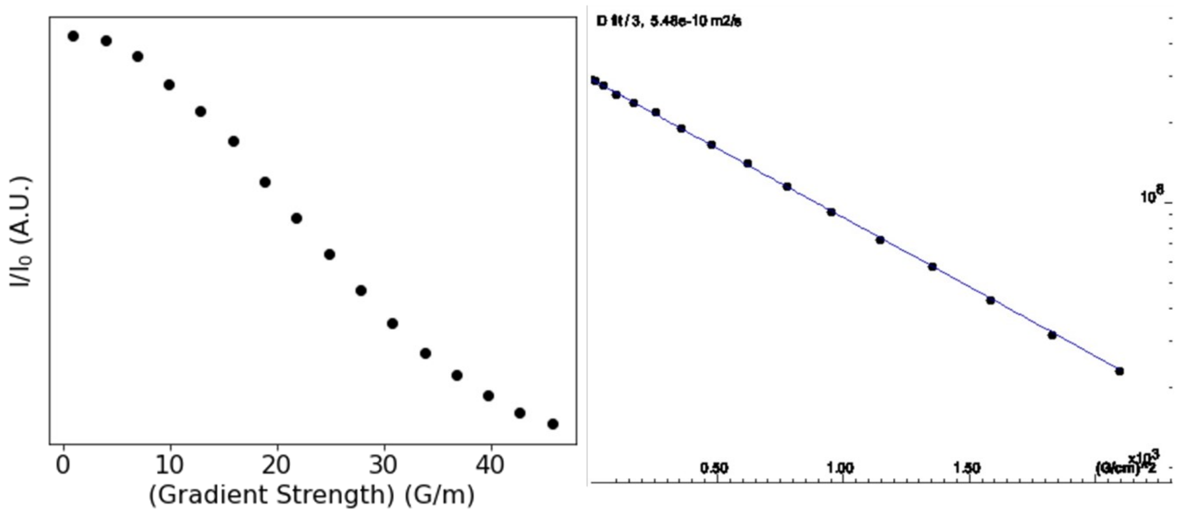

Figure S51: Diffusion decay curves (left) and Skejskal-Tanner fit (right) for peak at 8.10 ppm for bromoisotrianglimine, belonging to the [3+3] macrocycle.

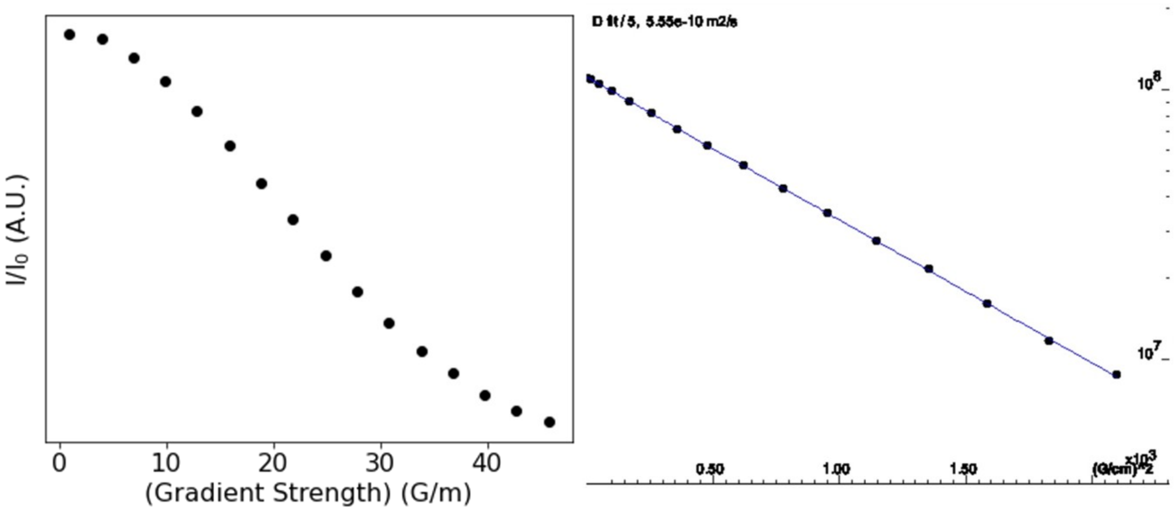

Figure S52: Diffusion decay curves (left) and Skejskal-Tanner fit (right) for peak at 7.86 ppm for bromoisotrianglimine, belonging to the [2+2] macrocycle.

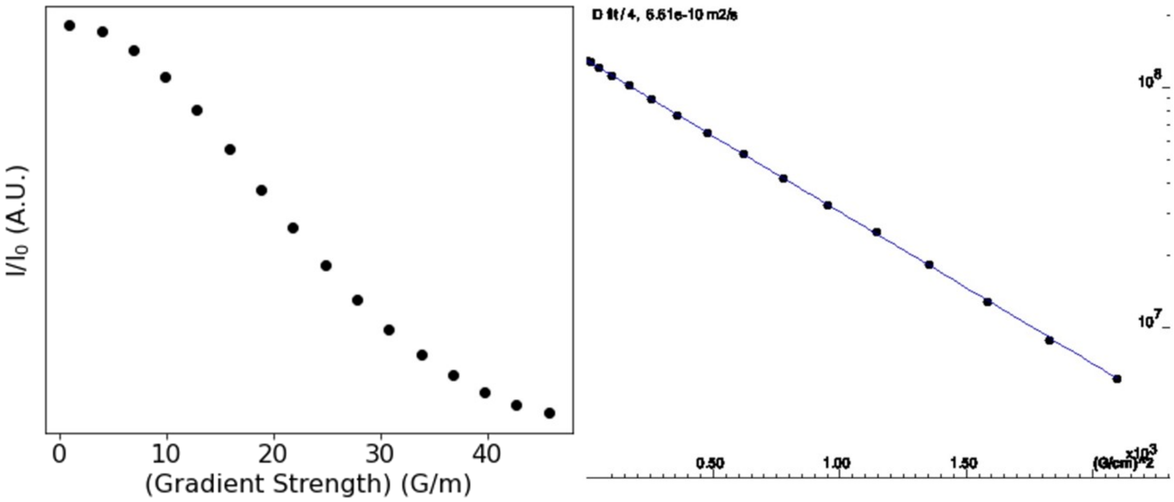

Figure S53: Diffusion decay curves (left) and Skejskal-Tanner fit (right) for peak at 7.82 ppm for bromoisotrianglimine, belonging to the [3+3] macrocycle.

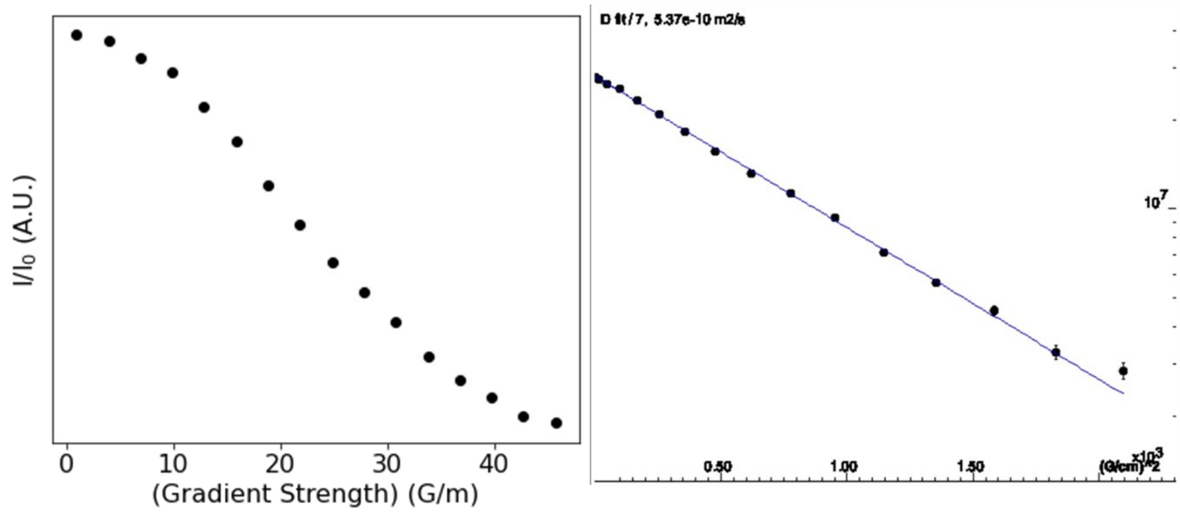

Figure S54: Diffusion decay curves (left) and Skejskal-Tanner fit (right) for peak at 7.72 ppm for bromoisotrianglimine, belonging to the [3+3] macrocycle.

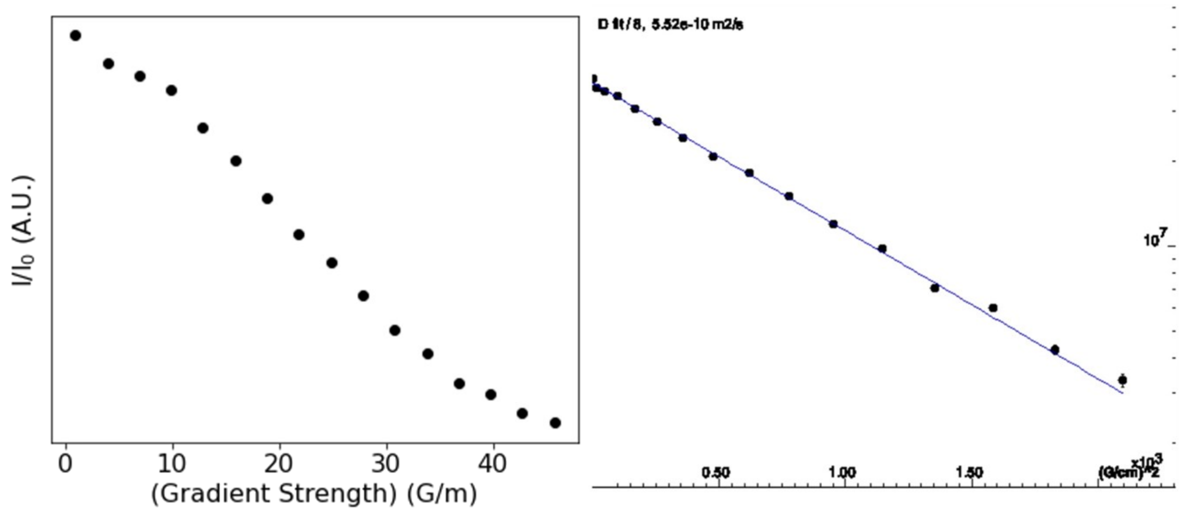

Figure S55: Diffusion decay curves (left) and Skejskal-Tanner fit (right) for peak at 7.66 ppm for bromoisotrianglimine, belonging to the [3+3] macrocycle.

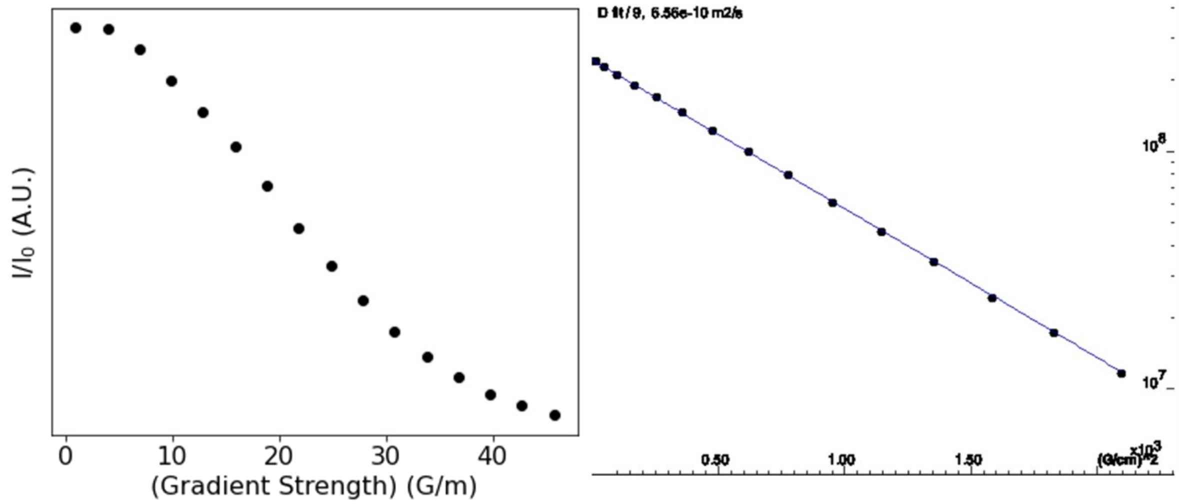

Figure S56: Diffusion decay curves (left) and Skejskal-Tanner fit (right) for peak at 7.63 ppm for bromoisotrianglimine, belonging to the [2+2] macrocycle.

2. Single Crystal data

All crystal structures reported were obtained by vapor diffusion, using 15 mg of material dissolved in the chosen solvent (1 mL). See the following pages for crystallographic tables.

2.1 Crystallographic tables

Table 3: Crystallographic table 1

**Form**	[2+2]-*meso*	[3+3]-*R*/[3+3]-*S*	[3+3]-*R*/[3+3]-*S*	[3+3]-*R*	[3+3]-*R*
**Solvate**	Solvent free	*n*-hexane	DCM	toluene	xylenes
**Chemical formula moiety**	$C_{28}H_{30}Br_2N_4$	$C_{42}H_{45}Br_3N_6$, $C_{2.1}H_{4.9}$	$C_{42}H_{45}Br_3N_6$, $C_{0.95}H_{1.90}Cl_{1.90}$	$C_{42}H_{45}Br_3N_6$, $C_7H_{7.65}$	$C_{42}H_{45}Br_3N_6$, C_8H_{10}
**Chemical formula sum**	$C_{28}H_{30}Br_2N_4$	$C_{44.10}H_{49.89}Br_3N_6$	$C_{42.95}H_{46.90}Br_3Cl_{1.90}N_6$	$C_{49}H_{52.65}Br_3N_6$	$C_{50}H_{55}Br_3N_6$
Mr	582.38	903.67	954.25	965.35	979.73
**Crystal system**	Triclinic	Monoclinic	Monoclinic	Monoclinic	Monoclinic
**Space group**	*P*-1	*I*2/*a*	*C*2/*c*	$P2_1$	$P2_1$
Z'	0.5	1	1	1	1
Z	1	8	8	2	2
***a* (Å)**	6.4446(2)	23.6941(11)	31.5459(17)	11.0041(2)	11.0644(2)
***b* (Å)**	8.9992(6)	14.3036(7)	14.3588(3)	14.3125(2)	14.2714(2)
***c* (Å)**	11.3593(4)	27.0164(13)	23.7813(12)	15.5247(3)	15.6724(3)
**α (°)**	99.016(4)	90	90	90	90
**β (°)**	92.137(3)	101.598(4)	123.990(8)	109.692(2)	109.809(2)
**γ (°)**	109.139(5)	90	90	90	90
***V* (Å³)**	611.91(5)	8969.2(8)	8931.4(10)	2302.09(8)	2328.30(7)
***μ* (mm⁻¹)**	3.337	2.734	2.860	2.668	2.639
**Crystal size (mm)**	0.05 x 0.21 x 0.31	0.03 x 0.05 x 0.19	0.02 x 0.04 x 0.14	0.10 x 0.11 x 0.20	0.08 x 0.11 x 0.26
**Temperature (K)**	100.01(10)	100.01(10)	100.01(10)	100.01(11)	100.01(10)
**Radiation type**	Mo *K*a, λ = 0.71073	Mo *K*a, λ = 0.71073	Mo *K*a, λ = 0.71073	Mo *K*a, λ = 0.71073	Mo *K*a, λ = 0.71073
**Absorption correction**	Multi-scan	Multi-scan	Multi-scan	Multi-scan	Multi-scan
**T_{min}, T_{max}**	0.609, 1.000	0.717, 1.000	0.769, 1.000	0.881, 1.000	0.820, 1.000
**F (000)**	296	3692	3871	987.0	1004
**No. of measured, independent and observed [*I* > 2σ(*I*)] reflections**	3047, 3047, 2901	42323, 8202, 4889	52536, 10122, 7097	47561, 11043, 10218	67243, 11279, 10491
**R_{int}, R_{sigma}**	0.0293, 0.0214	0.1108, 0.0985	0.0761, 0.0716	0.0358, 0.0330	0.0341, 0.0270
**Θ_{max} (°)**	29.557	25.389	27.503	28.297	28.303
**Data / restraints / parameters**	3047 / 0 / 155	8202 / 2 / 474	10122 / 20 / 514	11043 / 29 / 563	11279 / 66 / 612

Table 4: Crystallographic table 2

Form	[3+3]-*R*	[3+3]-*R*	[3+3]-*R*	[3+3]-*S*	[4+4]-*S*
**Solvate**	H_2O	MeOH	desolvated	MeOH	1,4-dioxane
**Chemical formula moiety**	$C_{42}H_{45}Br_3N_6$, $H_3O_{2.29}$	$C_{42}H_{45}Br_3N_6$, $C_{1.25}H_5O_{1.25}$	$C_{42}H_{45}Br_3N_6$	$C_{42}H_{45}Br_3N_6$, $C_{1.26}H_{5.05}O_{1.26}$	$C_{56}H_{60}Br_4N_8$, $C_{20}H_{40}O_{10}$
**Chemical formula sum**	$C_{42}H_{48}Br_3N_6O_{2.29}$	$C_{43.24}H_{49.97}Br_3N_6O_{1.25}$	$C_{42}H_{45}Br_3N_6$	$C_{43.26}H_{50.05}Br_3N_6O_{1.26}$	$C_{76}H_{100}Br_4N_8O_{10}$
Mr	913.15	913.45	873.57	914.02	1605.27
**Crystal system**	Monoclinic	Monoclinic	Monoclinic	Monoclinic	Orthorhombic
**Space group**	$P2_1$	$P2_1$	$P2_1$	$P2_1$	$I2_12_12_1$
Z'	2	2	2	2	0.5
Z	4	4	4	4	4
***a* (Å)**	12.5590(4)	14.8232(7)	14.5723(3)	14.8142(3)	16.3450(7)
***b* (Å)**	23.3781(6)	19.6544(10)	18.9659(3)	19.7153(4)	17.4893(6)
***c* (Å)**	16.2798(5)	14.9442(7)	14.9238(2)	14.9459(3)	26.7421(11)
**α (°)**	90	90	90	90	90
**β (°)**	102.547(3)	92.335(2)	91.9080(10)	92.298(2)	90
**γ (°)**	90	90	90	90	90
***V* (Å^3)**	4665.7(2)	4350.2(4)	4122.31(12)	4361.68(15)	7644.6(5)
***μ* (mm^{-1})**	2.632	2.822	2.972	2.814	2.167
**Crystal size (mm)**	0.05 x 0.06 x 0.17	0.03 x 0.19 x 0.36	0.13 x 0.25 x 0.38	0.03 x 0.12 x 0.18	0.14 x 0.20 x 0.28
**Temperature (K)**	100.01(13)	149.99(10)	100.01(10)	150.01(10)	100.01(11)
**Radiation type**	Mo *K*a, λ = 0.71073	Mo *K*a, λ = 0.71073	Mo *K*a, λ = 0.71073	Mo *K*a, λ = 0.71073	Mo *K*a, λ = 0.71073
**Absorption correction**	Multi-scan	Multi-scan	Gaussian	Multi-scan	Multi-scan
**T_{min}, T_{max}**	0.796, 1.000	0.732, 1.000	0.171, 1.000	0.774, 1.000	0.500, 1.000
**F (000)**	1861.0	1866	1776	1867	3328
**No. of measured, independent and observed [I > 2σ(I)] reflections**	55071, 18957, 14728	66078, 17694, 13970	145655, 20267, 17354	73302, 19721, 14387	27440, 7824, 5437
**R_{int}, R_{sigma}**	0.0402, 0.0559	0.0706, 0.0729	0.0600, 0.0487	0.0558, 0.0637	0.0630, 0.0718
**$Θ_{max}$ (°)**	26.422	26.440	28.321	27.511	26.414
**Data / restraints / parameters**	18957 / 1 / 975	17694 / 1 / 982	20267 / 1 / 919	19721 / 1 / 982	7824 / 118 / 480
**Goodness of fit, *S***	1.051	1.040	1.066	1.007	1.040

2.2 Additional Information for single crystal data

[3+3]-*R* desolvated:

A full structural solution of [3+3]-*R* obtained from MeOH and THF revealed a topologically identical lattice to [3+3]-*R* solvated with H_2O, in which a modulation along *b* permits the inclusion of three discrete near fully occupied ordered MeOH molecules of crystallisation. This results in a small slippage of the pi-pi offset bromobenzene stacks within associating dimers, reduced by ca. 0.3 Å, however the centroid-to-centroid distance is effectively unchanged at 3.634 (*cf*. 3.671 Å)[33].

Desolvation was attempted in situ via warming to 375 K: A crystal freshly extracted from the mother liquor was warmed on the diffractometer to 375 K, and over the course of 0.5 h a reduction in the crystallographic *b* axis of 0.9 Å manifested. Cooling back down to 100 K, a full structure determination was performed which confirmed the loss of MeOH from the crystal and clarified through a subsequent SQUEEZE analysis which calculated only a residual 6 electrons in the model per cell, equating to essentially zero remaining solvent presence above the noise level. The cell parameters exhibit only a modest isotropic unit cell contraction of 100 Å^3 compared to the as-crystallised solvent-free structure reported by Kwit *et al* which was instead carried out at ambient temperature.

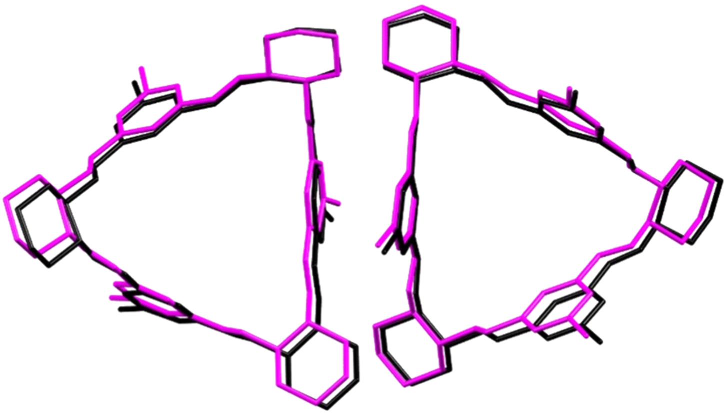

S57: Methanolic solvated (magenta) and desolvated (black) [3+3]

3 Molecular Modelling

Conformer generation for macrocycles

Starting from the InChI identifiers of all the molecules studied (macrocycles, aldehydes, amines, and water), we embedded 1000 conformers using the ETKDGv3[4] algorithm as implemented in RDkit[5] 2022.03.2. The structures were minimised using the MMFF force-field and the lowest-energy conformers where all imine bonds had *E* geometry were used for subsequent calculations. With the embedded structures at hand, CREST[6,7] was used with GFN2-xTB[8,9] semi-empirical functional and the built-in analytical linearized Poisson-Boltzmann (ALPB) solvation model for chloroform to perform conformational searches. Redundant conformers were removed with an RMSD threshold of 0.5 A using the sPyRMSD[10] package. Such symmetry corrected RMSD calculations were necessary for the highly symmetric macrocycles. Each unique conformer generated with CREST was optimised in Orca 5.0.1.[11] using the B97-3c[12] functional and confirmed to be a true minimum with frequency calculations. The calculations used atom-pairwise dispersion correction[13] with the Becke-Johnson damping scheme (D3BJ)[14] and the universal solvent model based on density (SMD)[15] for implicit treatment of chloroform. Entropy contributions were computed according to the QRRHO[16] model. Lowest-energy conformer of each structure from the B97-3c (Figures S44-S46) was used for single-point calculations at higher levels of theory.

Conformer generation for [4+4]-*R* macrocycle

Conformer generation following the above procedure yielded a collapsed structure for the [4+4]-*R* macrocycle. As it has been observed in the solid state as an inclusion complex with dioxane, a semi-open conformation from the CREST search was identified (at 11.5 kJ/mol relative to the identified minimum, see Figure S46) and a single molecule of dioxane was manually placed inside the cavity. The structure was optimised at GFN2-xTB level of theory and used for a conformational search in CREST using the non-covalent interactions mode with identical settings as above. Resulting conformers were filtered to only include structures with dioxane occupying the cavity formed by the macrocycle, resulting in eight unique conformations. The lowest energy structure is depicted in Figure S47.

Single-point calculations and formation energy calculations

Final energetics were obtained with single point energies at a DFT level of theory. For all reported calculations, the def2-QZVP basis[17] set with the def2/J auxiliary basis[18] set were used. Results were compared between the M06-2X[19], PBE0[20], PW6B95,[21] ωB97X-V[22] (as well

as the D3BJ-corrected variant ωB97X-D3), and ωB97M-V[23]. The functionals were corrected for dispersion either following Grimme's D3 scheme or using the non-local VV10 correction.[24] Final energies were calculated both in vacuum and with SMD chloroform solvation. Formation energies were obtained following standard thermodynamic relationships. Electronic formation energy is simply the difference in final single point energies calculated at the DFT level of theory. As thermal corrections depend predominantly on geometry, they were extracted from the relatively cheap frequency calculation using the B97-3c method. All thermochemical properties were calculated at 298.15K. Enthalpy is calculated as U + kB*T, where U is the inner energy, i.e., the sum of the total electronic single point energy, the zero-point energy and the thermal energy due to vibrations, rotations, and translations. Entropy contributions are the sum of electronic, vibrational, rotational and translational entropy. Rotational entropy was calculated assuming symmetry number 1 for the macrocycle. The symmetry numbers for aldehyde (C_{2v}), amine (C_2) and water (C_{2v}) are all 2. Differences in the Gibbs free energies of formation for different symmetry numbers for the macrocycles are within 5 kJ/mol and are negligible when scaled per imine bond formed.

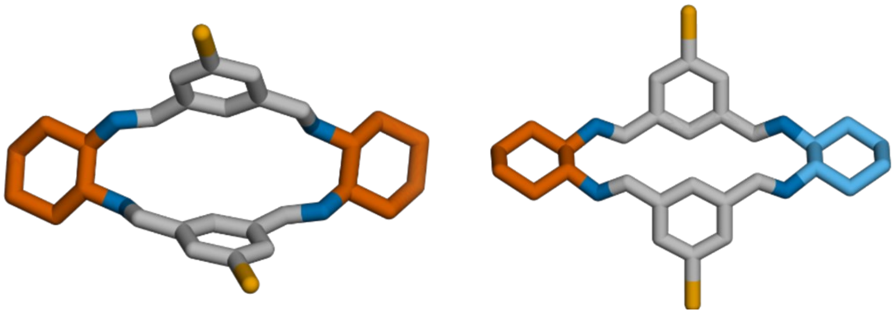

Figure S58: Lowest energy conformation of modelled [2+2] macrocycles from GFN2-xTB CREST search, further refined at B97-3c level of theory; [2+2]-*R* (left), labelled [2+2]-RR in Figure 4, and *meso*-[2+2] (right), labelled [2+2]-RS in Figure 4.

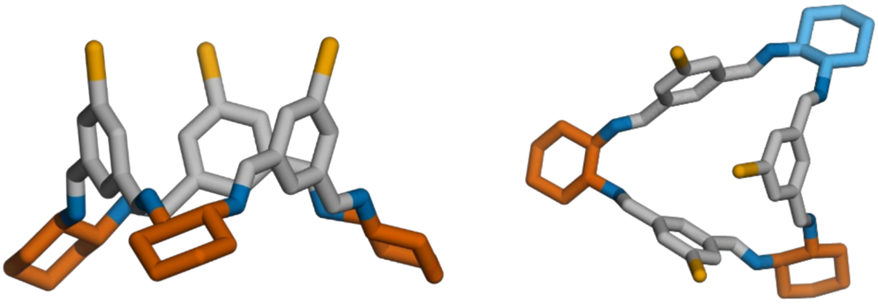

Figure S59: Lowest energy conformation of modelled [3+3] macrocycles from GFN2-xTB CREST search, further refined at B97-3c level of theory; [3+3]-*R* (left), labelled [3+3]-RRR in Figure 4, and [3+3]-*RRS* (right), labelled [3+3]-RRS in Figure 4.

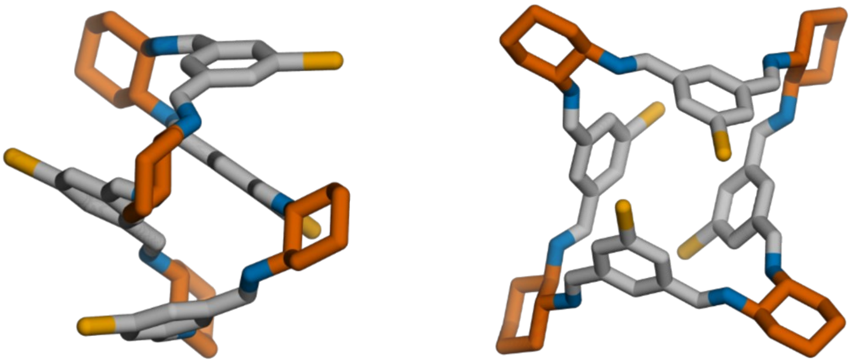

Figure S60: Representative conformations of the modelled [4+4]-*R* macrocycle from GFN2-xTB CREST search, further refined at B97-3c level of theory; lowest-energy collapsed [4+4] structure (left); open [4+4] structure that is at 11.5 kJ/mol from the identified minimum (right).

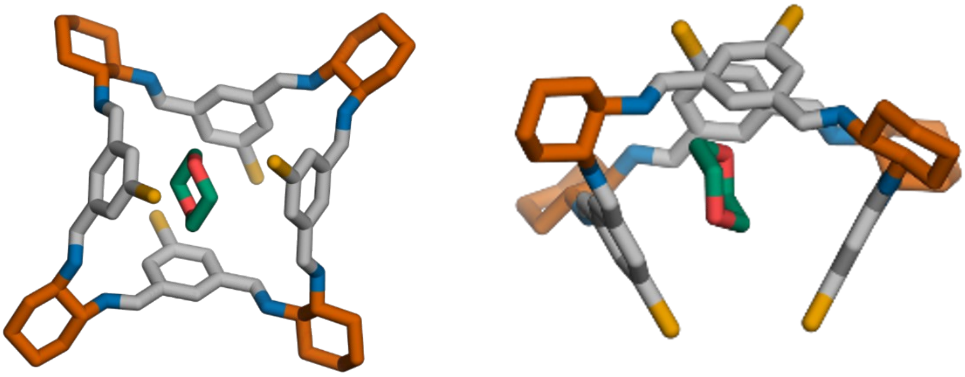

Figure S61: Lowest energy conformation with dioxane explicitly placed inside the [4+4] cavity macrocycle from GFN2-xTB CREST search in the non-covalent interactions mode; top view (left); side view (right).

4 References

1 A. Troć, J. Gajewy, W. Danikiewicz and M. Kwit, *Chem. - Eur. J.*, 2016, **22**, 13258–13264.

2 E. O. Stejskal and J. E. Tanner, *J. Chem. Phys*, 1965, **42**, 288–292.

3 S. Wang, J. Witek, G. A. Landrum and S. Riniker, *J. Chem. Inf. Model.*, 2020, **60**, 2044–2058.

4 RDKit: Open-source cheminformatics. https://www.rdkit.org, DOI:10.5281/ZENODO.7880616S.

5 Grimme, *J. Chem. Theory. Comput*., 2019, **15**, 2847–2862.

6 P. Pracht, F. Bohle and S. Grimme, *Phys. Chem. Chem. Phys*., 2020, **22**, 7169–7192.

7 C. Bannwarth, S. Ehlert and S. Grimme, *J. Chem. Theory. Comput.*, 2019, **15**, 1652–1671.

8 C. Bannwarth, E. Caldeweyher, S. Ehlert, A. Hansen, P. Pracht, J. Seibert, S. Spicher and S. Grimme, *WIREs Comput. Mol. Sci.*, 2021, **11**, e1493–e1493.

9 R. Meli and P. C. Biggin, *J. Cheminform.*, 2020, **12**, 1–7.

10 F. Neese, *Wiley. Interdiscip. Rev. Comput. Mol. Sci.*, 2022, **12**, e1606–e1606.

11 J. G. Brandenburg, C. Bannwarth, A. Hansen and S. Grimme, *J. Chem. Phys.*, 2018, **148**, 64104.

12 S. Grimme, S. Ehrlich and L. Goerigk, *J. Comput. Chem.*, 2011, **32**, 1456–1465.

13 S. Grimme, J. Antony, S. Ehrlich and H. Krieg, *J. Chem. Phys.*, 2010, **132**, 154104.

14 A. V. Marenich, C. J. Cramer and D. G. Truhlar, *J. Chem. Phys. B*, 2009, **113**, 6378–6396.

15 S. Grimme, *Chem. - Eur. J.*, 2012, **18**, 9955–9964.

16 F. Weigend and R. Ahlrichs, *Phys. Chem. Chem. Phys*., 2005, **7**, 3297–3305.

17 M. K. Armbruster, W. Klopper and F. Weigend, *Phys. Chem. Chem. Phys.*, 2006, **8**, 4862–4865.

18 Y. Zhao and D. G. Truhlar, *Theor. Chem. Acc.,* 2008, **120**, 215–241.

19 C. Adamo and V. Barone, *J. Chem. Phys*., 1999, **110**, 6158–6170.

20 Y. Zhao and D. G. Truhlar, *J. Chem. Phys. A*, 2005, **109**, 5656–5667.

21 N. Mardirossian and M. Head-Gordon, *Phys. Chem. Chem. Phys.*, 2014, **16**, 9904.

22 N. Mardirossian and M. Head-Gordon, *Phys. Chem. Chem. Phys.*, 2016, **144**, 214110.

23 O. A. Vydrov and T. Van Voorhis, *J. Chem. Phys.*, 2010, **133**, 244103.
